# Synergism of Light-Induced [4 + 4] Cycloaddition and Electron Transfer Toward Switchable Photoluminescence and Single-Molecule Magnet Behavior in a Dy_4_ Cubane

**DOI:** 10.34133/research.0411

**Published:** 2024-07-05

**Authors:** Yu-Han Wang, Zhen-Ni Gao, Shuai Liang, Jie Li, Wu-Ji Wei, Song-De Han, Yi-Quan Zhang, Ji-Xiang Hu, Guo-Ming Wang

**Affiliations:** ^1^ College of Chemistry and Chemical Engineering, Qingdao University, Qingdao 266071, China.; ^2^ Jiangsu Key Laboratory for NSLSCS, School of Physical Science and Technology, Nanjing Normal University, Nanjing 210023, China.

## Abstract

Molecular materials possessing switchable magneto-optical properties are of great interest due to their potential applications in spintronics and molecular devices. However, switching their photoluminescence (PL) and single-molecule magnet (SMM) behavior via light-induced structural changes still constitutes a formidable challenge. Here, a series of cubane structures were synthesized via self-assembly of 9-anthracene carboxylic acid (HAC) and rare-earth ions. All complexes exhibited obvious photochromic phenomena and complete PL quenching upon Xe lamp irradiation, which were realized via the synergistic effect of photogenerated radicals and [4 + 4] photocycloaddition of the AC components. The quenched PL showed the largest fluorescence intensity change (99.72%) in electron-transfer photochromic materials. A reversible decoloration process was realized via mechanical grinding, which is unexpectedly in the electron-transfer photochromic materials. Importantly, an SMM behavior of the Dy analog was observed after room-temperature irradiation due to the photocycloaddition of AC ligands and the photogenerated stable radicals changed the electrostatic ligand field and magnetic coupling. Moreover, based on the remarkably photochromic and photoluminescent properties of these compounds, 2 demos were applied to support their application in information anti-counterfeiting and inkless printing. This work, for the first time utilizing the simultaneous modulation of photocycloaddition and photogenerated radicals in one system, realizes complete PL quenching and light-induced SMM behavior, providing a dynamical switch for the construction of multifunctional polymorphic materials with optical response and optical storage devices.

## Introduction

Light-switchable single-molecule magnets (SMMs), with slow relaxation of magnetization at a specific temperature and under light, have received considerable attention in recent years owing to their potential application in switches, sensors, and magnetic data storage devices at the molecular level [1–4]. Tremendous efforts have been devoted to the synthesis and characterization of light-switchable SMMs, especially using transition-metal complexes with light-induced spin transition [5–10]. In particular, lanthanide-based SMMs (Ln-SMMs) with light-switchable properties have recently attracted increasing attention as switchable bistable magnetic materials [11–14] due to their large magnetic moments, high single-ion magnetic anisotropy, and bistable ground states originating from their strong spin–orbit coupling and the crystal field effect of the Ln ions [15–19].

In general, modulating the light-switchable SMM behavior at room temperature is essential for practical applications. To obtain Ln-SMMs with tunable magnetic and optical properties induced by light, promoting structural changes by introducing photoresponsive organic ligands has emerged as a promising approach [13,20–23]. For example, the use of diarylethene and anthracene derivatives as ligands can enable controlled reversible SMM behavior via photoisomerization [24,25]. Anthracene and its derivatives are typically used for this purpose because they undergo light-induced [4 + 4] cycloaddition reactions, which render them good candidates for reversibly photoresponsive smart materials exhibiting photoluminescence (PL) [26,27], photochromism, and photomagnetism [28,29]. However, the cycloaddition reactions proceed only when the π–π stacking of the anthracene groups adopts a face-to-face arrangement, which is particularly challenging to achieve in polynuclear complexes. Furthermore, most cycloaddition reactions can only induce slight variations in the effective energy barrier and magnetic relaxation time; therefore, switching the SMM behavior remains a formidable task. Meanwhile, photomagnetic phenomena, including light-switchable SMM behavior, have been achieved in electron-transfer photochromic complexes in which photogenerated radicals tune the electrostatic ligand field, couple with paramagnetic metal ions, and change the magnetic behavior [30–32]. In this context, numerous photomagnetic compounds with tunable magnetic properties have been reported [33–36]. However, due to the weak magnetic coupling between photogenerated radicals and metal ions, the light-induced tunable SMM behavior remains restricted to a few examples [30–32].

Upon room-temperature irradiation, electron-transfer photochromic complexes and anthracycline derivatives quickly generate stable radicals and undergo structural changes due to photocycloaddition [37,38], respectively, thereby showing magnetic properties that are changed to a certain degree. Therefore, simultaneously, incorporating photocycloaddition and photogenerated radicals into one system is expected to substantially affect the electrostatic ligand field and magnetic coupling of Ln ions, enabling the switching of the SMM behavior in a single-component complex. However, utilizing the synergism of photogenerated radicals and cycloaddition to achieve switchable SMMs is still confronted with huge challenges and has not been realized for now. Recently, we confirmed that 9-anthracenecarboxylic acid (HAC) and its complexes can produce stable radicals upon irradiation, showing enhanced photochromic and photomagnetic properties [39]. Therefore, when HAC is used as a ligand with anisotropic Ln ions and the anthracene groups adopt a face-to-face π–π stacking, photogenerated radicals and photocycloaddition can be synchronously coupled in one complex upon Xe lamp irradiation, resulting in an obvious alteration in the exchange interaction between 4f ions and the concomitant SMM behavior.

Furthermore, photocontrollable luminescent Ln-SMMs are attractive not only because light can be conveniently and precisely controlled but also owing to the changes in their optical properties [40–42]. However, the synergistic modulation of the PL and magnetism of Ln-SMMs via irradiation is extremely difficult. In anthracene-containing Ln complexes, the electron-transfer process and photocycloaddition reactions give rise to remarkable structural changes, leading to drastic variations in the physical properties of the materials. Considering that both the PL and magnetic behavior can be substantially altered via structural changes originating from photochemical reactions, Ln complexes containing photoresponsive anthracene components are promising candidates to realize light-induced tunable optical and SMM bifunctions.

In this work, a series of cubane structures [Ln_4_(HAC)_2_(H_2_O)(OH)_4_(AC)_7_(NO_3_)]·3H_2_O (Ln = Dy for **1**, Gd for **2**, and Y for **3**) were synthesized via the self-assembly of HAC and Ln ions under hydrothermal conditions. Upon irradiation with a Xe lamp at room temperature, all structures exhibited remarkable photochromic phenomena, as confirmed by ultraviolet-visible (UV-vis), infrared radiation (IR), and electron spin resonance (ESR) spectra as well as single-crystal structure analyses. Concomitantly, complete PL quenching was observed in all structures due to the synergistic effect of photogenerated radicals and [4 + 4] photocycloaddition. Furthermore, a reversible color change was unexpectedly achieved by subjecting the compounds to mechanical grinding due to the quenching of photogenerated radicals. Importantly, the photocycloaddition of AC ligands and photogenerated stable radicals accelerated the change in the electrostatic ligand field and magnetic coupling and triggered the SMM behavior for the Dy analog upon Xe lamp irradiation. Thus, light-induced PL quenching and SMM behavior are realized for the first time by simultaneously regulating photocycloaddition and light-induced electron transfer in one system, providing a new approach for preparing materials with optical response and optical storage devices.

## Results and Discussion

**Fig. 1.**
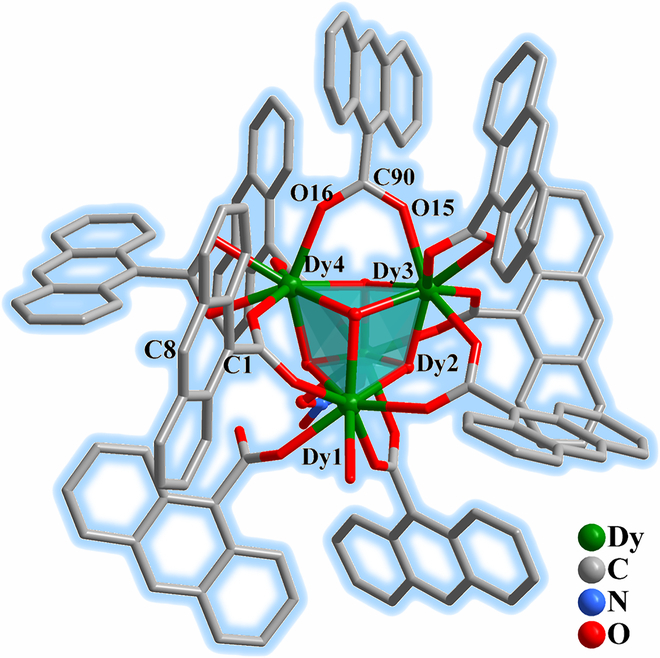
Molecular structure of 1. Lattice water molecules and H atoms are omitted for clarity. Color code: Dy, green; C, gray 40%; O, red; N, light blue.

Single-crystal x-ray diffraction (XRD) results revealed that all the compounds crystallized in the monoclinic *P*2_1_/*c* space group (Tables S1 and S2). The presence of lattice water molecules and the thermal stability of the compounds were confirmed via thermogravimetric (TG) analyses (Figs. S1 to S3). The phase purities were checked by powder x-ray diffraction (PXRD; Figs. S4 to S6) and elemental analysis. Owing to the similarity of the crystal structures, only that of **1** is discussed here. As shown in Fig. 1, the molecular skeleton of **1** contains a cubane [Dy_4_(*μ*_3_-OH)_4_] structure with 4 Dy^III^ centers, 4 OH^−^ groups, 9 AC units, one NO_3_^−^ groups, and one coordinated water molecule. In the tetranuclear structure, 4 Dy^III^ centers and 4 *μ*_3_-OH^−^ moieties occupied the vertices of the cubane core. All Dy atoms formed an 8-coordinated [DyO_8_] polyhedral coordination geometry. The coordination sites of a Dy atom were occupied by one water molecule, one monodentated AC ligand, 3 bridged AC ligands, and 3 OH^−^ groups. Another Dy atom was surrounded by one chelating NO_3_^−^ group, 3 bridged AC ligands, and 3 OH^−^ groups. The remaining 2 Dy atoms were coordinated by 3 bridged and one chelating AC ligands and 3 OH^−^ groups. The coordination configuration of the Dy^III^ ions was confirmed to be a triangular dodecahedron via continuous shape measurements (Table S3). The Dy–O bond lengths were in the range of 2.290(5) to 2.475(6) Å, and the O–Dy–O angles were in the range of 53.80(18) to 147.66(19)° (Table S10). The Dy···Dy distances within the cubane structure were similar within a range of 3.7108(8) to 3.8326(7) Å, while the shortest intermolecular Dy···Dy distance was 11.962(1) Å. An anthracene ring in the cubane structure was oriented almost face-to-face to an anthracene ring of the adjacent tetranuclear structure, exhibiting π···π interactions with a centroid-to-centroid distance of 3.6809(2) Å. For **2** and **3**, the Gd–O and Y–O bond lengths were in the range of 2.318(5) to 2.499(6) and 2.267(2) to 2.465(3) Å (Tables S12 to S16), and π···π interactions were also formed between the adjacent anthracene rings with a centroid-to-centroid distance of 3.6724(1) and 3.7581(0) Å for **2** and **3**, respectively (Figs. S7 and S8). Owing to the π···π interactions between the anthracene rings, all the compounds were prone to undergo [4 + 4] photocycloaddition reactions, which enable solid-state switchable photochromism, PL, and photomagnetism [43,44].

**Fig. 2.**
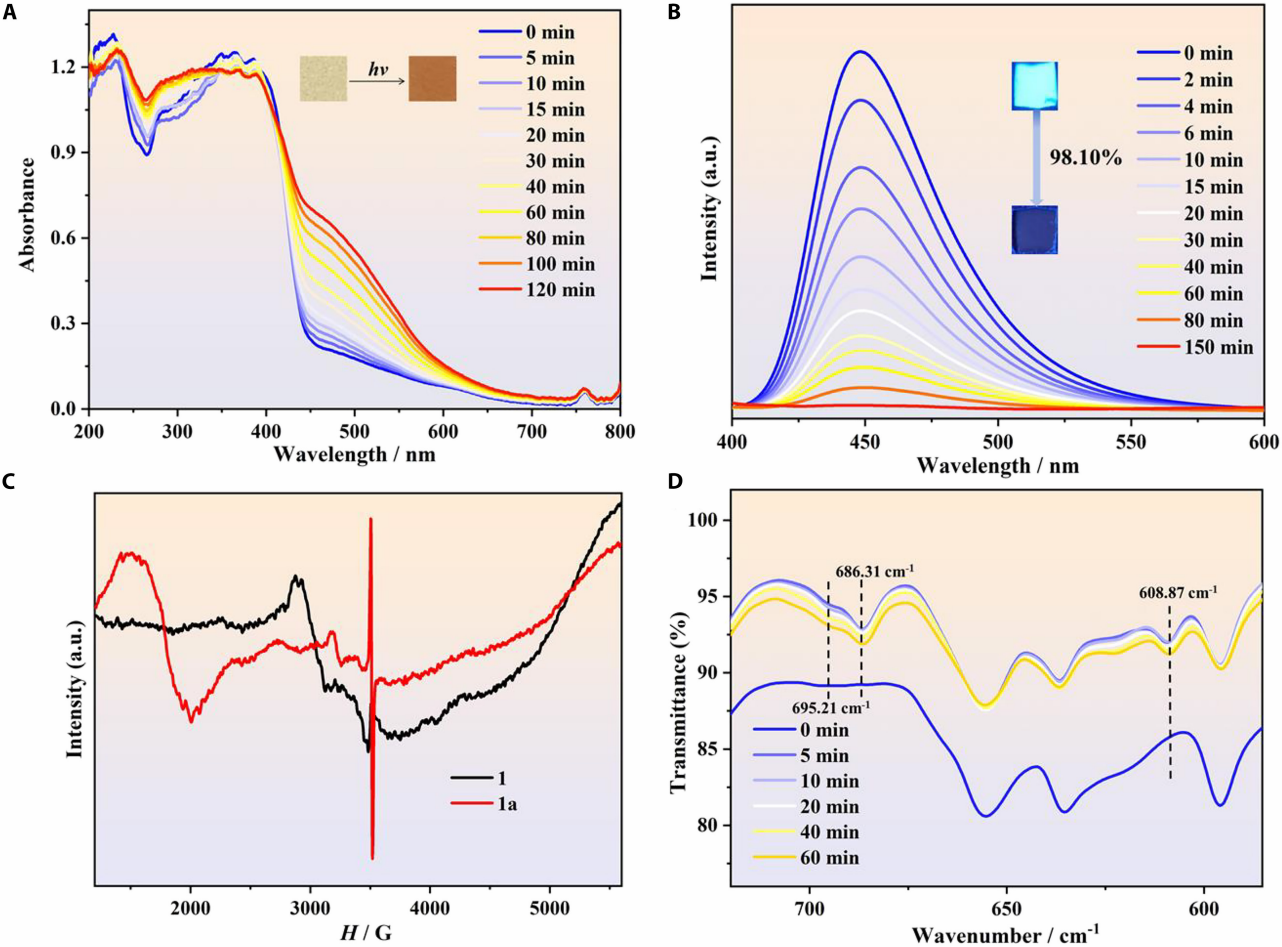
(A) Time-dependent UV-vis spectra of a powder sample of 1 in the solid state upon light irradiation. Inset: Photos of apparent color upon irradiation. (B) In situ PL spectra of 1 after excitation at 360 nm. Inset: Photos of luminous color before and after light irradiation. (C) Room-temperature ESR spectra of 1 and 1a at a frequency of 9.84 GHz. (D) Time-dependent IR spectra of 1 at room temperature.

The photochromic electron-transfer and [4 + 4] photocycloaddition properties of the AC ligand prompted us to explore the photoresponsive behavior of this series of compounds under ambient conditions. For **1**, the light yellow color of the powder sample intensified deeper upon Xe lamp (320 to 780 nm; 300 W) irradiation and progressively became brown after 60 min of irradiation (inset of Fig. 2A and Fig. S9). The colored samples are hereinafter referred to as **1a**. The UV-vis spectra of **1** in the solid state were obtained to verify this light-induced phenomenon. As shown in Fig. 2A, a new and broad absorption band centered at 530 nm appeared and gradually intensified with increasing irradiation time of the Xe lamp, indicating the formation of stable radical species, aligning with most existing reports on the electron-transfer photochromic materials [30]. Furthermore, the intensity of the peaks at ~380 nm decreased upon light irradiation, whereas that of the peaks at ~260 nm increased, indicating the formation of dimer structure via photocycloaddition [23,45]. Because the photocycloaddition reaction is detected below 400 nm in the UV-vis spectra, the photochromic behavior is mainly attributed to the formation of stable radicals triggered by photonic stimuli. The room-temperature in situ PL behavior of **1** was subsequently studied in the solid state. The PL spectra of **1** showed a maximum emission at 445 nm when the samples were excited at 360 nm, which can be attributed to intraligand π–π* or n–π* transitions in the AC components [46]. Compared with the initial state, the intensity of the fluorescence peak drastically decreased by 98.10% after irradiation using a 360-nm laser for 150 min (Fig. 2B). Furthermore, the pristine light yellow crystalline powder of **1** emitted blue fluorescence when excited at 360 nm. After irradiation, the luminous color disappeared with the apparent color changed to brown, which was consistent with the obtained PL spectra (inset of Fig. 2B). The Gd and Y analogs exhibited similar photochromic and PL behaviors (Figs. S10 to S15), with the intensity of the fluorescence peak drastically decreasing by 99.72% and 99.67%, respectively. Thus, the PL quenching showed the largest intensity decrease in the electron-transfer photochromic materials. This rare phenomenon involving complete PL quenching of photochromic materials stems from the synergic effect of the light-induced electron transfer and cycloaddition of the AC components. The drastic intensity decreases in the PL spectra indicate the occurrence of structural changes after irradiation.

To explore the photochromic and photoluminescent phenomena, the solid-state ESR spectra of all the compounds were obtained before and after irradiation. As shown in Fig. 2C, multiple resonances belonging to Dy^III^ ions were observed for the as-prepared samples. After irradiation, a sharp and strong peak at *g* = 2.008 appeared, confirming the photogeneration of radicals in **1a**. Moreover, a change occurred in the Dy^III^ signals after irradiation, most likely resulting from the generation of radicals or structural changes. A similar result was observed in the ESR spectra of the Y^III^ analog **3**, which showed a sharp radical signal at *g* = 2.003 (Fig. S16). For **2**, due to the half-filled electronic configuration *f*^7^ of the Gd^III^ centers, a broad resonance peak at *g* = 2.004 appeared in the ESR spectra (Fig. S17), while the characteristic radical signal was not observed after irradiation probably because it was obscured by the strong Gd^III^ signal [47]. However, the intensity of the metal signal changed after irradiation, suggesting the generation of radicals or the occurrence of structural changes. Furthermore, IR spectra were obtained to explore chemical reactions occurring during the photochromic process. As shown in Fig. 2D and Fig. S18, new peaks at 609, 686, and 695 cm^−1^ appeared in the spectra of **1** after irradiation, which can be attributed to the C–H out-of-plane deformation vibration of dianthracenes caused by photocycloaddition [48]. The Gd and Y analogs showed similar IR spectral changes, suggesting the occurrence of a photocycloaddition reaction after irradiation (Figs. S19 and S20). These results indicate that both the photocycloaddition reaction and radicals were generated after irradiation for all the compounds.

**Fig. 3.**
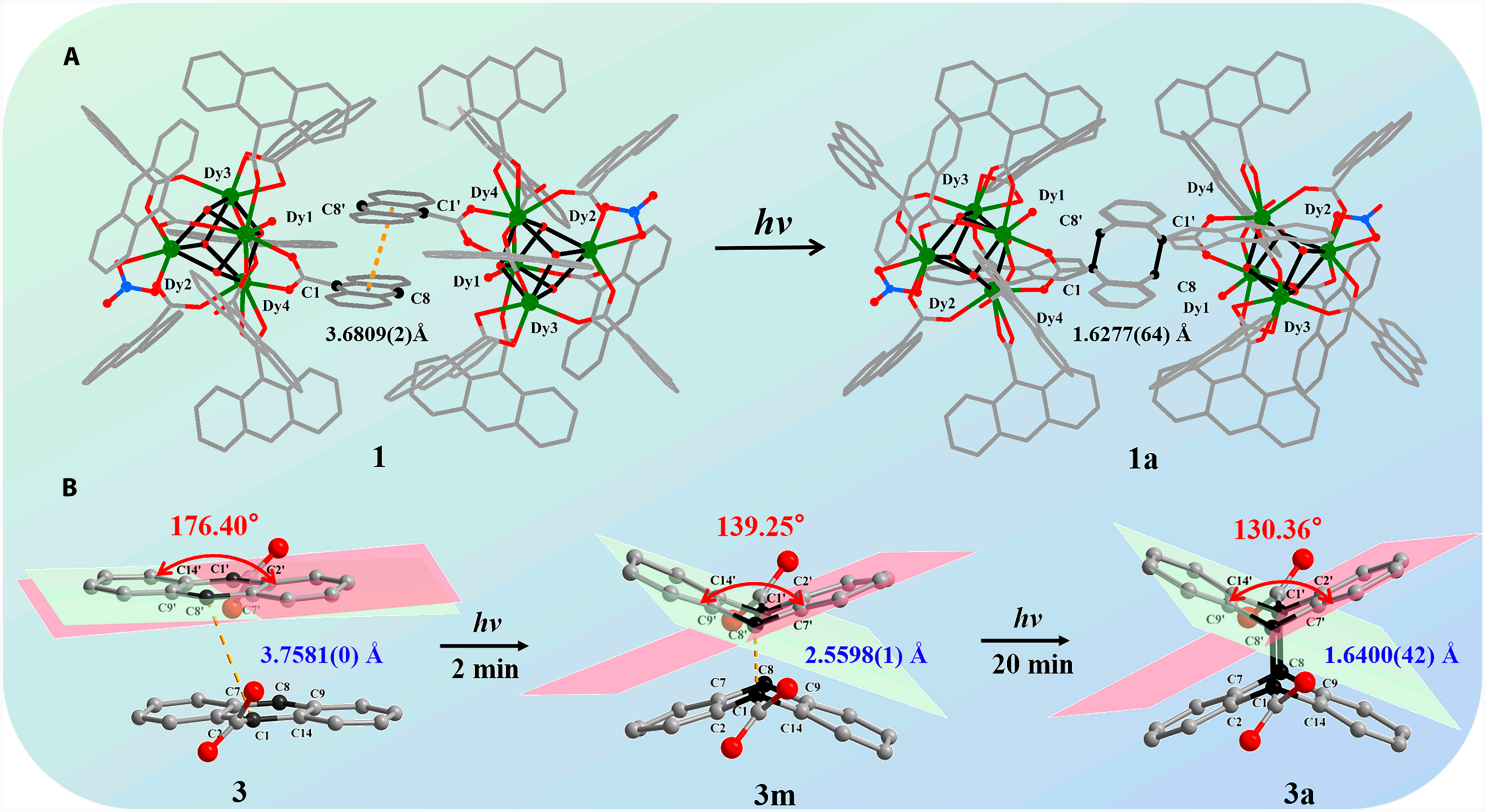
(A) Crystal structures of 1 (left) and 1a (right) exhibiting the anthracene stacking in 1 before and after photocycloaddition to 1a. (B) Changes in the adjacent anthracene components of 3 during light irradiation.

To further confirm the photoresponsive behavior, the crystal structures of the compounds were analyzed in detail. As shown in Fig. 3A, the C1···C8′ distance between the peripheral anthracene moieties in **1** was 3.6809(2) Å (<4.2 Å), suggesting that a solid-state photocycloaddition reaction could occur during the color-change process [49,50]. In fact, dimerization appeared via photocycloaddition on the central benzene rings of adjacent anthracenes (C1–C8′ and C1′–C8 = 1.6277(64) Å) after Xe lamp irradiation. Furthermore, the C1–C8′ distance was shorter than that of reported dianthracene derivatives (1.66 Å) [51], indicating fully completed photocycloaddition process. Photocycloaddition also occurred in the Gd and Y analogs, with the distance between photocycloaddition rings being 1.6351(91) and 1.6400(42) Å for **2** and **3**, respectively (Figs. S7 and S8). In addition to the obvious variations in the cycloaddition components, other AC components in the 3 structures showed detectable changes before and after irradiation. The structure of **1** is discussed here as a representative example. The anthracene carboxylate group containing the C90 atom also showed considerable changes after irradiation. In this carboxylate moiety, the C90–O15 and C90–O16 bond lengths changed from 1.2611(116) and 1.3036(111) to 1.2484(64) and 1.2597(67) Å, respectively. Concomitantly, remarkable changes were observed for the middle benzene ring after irradiation, with C77–C82, C82–C83, C76–C77, and C84–C89 bond lengths changing from 1.3890(106), 1.3810(79), 1.4499(128), and 1.3905(106) to 1.4630(99), 1.4052(108), 1.3948(81), and 1.4464(88) Å, respectively. As a result, the structure showed electron transfer from the carboxylate group to the anthracene ring after irradiation, leading to the generation of free radicals and the occurrence of photochromism [36,39]. Based on the changes in the bond lengths, we propose a schematic (Fig. S21) for the structural changes after irradiation.

Surprisingly, an intermediate state was detected in the [4 + 4] photocycloaddition reaction of the Y analog. Upon irradiating the crystals of **3** for 2 min, the dimerization of neighboring anthracene rings began, forming an intermediate state **3m** (Fig. 3B), in which one of the AC ligands was bent in the middle benzene ring and moved closer upon irradiation, with the dihedral angle between the plane (C1, C2, C7, and C8) and the plane (C8, C9, C14, and C1) decreasing from 176.40° to 139.25° and C1–C8′ distance decreasing from 3.7581(0) to 2.5598(1) Å. Upon further irradiation, the C1–C8' distance decreased to 1.6400(42) Å, while the dihedral angle slightly decreased to 130.36°. As a result, the adjacent molecules moved closer as the photodimerized AC ligands changed from the slip-stacked manner to face-to-face mode, while the anthracene rings first bent and then shifted closer to form C–C bonds.

**Fig. 4.**
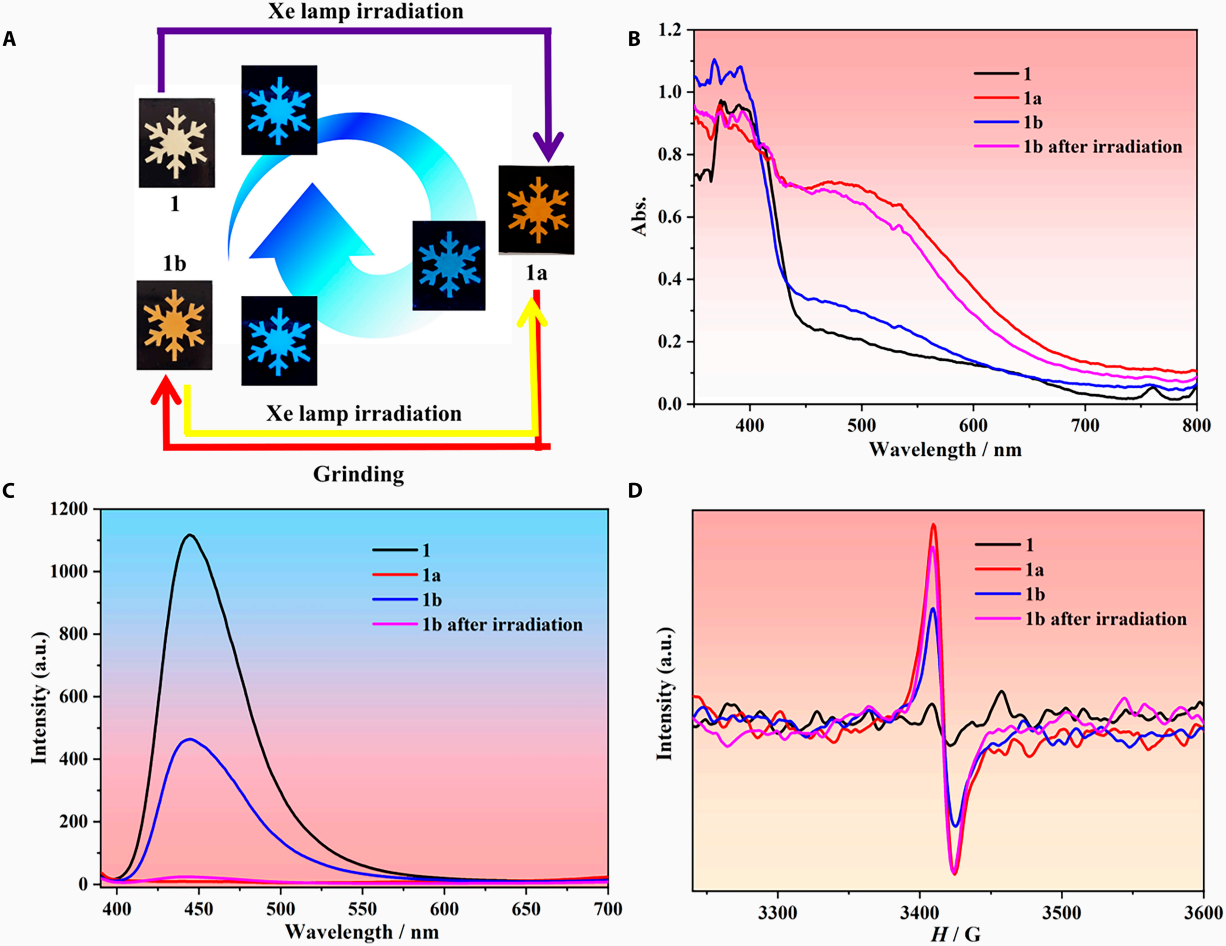
(A) Reversible appearance and luminous color of 1, 1a, and 1b. (B) UV-vis spectra of 1, 1a, 1b, and 1b after irradiation. (C) PL spectra of 1, 1a, 1b, and 1b after irradiation upon excitation at 360 nm. (D) ESR spectra of 1, 1a, 1b, and 1b after irradiation between 3,220 and 3,600 G field at 296 K.

In general, photogenerated radicals and ligand photocycloaddition products can be relaxed to the initial state via thermal treatment or irradiation or by keeping in the dark for days [52,53]. Due to the large π-conjugated anthracene components [39,54] as well as strong crystal rigidity and steric hindrance effects between the molecules [29,41], the light-induced phenomena simultaneously caused by the photogeneration of radicals and photocycloaddition in the developed compounds were extremely stable under ambient conditions and could not be reversed even by irradiation or heating. However, the compounds showed reversed discoloration behavior induced by mechanical grinding. For **1**, a reversible color change was detected with naked eyes after grinding the colored powder **1a** with a pestle (the ground samples are hereinafter referred to as **1b**; Fig. 4A and Fig. S22). In particular, the quenched fluorescence returned to the origin state (blue emission) after grinding. Samples **1b** were subjected to UV-vis, fluorescence, ESR, and IR spectra measurements after irradiation. As shown in Fig. 4B and C, the intensity of the photogenerated absorption peak substantially decreased, with the emission partially relaxing to the initial state. Furthermore, the ESR spectra of **1b** showed a drastic weakening in the intensity of the radical peak (Fig. 4D), whereas the peaks ascribed to the C–H out-of-plane vibration of dianthracenes remained almost unchanged after grinding (Fig. S18), indicating that radical quenching was the main reason for the reversible coloration behavior. Furthermore, the mutable transition between **1a** and **1b** was achieved by alternating irradiation and grinding process, as confirmed by UV-vis, PL, and ESR spectra (Fig. 4). A similarly reversible coloration behavior was observed in the Gd and Y analogs (Figs. S23 to S28). These unexpected results indicated that the reversible color-change property of these compounds could be activated on by irradiation and partially deactivated by mechanical grinding. In the as-prepared crystal structures, the tetranuclear molecules were orderly arranged with face-to-face π···π interactions as well as C–H···O and C–H···π bonding interactions between them. After irradiation, the photogeneration of radicals mainly elicited the photochromic behavior. In **1**, the C–H···O bond lengths were 3.184(5) and 2.5098(54) Å and the C–H···π bond lengths were 2.5875(2) Å (Fig. 29). These weak intermolecular noncovalent interactions between anthracene rings effectively stabilized the delocalized radicals in the anthracene rings. Upon grinding, these intermolecular interactions became weak, which increased the contact between free radicals and O_2_, leading to radical quenching and partially reversible coloration process in these compounds. In addition, grinding did not destroy the crystal structures (Figs. S4 to S6), which allowed excluding the loss of crystallinity as the reason for the discoloration [55,56]. Thus, the dynamically switchable photochromism and PL render the compounds promising as optical storage materials [57].

**Fig. 5.**
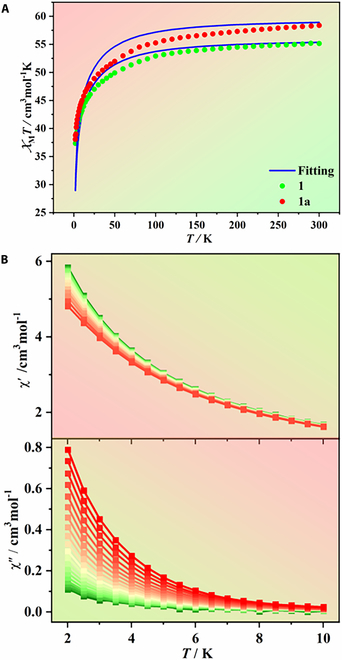
(A) Temperature-dependent magnetic susceptibilities of 1 and 1a under a dc magnetic field of 1,000 Oe. Calculated (blue solid line) data of magnetic susceptibilities of 1 and 1a. The intermolecular interactions *zJ*′ of 1 and 1a were fitted to −0.02 and 0.02 cm^−1^, respectively. (B) Temperature dependence of the in-phase and out-of-phase components of ac magnetic susceptibilities for 1a in a 1,200-Oe dc field at various ac frequencies.

The photogeneration of radicals and photocycloaddition in the structures probably caused the photomagnetic behavior for these compounds. Therefore, the magnetic susceptibilities of **1** before and after irradiation were first measured under a direct current (dc) magnetic field of 1,000 Oe between 2 and 300 K (Fig. 5A). The *χT* value, where *T* is the temperature in kelvin and *χ* is the molar magnetic susceptibility, was 55.15 cm^3^ mol^−1^ K at 300 K, slightly lower than the expected value for 4 Dy^III^ centers (56.68 cm^3^ mol^−1^ K). Upon cooling, the *χT* value gradually decreased to 37.35 cm^3^ mol^−1^ K at 2 K, which can be attributed to the depopulation of the Stark levels of the Dy^III^ center with crystal field splitting [58]. After coloration, the *χT* value increased to 58.37 cm^3^ mol^−1^ K at 300 K, and larger than the as-prepared samples in the entire temperature range. This result indicated that the photogeneration of radicals and photocycloaddition induced the observed changes in the magnetic coupling of the cubane structure. To further explore the magnetic coupling between the photogenerated radicals and Dy^III^ ions, the temperature-dependent magnetic susceptibilities of **2** and **3** were measured under the same conditions. The *χT*–*T* curve of the Y^III^ analog exhibited a linear decrease (Fig. S30), while the Gd^III^ analog and **1** showed similar *χT*–*T* curves (Fig. S31). The magnetic-field (*H*) dependence of magnetization (*M*) was evaluated before and after irradiation at 2 K. The *M* values of **1** at 50 kOe gradually reached 21.13 Nβ before irradiation. After irradiation, the *M* value gradually increased to 22. 39 Nβ at 50 kOe (Fig. S32). For the Gd^III^ analog, the *M* values at 50 kOe reached 25.09 and 26.48 Nβ before and after irradiation, respectively (Fig. S33). The results confirmed that the photomagnetic behaviors originated from variations in the ligand fields and magnetic couplings of Dy^III^ ions due to light-induced electron transfer and structure changes.

To comprehensively explore the effect of the photogenerated radicals and photocycloaddition on the dynamic magnetic properties of **1**, the magnetic susceptibilities were measured under alternating current (ac) magnetic field of 5 Oe ac field. Before irradiation, no frequency-dependent behavior was observed in the temperature-dependent magnetic susceptibilities (Fig. S34). However, both the in-phase (*χ’*) and out-of-phase (*χ”*) components of **1a** exhibited frequency dependence under a zero dc and 5 Oe ac magnetic field (Fig. S35), showing slow magnetic relaxation. Due to fast quantum tunneling of the magnetization (QTM), no peaks for the *χ’* and *χ”* components were observed in the measured temperature range. To suppress the QTM process, the optimum dc magnetic field was determined by performing variable-field ac measurements between 0 and 5,000 Oe at 2 K (Fig. S36) and used in the external field-influenced ac magnetic susceptibility measurements. As shown in Fig. 5B and Figs. S37 and S38, under the optimum dc magnetic field of 1,200 Oe, the *χ’* and *χ”* components decreased while the corresponding peaks were still not observed. Due to the fast QTM, the fitting result of the relaxation energy barrier failed. However, this relaxation phenomenon was similar with other compounds with relaxation dynamics, thus confirming the SMM behavior of the compounds prepared in this work [59].

**Fig. 6.**
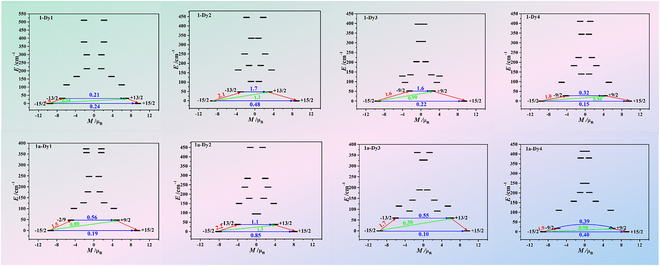
Magnetization blocking barriers of individual Dy^III^ fragments for 1 and 1a. The thick black lines represent the KDs of individual Dy^III^ fragments as a function of their magnetic moment along the magnetic axis. The blue lines correspond to the diagonal matrix element of the transversal magnetic moment; the green lines represent Orbach relaxation processes. The path indicated by the red arrows represents the most probable path for magnetic relaxation in the corresponding compounds. The numbers at each arrow stand for the mean absolute value of the corresponding matrix element of transition magnetic moment.

To gain insights into the magneto-structural relationship of the compounds, complete-active-space self-consistent field (CASSCF) calculations were performed on 8 individual Dy^III^ fragments, namely, **1**-**Dy1**, **1**-**Dy2**, **1**-**Dy3**, **1**-**Dy4**, **1a**-**Dy1**, **1a**-**Dy2**, **1a**-**Dy3**, and **1a**-**Dy4** (Fig. S39), based on the x-ray-determined geometries using the OpenMolcas [60] and SINGLE_ANISO [61–63] programs (see the “computational details”). The energy levels (cm^−1^), *g* (*g*_x_, *g*_y_, and *g*_z_) tensors, and predominant *m_J_* values of the 8 lowest Kramers doublets (KDs) are shown in Table S17. All the *m_J_* values were found to be ±15/2 in their ground KDs with *g*_x,y_ ≈ 0.000 and *g*_z_ ≈ 20.000, indicating a nearly perfect axial anisotropy. The mixed *m_J_* components for the 2 lowest KDs of the individual Dy^III^ fragments in **1** and **1a** are shown in Table S18. KD_0_ in **1** and **1a** are almost composed of *m_J_* = ±15/2, while KD_1_ comprises several *m_J_*. As depicted in Fig. 6, the transversal magnetic moments in the ground KDs are all larger than 10^−1^ *μ*_B_ in the magnetization blocking barriers of the 8 individual Dy^III^ fragments, thus allowing a fast QTM in their KD_0_ level. Thus, the energy barriers of **1** and **1a** must be zero according to the calculated results of the 8 individual Dy^III^ fragments.

However, the weak Dy^III^–Dy^III^ and Dy^III^–radical interactions may exert a nonnegligible influence on their slow magnetic relaxation processes. For **1** and **1a**, the calculated ground *g*_z_ values of the 8 individual Dy^III^ fragments were all close to 20.000; thus, the Dy^III^–Dy^III^ and Dy^III^–radical interactions can be regarded to belong to the Ising type. The POLY_ANISO program [61–62] was used to fit the Lines [64] exchange coupling constants (*J*; effective coupling constant ${\tilde{J}}_{1}=25J_{1}\cos\theta \;$for the Dy^III^–Dy^III^ interactions and ${\tilde{J}}_{2}=5J_{2}\;$ for the Dy^III^–radical interactions) and the intermolecular interaction parameter (*zJ′*) by comparing the computed and measured magnetic susceptibilities for **1** and **1a**. During the fitting, the Dy^III^–Dy^III^ interactions were simply considered equal due to similar coordination environments and proximity between the Dy^III^ centers (Fig. S40).

The parameters ${\tilde{J}}_{1}\;$and ${\tilde{J}}_{2}$ were calculated with respect to the pseudospin $\tilde{S}$ = 1/2 for the Dy^III^ ion. The calculated plots of *χT* versus *T* for **1** and **1a** are displayed in Fig. 5A, which show a good agreement between the calculated and experimental curves. The fitted negative values of $\tilde{J}$ for **1** (${\tilde{J}}_{1}$ = −0.75 cm^−1^) and **1a** (${\tilde{J}}_{1}$ = −0.90 cm^−1^ and ${\tilde{J}}_{2}$ = −1.00 cm^−1^) indicate that the total interactions between Dy^III^–Dy^III^ and Dy^III^–radical are antiferromagnetic. Moreover, the radical–radical interaction in **1a** is very weak due to the large distance between them (Fig. S21); thus, it was not fitted. The exchange energies (*E*; cm^−1^), energy difference between each exchange doublet (Δ*_t_*; cm^−1^), and predominant *g_z_* values for the lowest 8 and 24 exchange doublets arising from the exchange interactions on the magnetic centers **1** and **1a** are shown in Table S19. The *g*_z_ values of the ground exchange states of **1** and **1a** were 11.352 and 11.712, respectively, confirming that the interactions in **1** and **1a** are antiferromagnetic. According to the exchange energy levels shown in Table S19, the energy barrier of **1a** cannot be associated with the exchange states because the highest of these states (2.9 cm^−1^; Table S19) lies lower than the value of the extracted barrier (8.4 cm^−1^), indicating that this regime must be related to relaxation through the excited KDs of individual metal ions. The calculated orientations of the local main magnetic axes on the Dy^III^ ions of **1** and **1a** in the ground state are depicted in Fig. S41, which shows that the magnetic axes have an arbitrary discrete distribution.

**Fig. 7.**
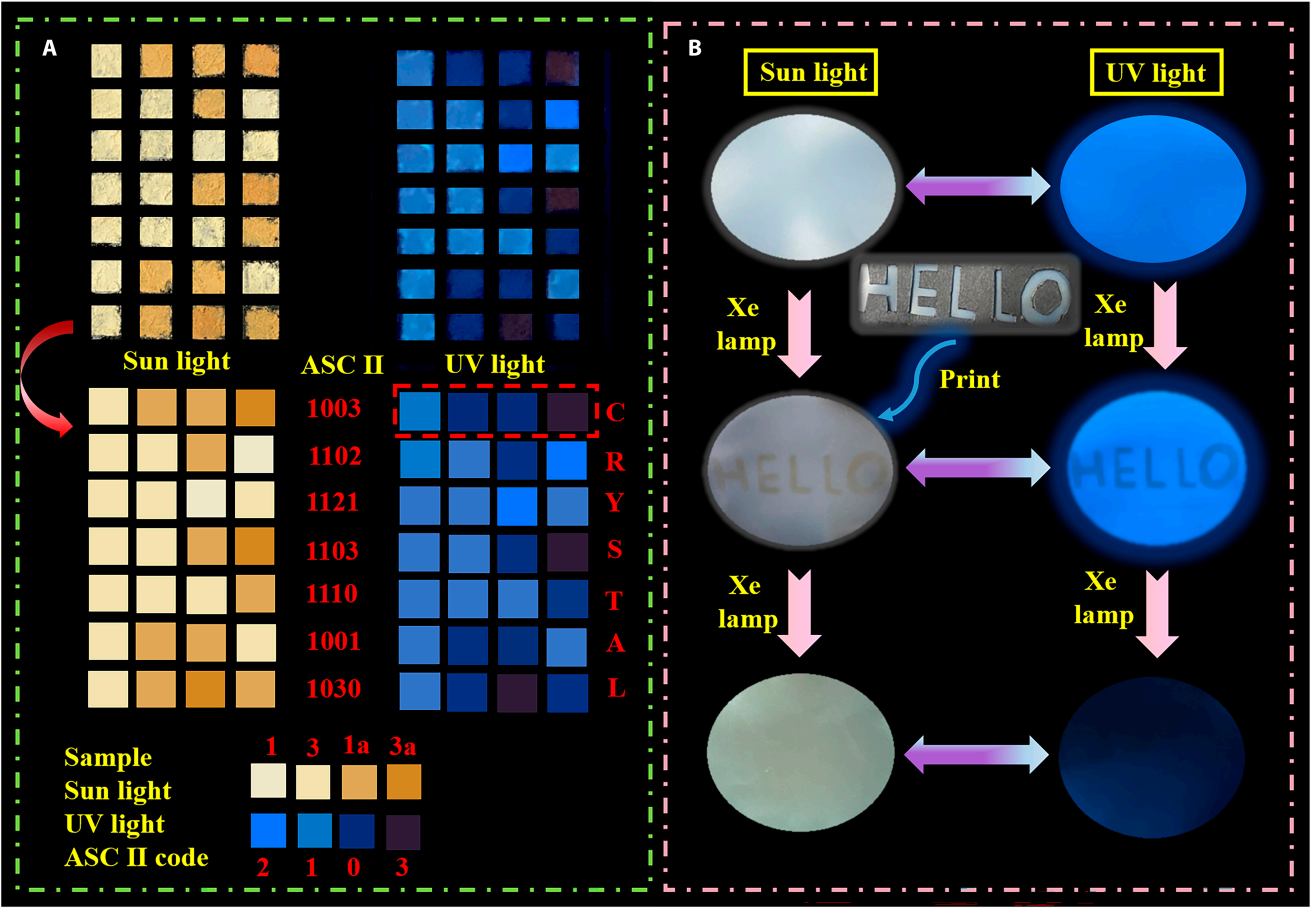
(A) Schematic diagram of encrypted information based on the photochromic and photoluminescent properties of compounds 1 and 3. (B) Schematic diagram of the light-mediated inkless printing based on compound 1.

Derived from the remarkably photochromic and PL properties, these compounds show great potential in the applications of information storage and encryption. Therefore, an anti-counterfeit encryption system was firstly designed according to the changes of photochromism and PL by ASCII encryption algorithm (American Standard Code for Information Exchange). As shown in Fig. 7A, owing to the distinction of apparent and luminous color between compounds **1** and **3,** the samples were defined as different information codes in the note and uniformly placed in a square matrix of 4 × 7. Under the irradiation with a 365-nm UV light, the samples of **1** and **3** in the square matrix exhibited blue emission and output information was defined as “2” and “1.” Samples **1a** and **3a** without emission was “0” and “3” in the output information. Based on the changes under UV light, each line code was interpreted as a different digital code, converting ASCII algorithm to obtain effective information “CRYSTAL.” In addition, a writable material with compound **1** was successfully developed utilizing inkless printing technology. The fabricated film still showed excellent luminescence characteristics under UV light, indicating effective integration with the polymer. The mask with hollow-out “HELLO” letters was covered on the film and illuminated by Xe lamp for 30 min at room temperature. Originated from the electron-transfer photochromic behavior, the “HELLO” word was then printed on the film after removing the mask. The printed word became more obvious under UV light but further disappeared after persistent Xe lamp light irradiation. Therefore, the synthesized compounds can enhance the application potential in information anti-counterfeiting and inkless printing.

### Conclusion

Here, a series of tetranuclear cubane complexes with switchable optical and magnetic properties were synthesized. The cubane complexes underwent light-induced dual structural changes, involving the photogeneration of radicals and [4 + 4] photocycloaddition, which elicited photochromic behavior and complete PL quenching. The quenched PL showed the largest fluorescence intensity change (99.72%) observed in electron-transfer photochromic materials. A reversible decoloration process unexpectedly occurred upon mechanical grinding, which stemmed from partial radical quenching, as confirmed by UV-vis, PL, IR, and ESR spectra. This study provides a novel approach to realize reversible properties of electron-transfer photochromic compounds triggered by mechanical force. Importantly, the photocycloaddition of AC ligands and photogenerated stable radicals unexpectedly changed the electrostatic ligand field and magnetic coupling, triggering the SMM behavior for the Dy analog after Xe lamp irradiation. The ab initio calculations of magnetization relaxations were performed to confirm the light-induced SMM behavior. Furthermore, these compounds were successfully applied in information anti-counterfeiting and inkless printing. This work for the first time achieved the totally quenched luminescence and SMM behavior via the synergy of dual photoactive response originated from photogenerated radicals and cycloaddition, providing an innovative strategy for the development of multifunctional polymorphic materials with optical response and optical storage devices.

## Materials and Methods

### Materials and syntheses

All chemicals were reagent grade and used as purchased without further purification.

#### Synthesis of **1**

A mixture of Dy(NO_3_)_3_·6H_2_O (0.2 mmol, 0.09 g) and 9-anthracenecarboxylic acid (HAC; 0.45 mmol, 0.10 g) was dissolved in 5 ml of CH_3_OH and 5 ml of CH_3_CN. The resultant solution with 0.20 ml CH_3_NH_2_ was added in a Teflon-lined autoclave (20 ml) and placed in an oven at 120 °C to react for 5 days. Orange x-ray-quality crystals were obtained and dried in air. Yield: ca. 64% based on Dy(NO_3_)_3_·6H_2_O. Compound **1a** was obtained by irradiating **1** with a Xe lamp at room temperature for 150 min: Elemental analysis for compound **1** (%): calcd for C_135_H_95_NO_29_Dy_4_ (2,845.11): C, 56.99; H, 3.37; N, 0.49. Found: C, 56.68; H, 3.31; N, 0.52. IR (KBr pellet, cm^−1^): 3,417(m), 3,043(w), 2,919(w), 2,852(w), 1,660(w), 1,575(s), 1,490(m), 1,434(s), 1,386(s), 1,315(s), 1,272(m), 1,012(w), 952(w), 889(m), 854(w), 786(w), 738(s), 651(m), 595(w), 563(w), 526(w). For **1a** (%): calcd for C_135_H_95_NO_29_Dy_4_ (2,845.11): C, 56.99; H, 3.37; N, 0.49. Found: C, 57.19; H, 3.16; N, 0.55. IR (KBr pellet, cm^−1^): 3,423(w), 3,054(w), 2,921(w), 1,660(w), 1,575(s), 1,486(w), 1,436(s), 1,386(s), 1,319(s), 1,272(m), 1,020(w), 867(w), 775(w), 730(s), 684(w), 649(m), 595(s), 551(w), 522(w).

#### Synthesis of **2**

The crystals of **2** were prepared in a similar way with compound **1** by replacing Dy(NO_3_)_3_·6H_2_O with Gd(NO_3_)_3_·6H_2_O (0.09 g, 0.2 mmol). Yield: ca. 42% based on Gd(NO_3_)_3_·6H_2_O. Compound **2a** was obtained by irradiating **2** with a Xe lamp at room temperature for 150 min: Elemental analysis for compound **2** (%): calcd for C_135_H_95_NO_29_Gd_4_ (2,824.11): C, 57.41; H, 3.39; N, 0.50. Found: C, 57.52; H, 3.23; N, 0.41. IR (KBr pellet, cm^−1^): 3,430(m), 3,046(w), 2,925(m), 2,364(w), 1,660(w), 1,577(s), 1,490(w), 1,442(s), 1,386(s), 1,321(s), 1,274(m), 1,012(w), 881(w), 850(w), 730(m), 653(w), 595(w), 563(w), 518(w). For **2a** (%): calcd for C_135_H_95_NO_29_Gd_4_ (2,824.11): C, 57.41; H, 3.39; N, 0.50. Found: C, 57.24; H, 3.22; N, 0.47. IR (KBr pellet, cm^−1^): 3,426(w), 3,039(w), 2,923(w), 2,358(w), 1,666(w), 1,571(s), 1,490(w), 1,442(s), 1,384(s), 1,321(s), 1,276(m), 1,043(w), 875(w), 754(w), 730(m), 692(w), 649(w), 597(w), 558(w), 514(w).

#### Synthesis of **3**

Crystals of **3** were prepared in a similar way by using Y(NO_3_)_3_·6H_2_O (0.09 g, 0.2 mmol) instead of Dy(NO_3_)_3_·6H_2_O. Yield: ca. 48% based on Y(NO_3_)_3_·6H_2_O. Compound **3a** was obtained upon irradiation of **3** by the Xe lamp at room temperature for 150 min: Elemental analysis for compound **3** (%): calcd for C_135_H_95_NO_29_Y_4_ (2,550.75): C, 63.57; H, 3.75; N, 0.55. Found: C, 63.44; H, 3.61; N, 0.52. IR (KBr pellet, cm^−1^): 3,427(m), 3,043(w), 2,928(w), 2,355(w), 1,670(w), 1,582(s), 1,485(m), 1,436(s), 1,385(s), 1,317(s), 1,277(m), 1,170(w), 1,017(w), 950(w), 889(w), 787(w), 730(s), 646(m), 598(m), 558(w), 518(w). For **3a** (%): calcd for C_135_H_95_NO_29_Y_4_ (2,550.75): C, 63.57; H, 3.75; N, 0.55. Found: C, 63.36; H, 3.45; N, 0.61. IR (KBr pellet, cm^−1^): 3,423(m), 3,045(w), 2,928(w), 2,354(w), 1,670(w), 1,581(s), 1,483(m), 1,434(s), 1,387(s), 1,310(s), 1,276(m), 1,170(w), 1,015(w), 955(w), 889(w), 787(w), 733(s), 648(m), 598(m), 558(w), 520(w).

#### Preparation of inkless printing

Compound **1** (0.02 g) was blended with poly(butylene adipate-co-terephthalate) at a ratio of 1:100 and dissolved in CH_2_Cl_2_ (12 ml). The obtained solution was poured into a petri dish to form a film after CH_2_Cl_2_ slowly evaporated.

### Physical measurements

#### Elemental, TG, magnetic, and optical analyses

Elemental analyses (for C, H, and N) were performed on a Perkin-Elmer 240C analyzer (Perkin-Elmer, USA). TG analyses were performed under a N_2_ atmosphere on a Rigaku standard TG-DTA (differential thermal analysis) analyzer. IR spectra were obtained via a MAGNA-560 (Nicolet) FT-IR spectrometer with KBr pellets. Luminescence data were measured by a Hitachi F-7000 fluorescence spectrometer (150 W, 360-nm UV light). UV-vis spectra were analyzed by a Puxi Tu-1901 spectrophotometer (using BaSO_4_ as a reference). ESR spectra were carried out by a CIQTEK EPR200-Plus spectrometer at room temperature. Magnetic measurements of polycrystalline samples were performed on a Quantum Design SQUID magnetometer. PXRD patterns were recorded on a Rigaku diffractometer with a Cu-target tube and a graphite monochromator. Simulation of the PXRD curves was conducted by the single-crystal data and diffraction-crystal module of the mercury (Hg) program with free of charge on the internet at http://www.iucr.org. For irradiation experiments, a Perfect Light PLS-SXE 300 Xe lamp (320 to 780 nm, 300 W, used for at least 150 min) was equipped to prepare the colored samples for elemental analysis, PXRD, ESR, and magnetic studies.

#### X-ray crystallography

The single-crystal XRD data of **1**, **1a**, **2**, **2a**, **3**, **3m**, and **3a** were collected on a Rigaku XtaLAB MM007 CCD diffractometer with Mo-Kα radiation (*λ* = 0.71073 Å). The SHELX-2016 software was used to solve the structures. For all compounds, solvent molecules were largely disordered, and the diffraction data were treated by the “SQUEEZE” method. Detailed crystallographic data for **1**, **1a**, **2**, **2a**, **3**, **3m**, and **3a** were summarized in Tables S1 and S2, and the selected bond lengths and angles were listed in Tables S10 to S16. Full crystallographic data for all compounds have been deposited in the Cambridge Crystallographic Data Center (CCDC).

#### Computational details

Considering the large sizes of **1** and **1a**, all the ring ligands except for the radical in **1a** were simplified to C_6_H_5_ (Fig. S39). CASSCF calculations for 8 individual Dy^III^ fragments, namely, **1**-**Dy1**, **1**-**Dy2**, **1**-**Dy3**, **1**-**Dy4**, **1a**-**Dy1**, **1a**-**Dy2**, **1a**-**Dy3**, and **1a**-**Dy4** (Fig. S39), were performed using the OpenMolcas [61] program package based on the single-crystal x-ray-determined geometries. All the 8 individual Dy^III^ fragments were studied keeping the experimentally determined structures of the corresponding compounds while replacing the other 3 Dy^III^ ions with diamagnetic Lu^III^.

Atomic natural orbitals from the ANO-RCC library were used as the basis sets for all atoms, i.e., ANO-RCC-VTZP for Dy^III^ and the O close to it and ANO-dk3 for distant atoms. The second-order Douglas–Kroll–Hess Hamiltonian was employed for the calculations, where scalar relativistic contractions were considered in the basis set and spin–orbit couplings coupling was handled separately by following the restricted active space state interaction (RASSI-SO) procedure. For the 8 individual Dy^III^ fragments, 9 active electrons in 7 active orbitals included all *f* electrons [CAS (9 in 7)] in the CASSCF calculations. To exclude all the doubts, all the roots in the active space were calculated. Only the maximum number of spin-free states that were possible with our hardware were mixed (all 21 sextets, 128 from 224 quadruplets, and 130 from 490 doublets). The SINGLE_ANISO [61–62] program was used to obtain the energy levels, *g* tensors, *m_J_* values, and magnetic axes based on the above CASSCF/RASSI-SO calculations.

## Data Availability

Crystal structure data (CCDC: 2222477 for **1**, 2222480 for **1a**, 2222481 for **2**, 2222482 for **2a**, 2222483 for **3**, 2222484 for **3m**, and 2222485 for **3a**) have been deposited in the CCDC.

## Supplementary Materials

Supplementary 1 Experimental section Figs. S1 to S41 Tables S1 to S19
