# Supplementary material for: Synergism of Light-Induced [4 + 4] Cycloaddition and Electron Transfer Toward Switchable Photoluminescence and Single-Molecule Magnet Behavior in a Dy4 Cubane
Source: Research (Wash D C). 2024 Jul 5;7:0411. doi: 10.34133/research.0411 (PMC11223772; doi:10.34133/research.0411)
Supplement: Supplementary 1 — Experimental section Figs. S1 to S41 Tables S1 to S19 [file research.0411.f1.docx]

**Supporting Information**

**Synergism of Light-Induced [4 + 4] Cycloaddition and Electron Transfer toward Switchable Photoluminescence and Single-Molecule Magnet Behavior in a Dy_4_ Cubane**

Yu-Han Wang,^1†^ Zhen-Ni Gao,^1†^ Shuai Liang,^1^ Jie-Li,^1^ Wu-Ji Wei,^1^ Song-De Han,^1^ Yi-Quan Zhang,^2^* Ji-Xiang Hu,^1^* and Guo-Ming Wang^1^*

^1^College of Chemistry and Chemical Engineering, Qingdao University, Qingdao, Shandong 266071, P. R. China.

^2^Jiangsu Key Laboratory for NSLSCS, School of Physical Science and Technology, Nanjing Normal University, Nanjing 210023, P. R. China.

E-mail: [gmwang_pub@163.com](mailto:gmwang_pub@163.com), hujixiang@qdu.edu.cn, zhangyiquan@njnu.edu.cn.

**Contents**

Experimental section

Figure S1. The TG plot of compound **1** under N_2_ atmosphere.

Figure S2. The TG plot of compound **2** under N_2_ atmosphere.

Figure S3. The TG plot of compound **3** under N_2_ atmosphere.

Figure S4. PXRD patterns of **1**, **1a** and **1a after grinding**.

Figure S5. PXRD patterns of **2**, **2a** and **2a after grinding**.

Figure S6. PXRD patterns of **3**, **3a** and **3a after grinding**.

Figure S7. Crystal structures of **2** (top) and **2a** (bottom) exhibiting the stacking of the anthracene in **2** before and after photodimerization to **2a**. Atomic scheme: Gd, green; C, gray 40%; O, red; and N, light blue.

Figure S8. Crystal structures of **3** (top) and **3a** (bottom) exhibiting the stacking of the anthracene in **3** before and after photodimerization to **3a**. Atomic scheme: Y, green; C, gray 40%; O, red; and N, light blue.

Figure S9**.** Color change images of compound **1** powder samples under different irradiation time.

Figure S10**.** Color change images of compound **2** powder samples under different irradiation time.

Figure S11**.** Color change images of compound **3** powder samples under different irradiation time.

Figure S12. Time-dependent UV-vis spectra of **2** in the solid state upon irradiation under ambient conditions. Insert: the photocoloration of the crystalline powder samples at different irradiation time.

Figure S12. Time-dependent UV-vis spectra of **3** in the solid state upon irradiation under ambient conditions. Insert: the photocoloration of the crystalline powder samples at different irradiation time.

Figure S14. Time-dependent fluorescent spectra of **2** at solid state upon light irradiation when excited at 360 nm.

Figure S15. Time-dependent fluorescent spectra of **3** at solid state upon light irradiation when excited at 360 nm.

Figure S16. The ESR spectra of **3**, **3a**, **3b** and **3b after irradiation** at solid state.

Figure S17. The ESR spectra of **2**, **2a**, **2b** and **2b after irradiation** at solid state.

Figure S18. IR plots for compounds **1**, **1a** and **1b**. Insert: enlarged IR spectrum between 1000 and 400 cm^−1^.

Figure S19. IR plots for compounds **2**, **2a** and **2b**. Insert: enlarged IR spectrum between 1000 and 400 cm^−1^.

Figure S20. IR plots for compounds **3**, **3a** and **3b**. Insert: enlarged IR spectrum between 1000 and 400 cm^−1^.

Figure S21. The schematic diagram of the proposed mechanism for the photo-induced production of compound **1**. The structural change parts are represented as black color.

Figure S22. (a) Color of **1** before irradiation (first), after irradiation (second), regrinding (third), and reillumination (fourth); (b) Solid-state luminescence color under UV light (365 nm) of powder sample of **1** before (first), after (second), regrinding (third), and reillumination (fourth).

Figure S23. (a) Color of **2** before irradiation (first), after irradiation (second), regrinding (third), and reillumination (fourth); (b) Solid-state luminescence color under UV light (365 nm) of powder sample of **2** before (first), after (second), regrinding (third), and reillumination (fourth).

Figure S24. (a) Color of **3** before irradiation (first), after irradiation (second), regrinding (third), and reillumination (fourth); (b) Solid-state luminescence color under UV light (365 nm) of powder sample of **3** before (first), after (second), regrinding (third), and reillumination (fourth).

Figure S25. UV-vis spectra of **2**, **2a**, **2b** and **2b after irradiation**.

Figure S26. UV-vis spectra of **3**, **3a**, **3b** and **3b after irradiation.**

Figure S27. Fluorescence spectra of **2**, **2a**, **2b** and **2b after irradiation.**

Figure S28. Fluorescence spectra of **3**, **3a**, **3b** and **3b after irradiation.**

Figure S29. The intermolecular H-bonding and C-H···π interactions among the cubane structures for **1**. Parts anthracene rings in which the carboxyl carbon atoms are labeled as black are omitted for clarity.

Figure S30. Temperature-dependent susceptibilities for **3a** under a dc field of 1000 Oe.

Figure S31**.** Temperature-dependent susceptibilities for **2** and **2a** under a dc magnetic field of 1000 Oe.

Figure S32. Field dependence of magnetization for **1** and **1a** at 2 K.

Figure S33. Field dependence of magnetization of **2** and **2a** at 2 K.

Figure S34. Temperature dependence of the in-phase and out-of-phase components of ac magnetic susceptibility for **1** in a zero-dc and 5 ac field at various ac frequencies.

Figure S35. Temperature dependence of the in-phase and out-of-phase components of ac magnetic susceptibility for **1a** in a zero-dc and 5 ac field at various ac frequencies.

Figure S36. Field dependence of the *χ*" component of ac susceptibility components for **1a** with *ω* = 100 Hz at 2 K.

Figure S37. Frequency dependence of the in-phase components of **1a** in a 1200 Oe dc field between 2–10 K.

Figure S38. Frequency dependence of the out-of-phase components of **1a** in a 1200 Oe dc field between 2–10 K.

Figure S39. Calculated eight individual Dy^Ⅲ^ fragments of **1-Dy1**, **1-Dy2**, **1-Dy3**, **1-Dy4**, **1a-Dy1**, **1a-Dy2**, **1a-Dy3** and **1a-Dy4**; H atoms are omitted for clarify.

Figure S40. Scheme of the Dy^Ⅲ^-Dy^Ⅲ^ and Dy^Ⅲ^-radical interactions in **1** and **1a**.

Figure S41. Calculated orientations of the local main magnetic axes on Dy^III^ ions of **1** and **1a** in their ground KDs.

Table S1. Crystallographic data for compounds **1** and **1a**.

Table S2. Crystallographic data for compounds **2**, **2a**, **3**, **3m** and **3a**.

Table S3. Continuous Shape Measure (CShM) analyses of geometries for compound **1** by SHAPE 2.0 Software.

Table S4. Continuous Shape Measure (CShM) analyses of geometries for compound **1a** by SHAPE 2.0 Software.

Table S5. Continuous Shape Measure (CShM) analyses of geometries for compound **2** by SHAPE 2.0 Software.

Table S6. Continuous Shape Measure (CShM) analyses of geometries for compound **2a** by SHAPE 2.0 Software.

Table S7. Continuous Shape Measure (CShM) analyses of geometries for compound **3** by SHAPE 2.0 Software.

Table S8. Continuous Shape Measure (CShM) analyses of geometries for compound **3m** by SHAPE 2.0 Software.

Table S9. Continuous Shape Measure (CShM) analyses of geometries for compound **3a** by SHAPE 2.0 Software.

Table S10. Selected bond lengths (Å) and angles (^o^) for compound **1** at 180(2) K.

Table S11. Selected bond lengths (Å) and angles (^o^) for compound **1a** at 120(2) K.

Table S12. Selected bond lengths (Å) and angles (^o^) for compound **2** at 293(2) K.

Table S13. Selected bond lengths (Å) and angles (^o^) for compound **2a** at 293(2) K.

Table S14. Selected bond lengths (Å) and angles (^o^) for compound **3** at 293(2) K.

Table S15. Selected bond lengths (Å) and angles (^o^) for compound **3m** at 293(2) K.

Table S16. Selected bond lengths (Å) and angles (^o^) for compound **3a** at 293(2) K.

Table S17. Calculated energy levels (cm^−1^), ***g*** (*g_x_*, *g_y_*, *g_z_*) tensors and predominant *m_J_* values of the lowest eight Kramers doublets (KDs) of individual Dy^Ⅲ^ fragments for **1** and **1a** using CASSCF/RASSI-SO with the OpenMolcas.

Table S18. Wave functions with definite projection of the total moment | *m_J_* > for the lowest eight KDs of individual Dy^Ⅲ^ fragments for **1** and **1a** using CASSCF/RASSI-SO with the OpenMolcas.

Table S19. Exchange energies *E* (cm^−1^), the energy differences between each exchange doublet Δ*_t_* (cm^−1^) and the main values of the *g_z_* for the lowest 8 and 24 exchange doublets arising from the exchange interactions on magnetic centers **1** and **1a**, respectively.





**Figure S1.** The TG plot of compound **1** under N_2_ atmosphere.





**Figure S2.** The TG plot of compound **2** under N_2_ atmosphere.





**Figure S3.** The TG plot of compound **3** under N_2_ atmosphere.





**Figure S4.** PXRD patterns of **1**, **1a** and **1a after grinding**.

**

**

**Figure S5.** PXRD patterns of **2**, **2a** and **2a after grinding**.





**Figure S6.** PXRD patterns of **3**, **3a** and **3a after grinding**.


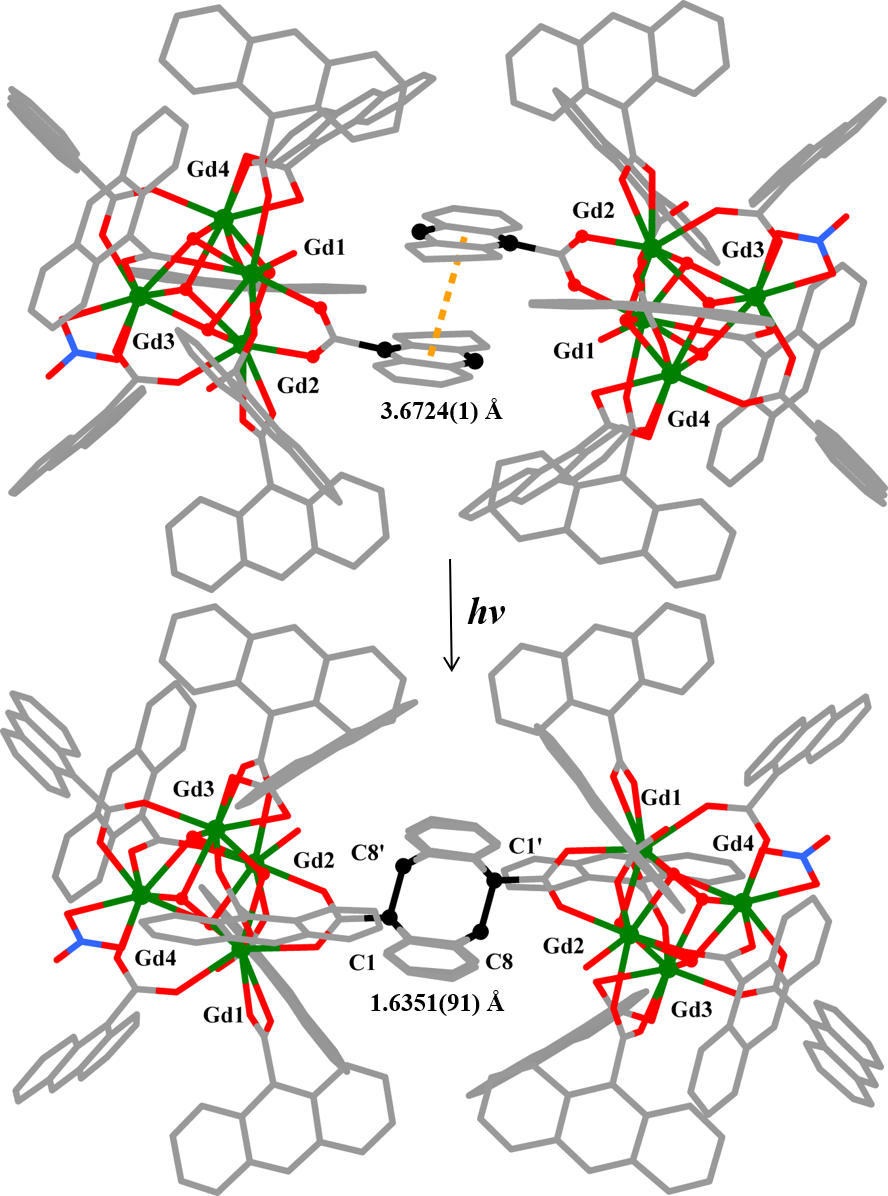


**Figure S7.** Crystal structures of **2** (top) and **2a** (bottom) exhibiting the stacking of the anthracene in **2** before and after photodimerization to **2a**. Atomic scheme: Gd, green; C, gray 40%; O, red; and N, light blue.


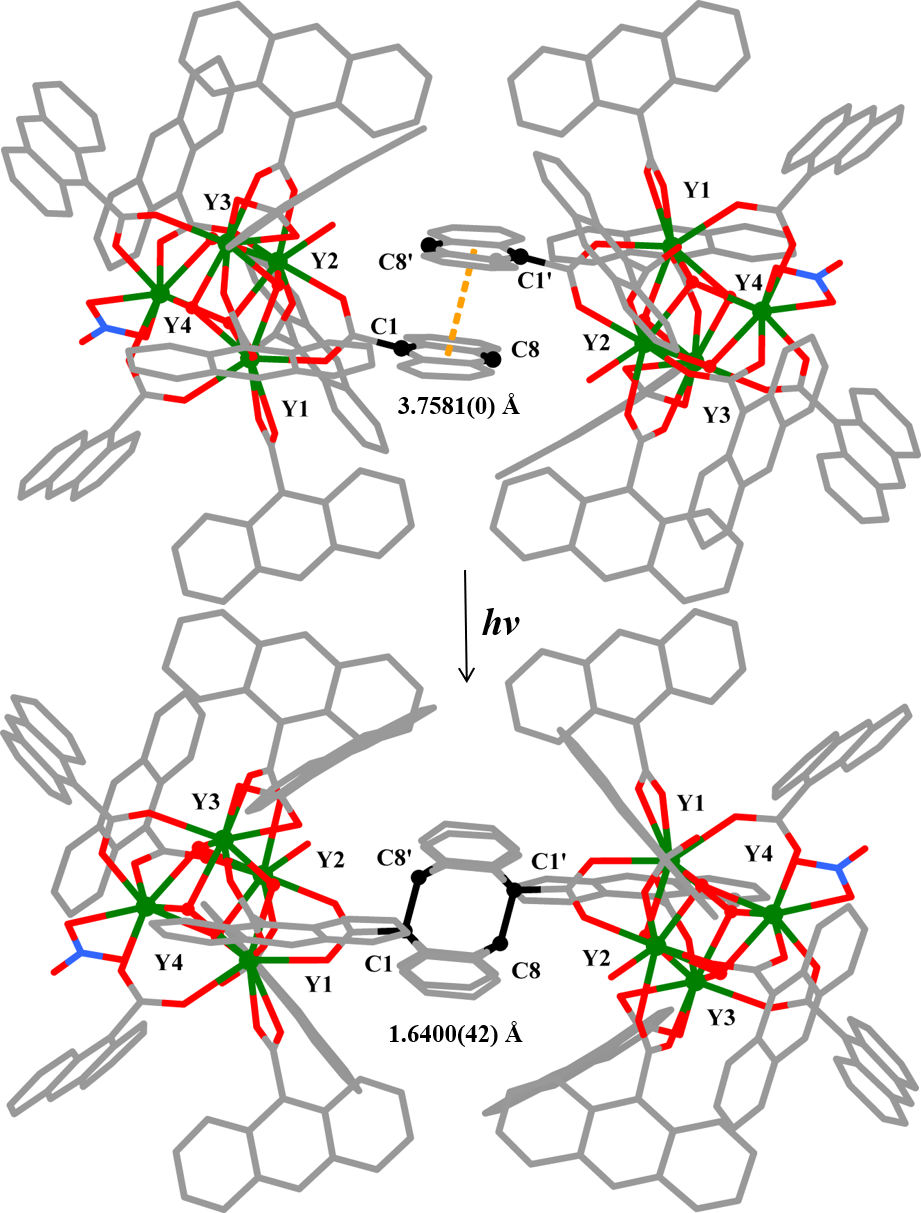


**Figure S8.** Crystal structures of **3** (top) and **3a** (bottom) exhibiting the stacking of the anthracene in **3** before and after photodimerization to **3a**. Atomic scheme: Y, green; C, gray 40%; O, red; and N, light blue.


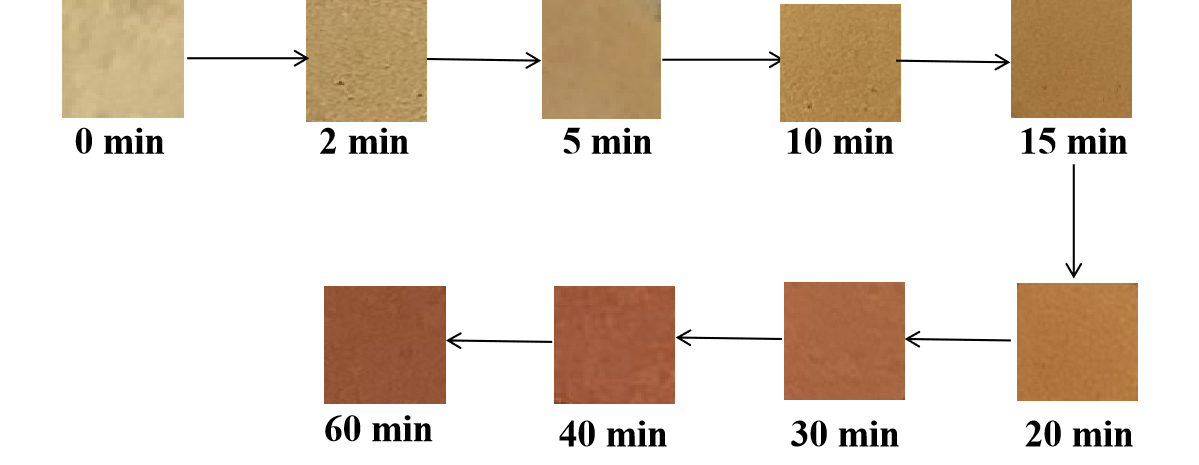


**Figure S9.** Color change images of compound **1** powder samples under different irradiation time.


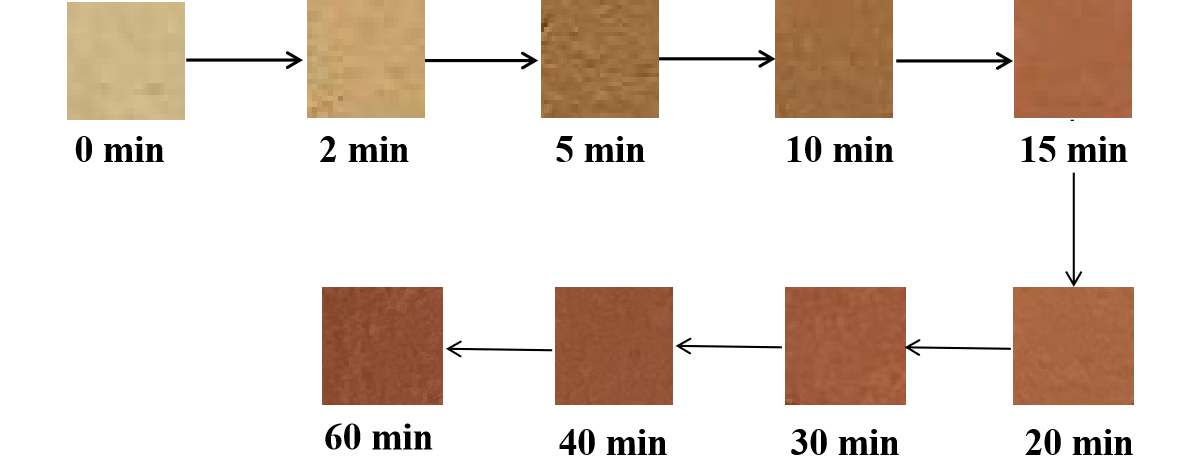


**Figure S10.** Color change images of compound **2** powder samples under different irradiation time.


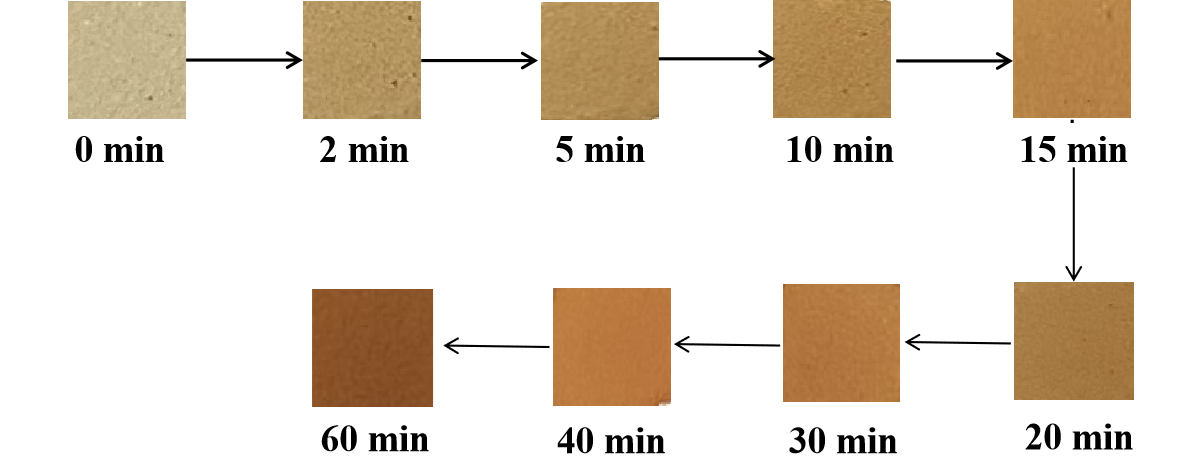


**Figure S11.** Color change images of compound **3** powder samples under different irradiation time.


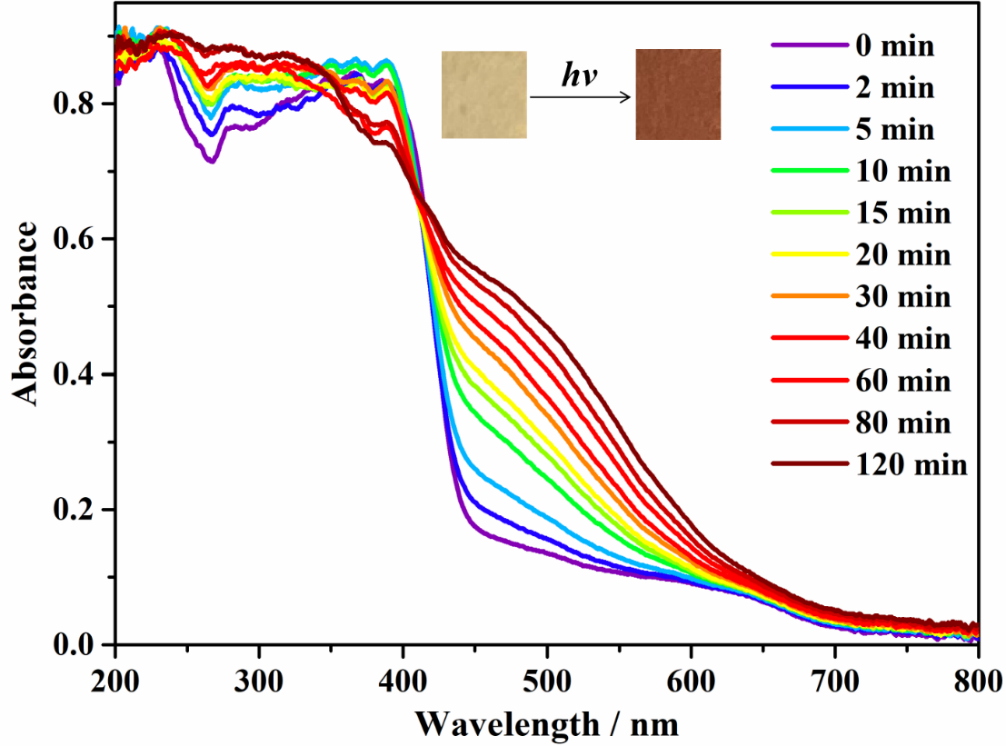


**Figure S12.** Time-dependent UV-vis spectra of **2** in the solid state upon irradiation under ambient conditions. Insert: the photocoloration of the crystalline powder samples at different irradiation time.


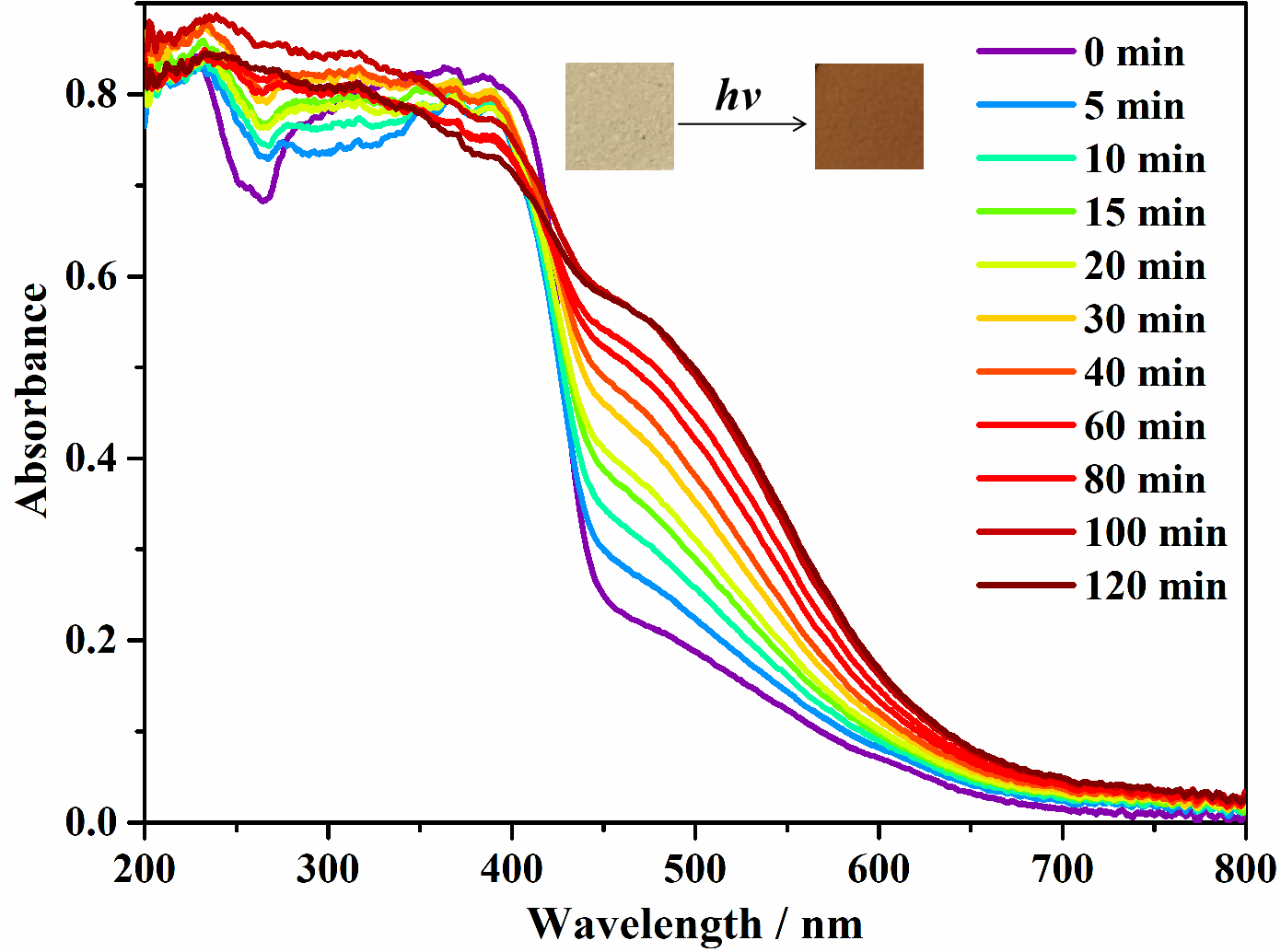


**Figure S13.** Time-dependent UV-vis spectra of **3** in the solid state upon irradiation under ambient conditions. Insert: the photocoloration of the crystalline powder samples at different irradiation time.

**
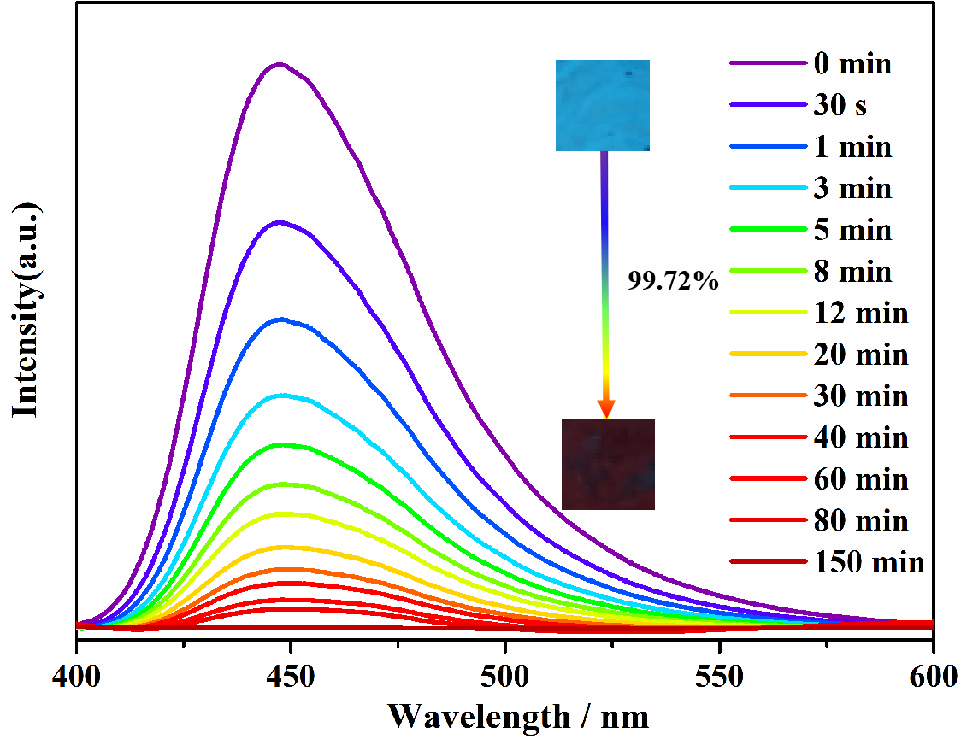
**

**Figure S14.** Time-dependent fluorescent spectra of **2** at solid state upon light irradiation when excited at 360 nm.


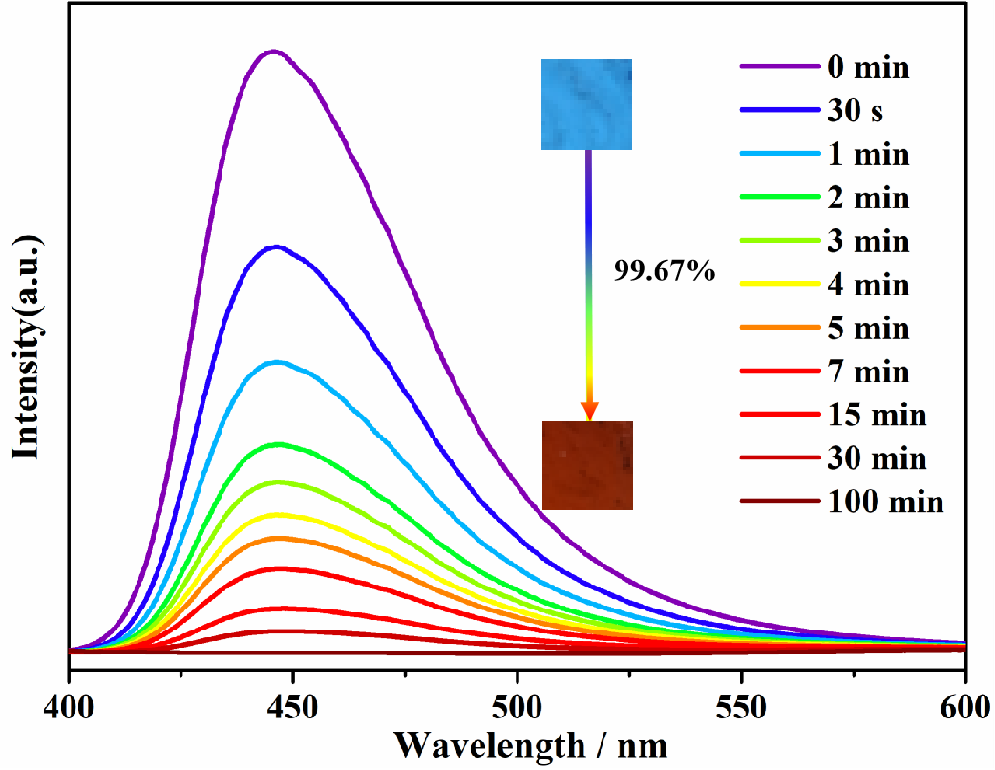


**Figure S15.** Time-dependent fluorescent spectra of **3** at solid state upon light irradiation when excited at 360 nm.





**Figure S16.** The ESR spectra of **3, 3a, 3b** and **3b after irradiation** at solid state.





**Figure S17.** The ESR spectra of **2, 2a, 2b** and **2b after irradiation** at solid state.


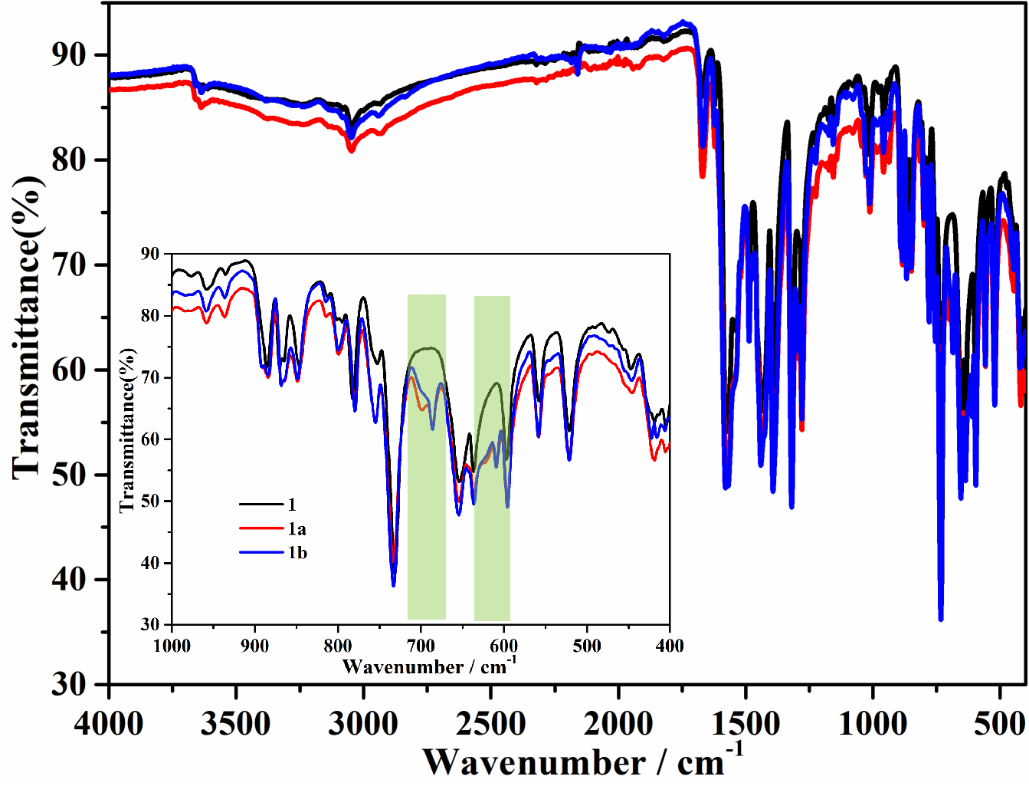


**Figure S18**. IR plots for compounds **1**, **1a** and **1b**. Insert: enlarged IR spectrum between 1000 and 400 cm^-1^.


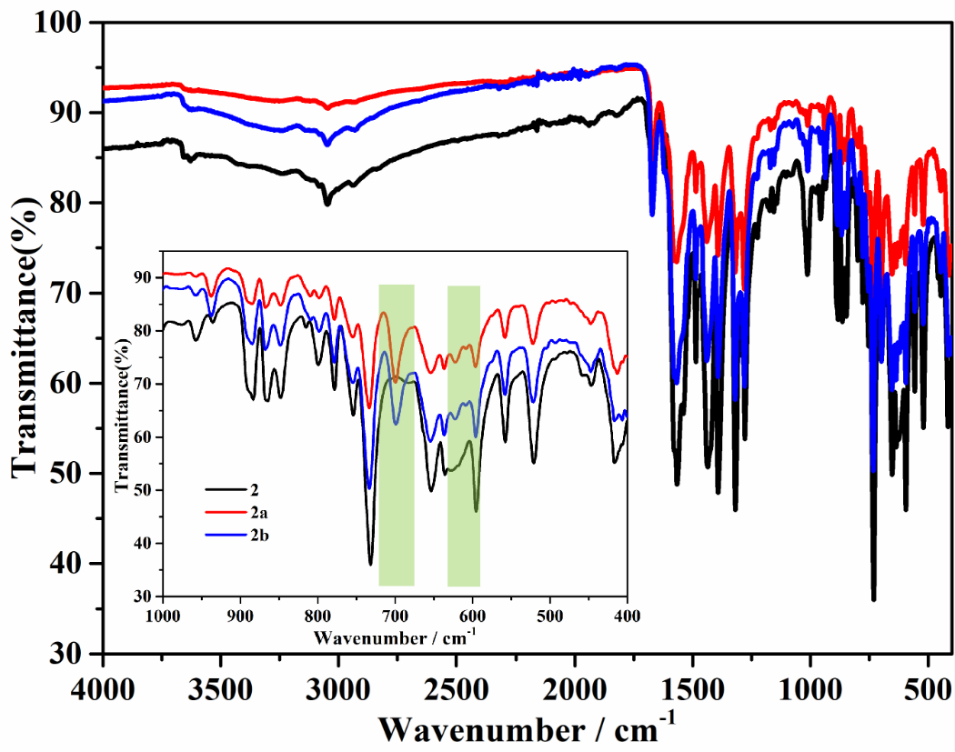


**Figure S19**. IR plots for compounds **2**, **2a** and **2b**. Insert: enlarged IR spectrum between 1000 and 400 cm^-1^.


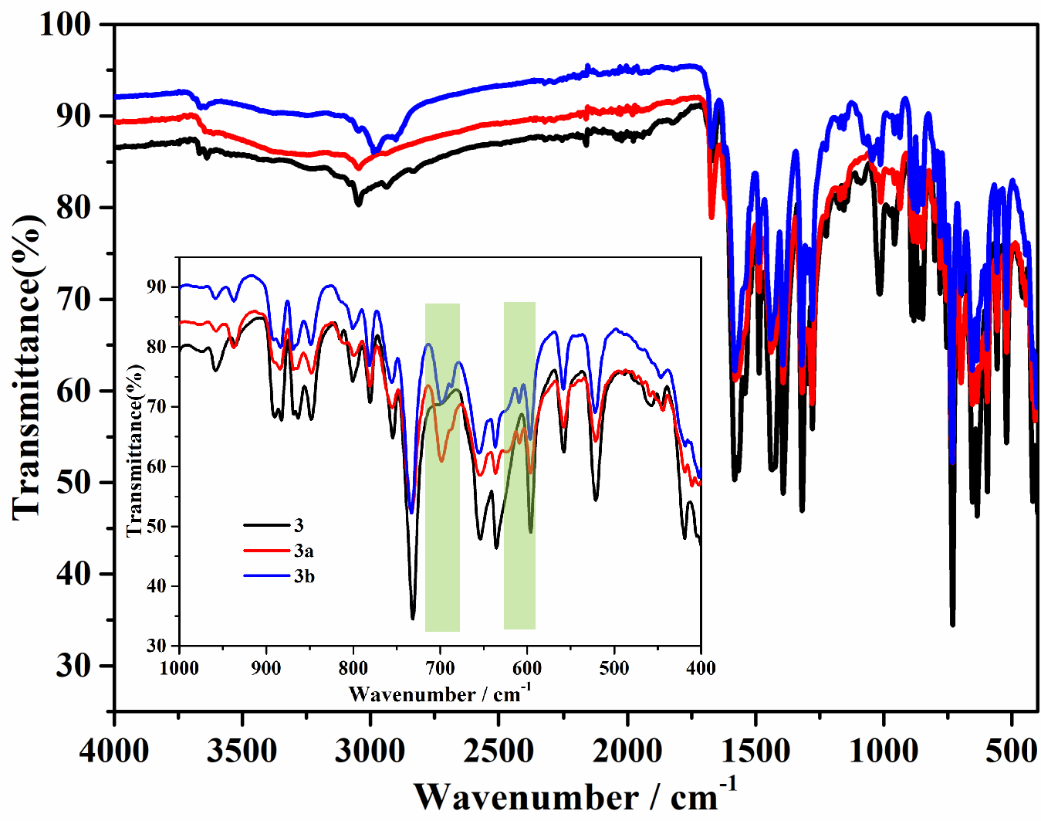


**Figure S20**. IR plots for compounds **3**, **3a** and **3b**. Insert: enlarged IR spectrum between 1000 and 400 cm^–1^.


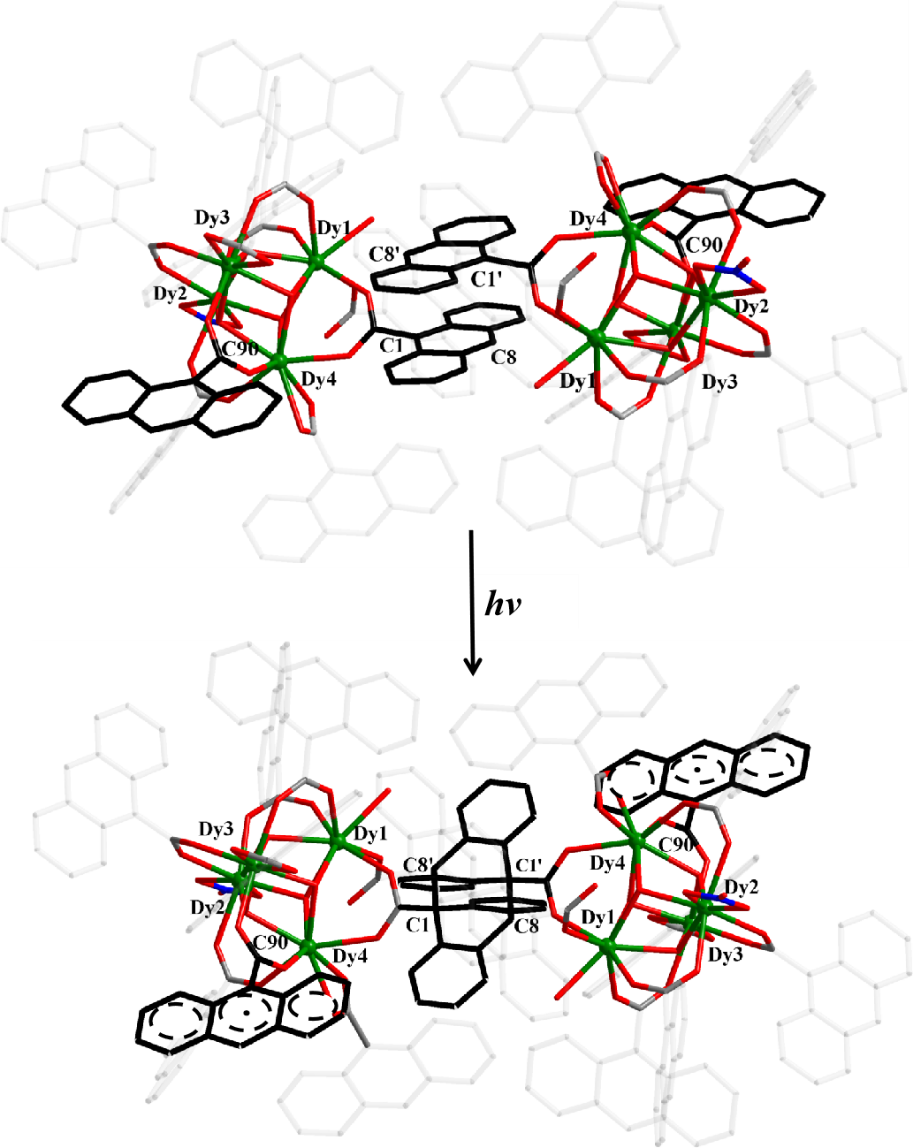


**Figure S21.** The schematic diagram of the proposed mechanism for the photo-induced production of compound **1**. The structural change parts are represented as black color.


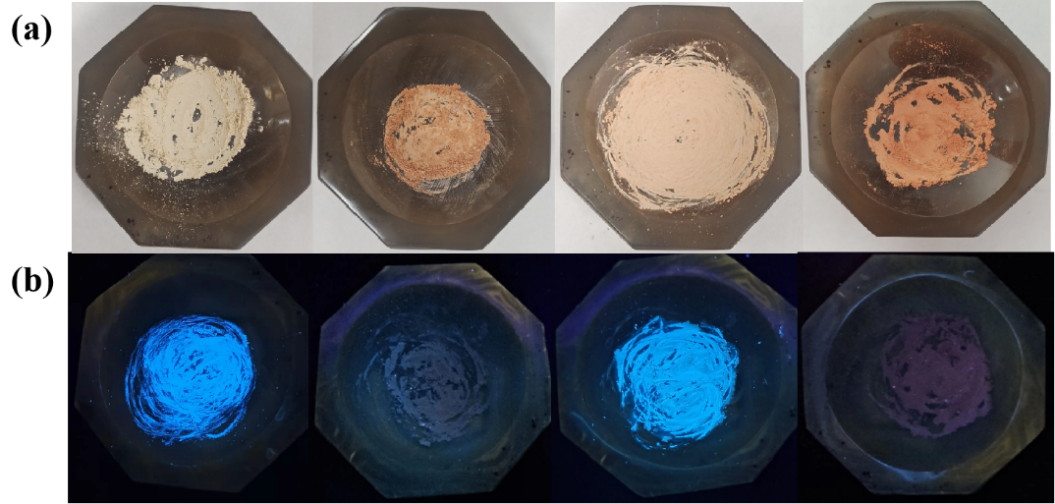


**Figure S22.** (a) Color of **1** before irradiation (first), after irradiation (second), regrinding (third), and reillumination (fourth); (b) Solid-state luminescence color under UV light (365 nm) of powder sample of **1** before (first), after (second), regrinding (third), and reillumination (fourth).


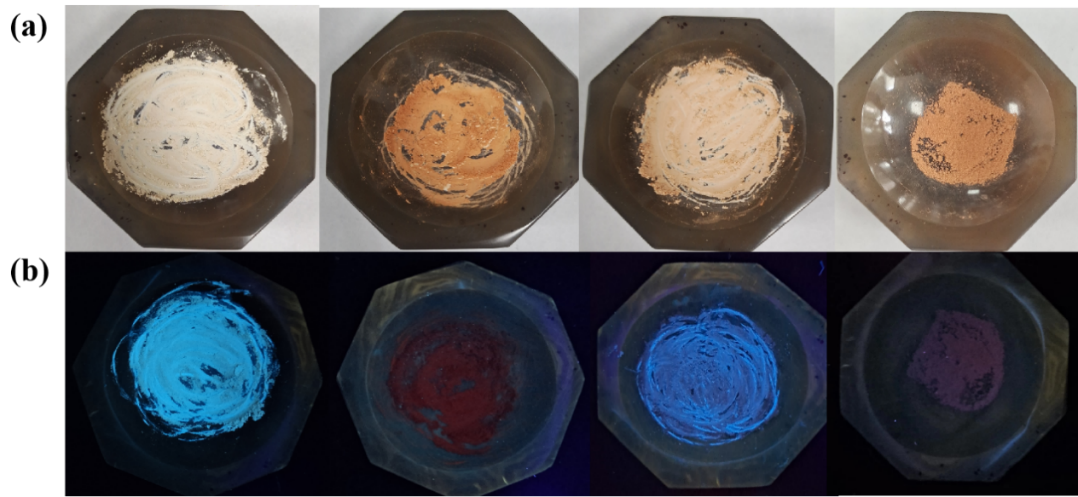


**Figure S23.** (a) Color of **2** before irradiation (first), after irradiation (second), regrinding (third), and reillumination (fourth); (b) Solid-state luminescence color under UV light (365 nm) of powder sample of **2** before (first), after (second), regrinding (third), and reillumination (fourth).


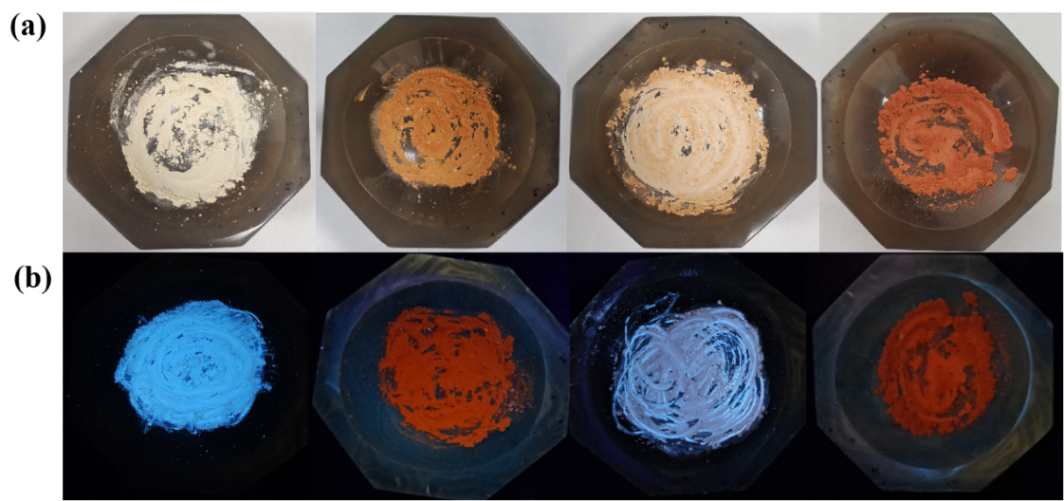


**Figure S24.** (a) Color of **3** before irradiation (first), after irradiation (second), regrinding (third), and reillumination (fourth); (b) Solid-state luminescence color under UV light (365 nm) of powder sample of **3** before (first), after (second), regrinding (third), and reillumination (fourth).





**Figure S25.** UV-vis spectra of **2**, **2a**, **2b** and **2b after irradiation**.





**Figure S26.** UV-vis spectra of **3**, **3a**, **3b** and **3b after irradiation**.





**Figure S27.** Fluorescence spectra of **2**, **2a**, **2b** and **2b after irradiation**.





**Figure S28.** Fluorescence spectra of **3**, **3a**, **3b** and **3b after irradiation**.

**
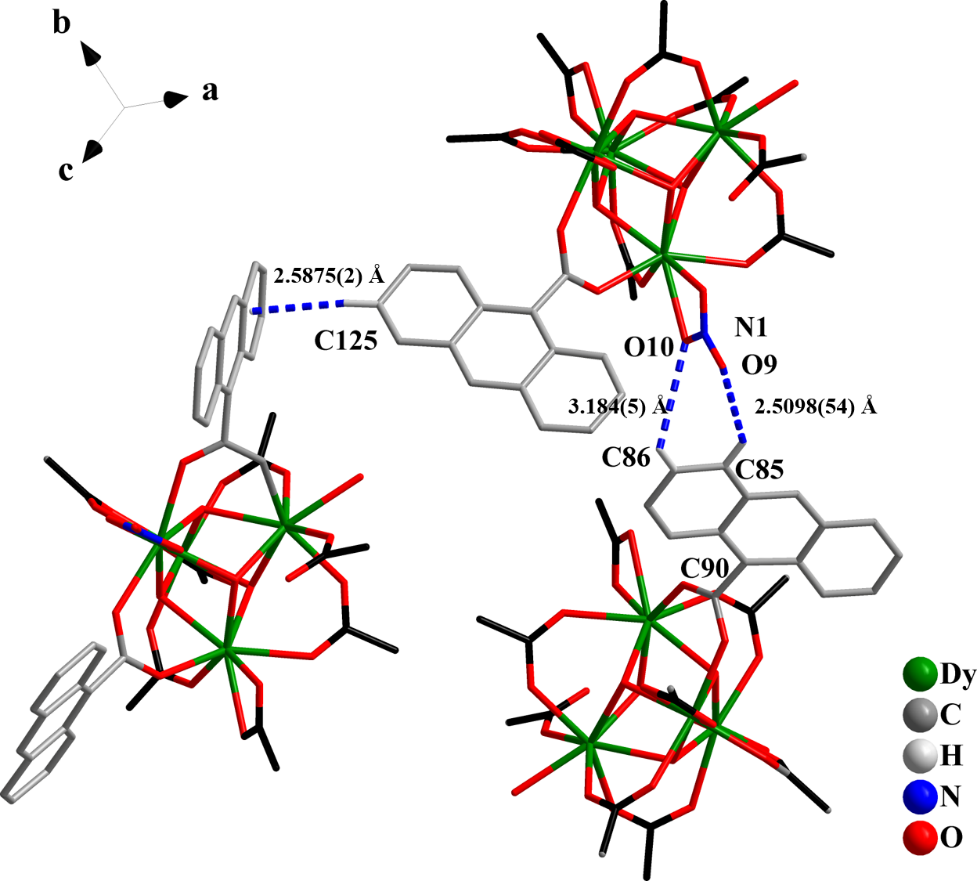
**

**Figure S29.** The intermolecular H-bonding and C-H···π interactions among the cubane structures for **1**. Parts anthracene rings in which the carboxyl carbon atoms are labeled as black are omitted for clarity.


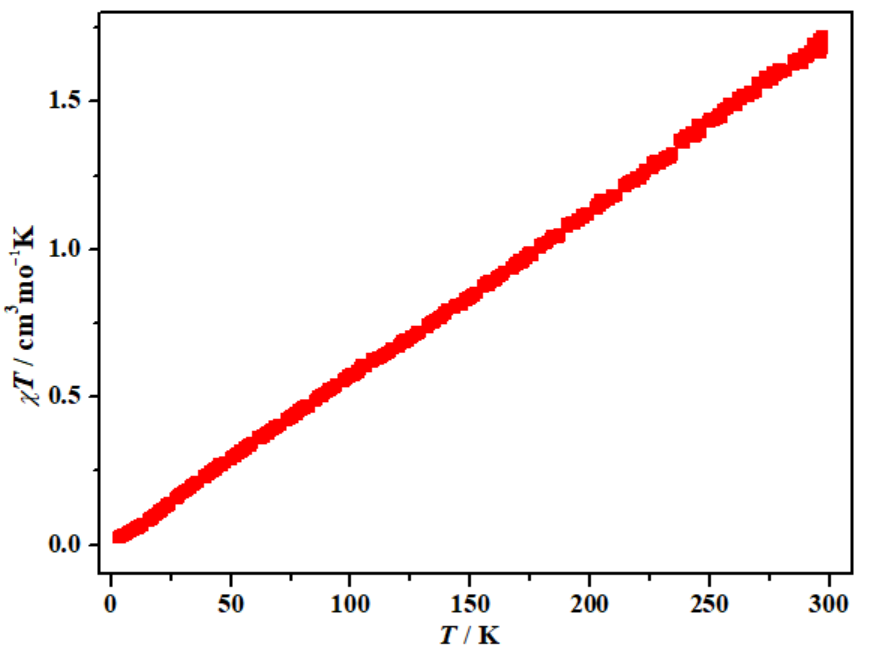


**Figure S30.** Temperature-dependent susceptibilities for **3a** under a dc field of 1000 Oe.





**Figure S31.** Temperature-dependent susceptibilities for **2** and **2a** under a dc magnetic field of 1000 Oe.





**Figure S32.** Field dependence of magnetization for **1** and **1a** at 2 K.


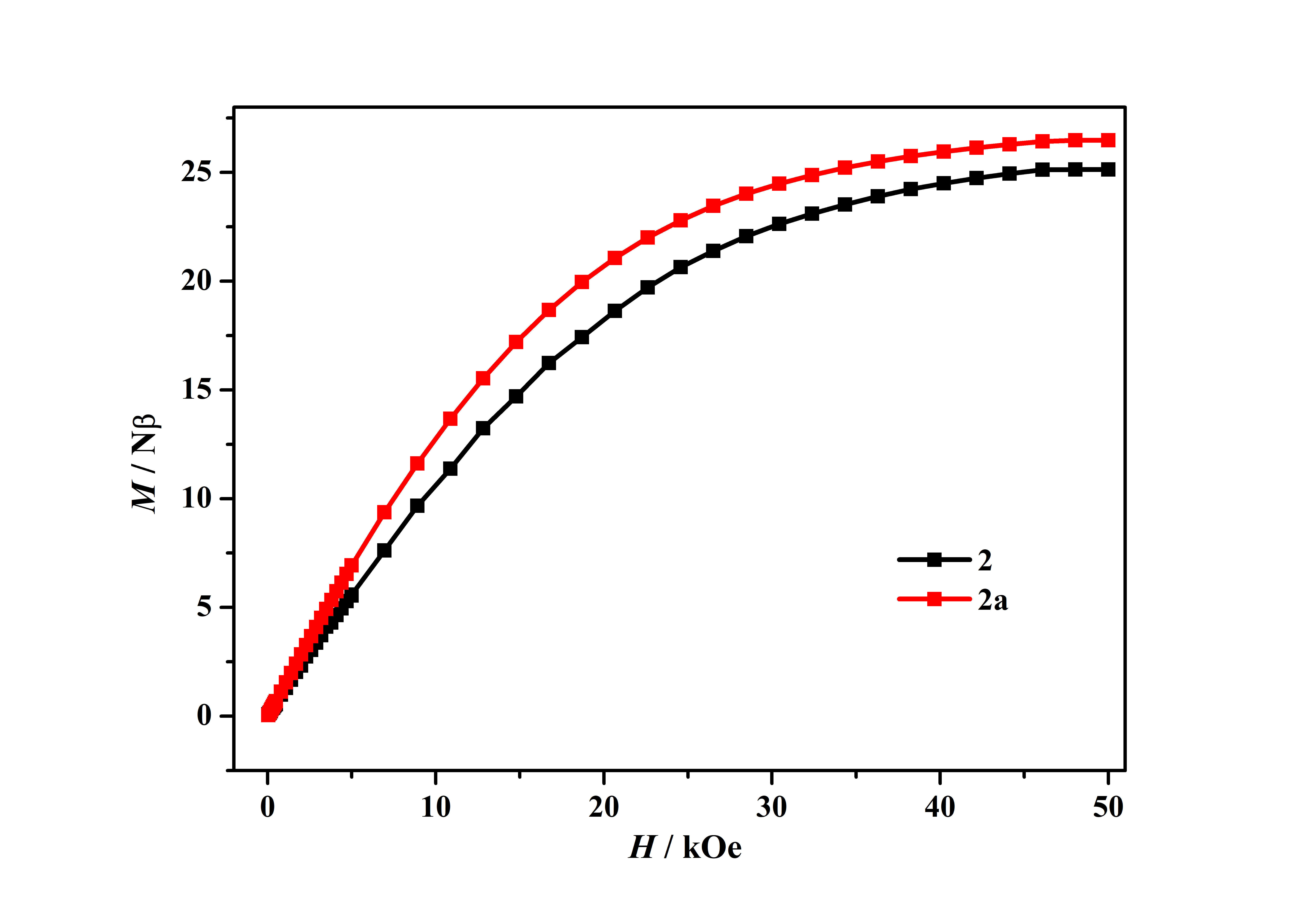


**Figure S33.** Field dependence of magnetization of **2** and **2a** at 2 K.


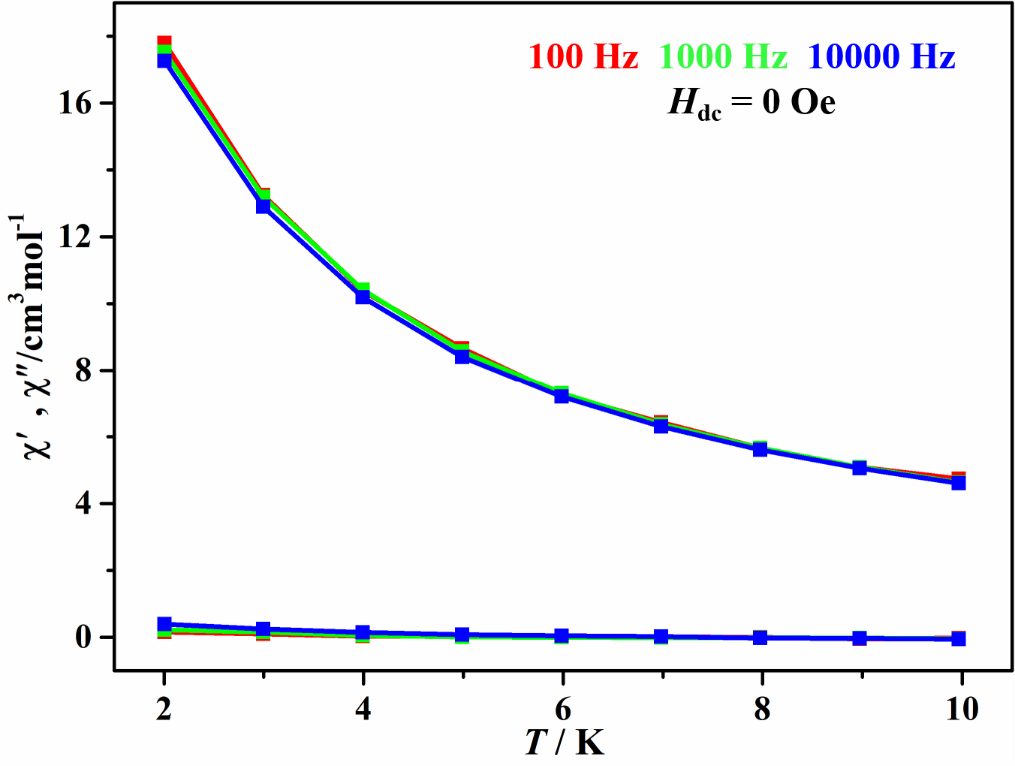


**Figure S34.** Temperature dependence of the in-phase and out-of-phase components of ac magnetic susceptibility for **1** in a zero-dc and 5 ac field at various ac frequencies.


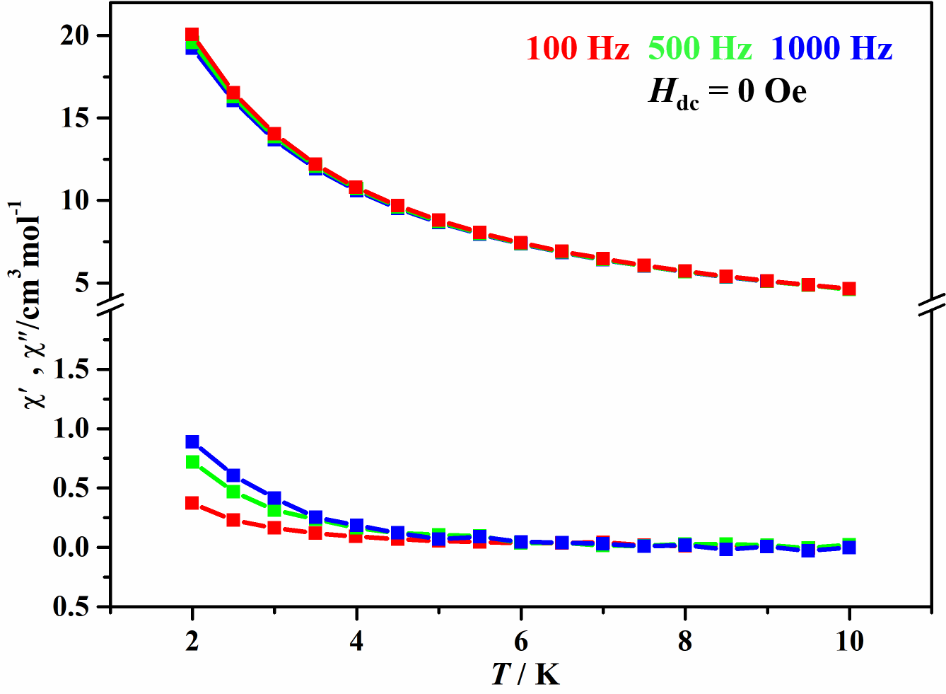


**Figure S35.** Temperature dependence of the in-phase and out-of-phase components of ac magnetic susceptibility for **1a** in a zero-dc and 5 ac field at various ac frequencies.





**Figure S36.** Field dependence of the *χ*" component of ac susceptibility components for **1a** with *ω* = 100 Hz at 2 K.


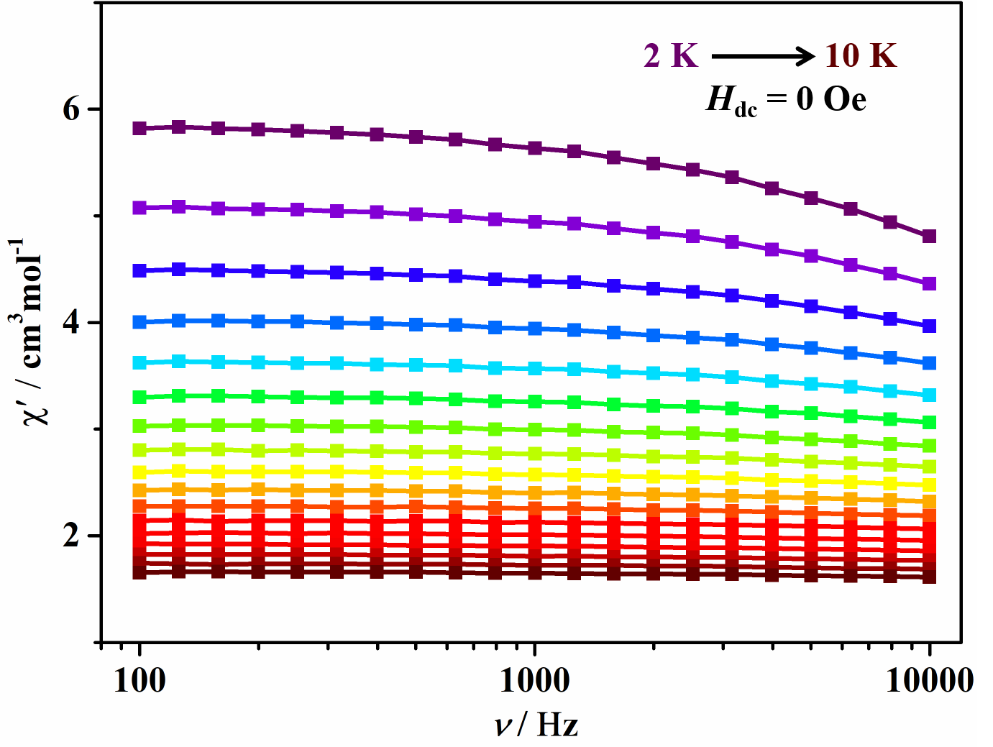


**Figure S37.** Frequency dependence of the in-phase components of **1a** in a 1200 Oe dc field between 2–10 K.


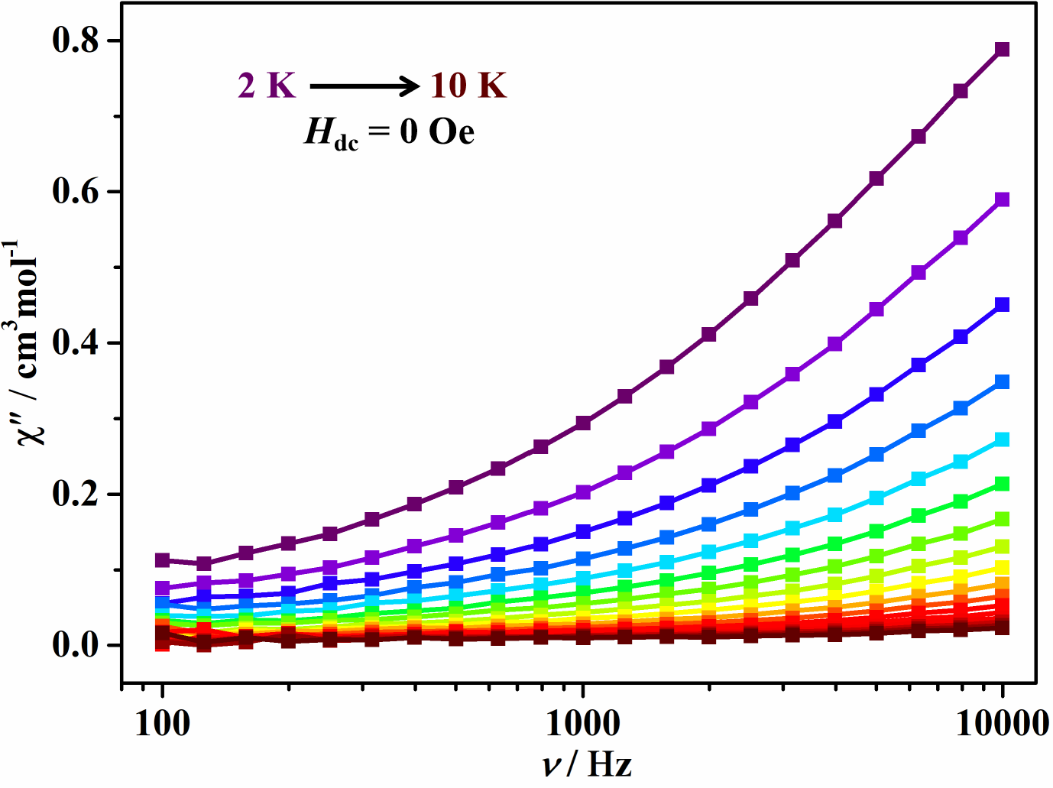


**Figure S38.** Frequency dependence of the out-of-phase components of **1a** in a 1200 Oe dc field between 2–10 K.


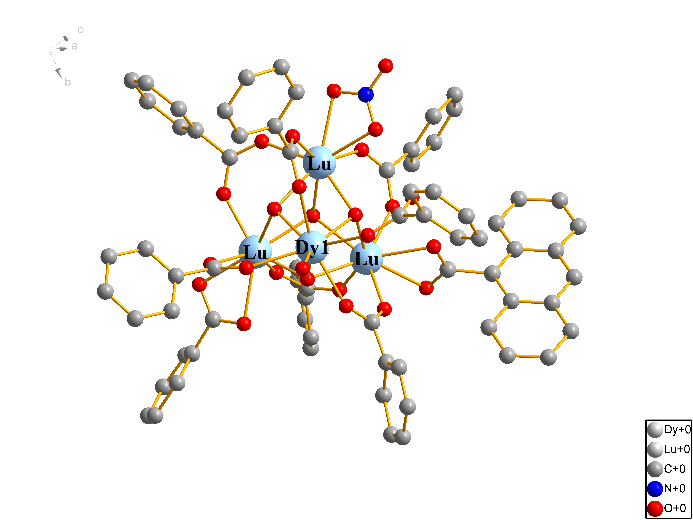

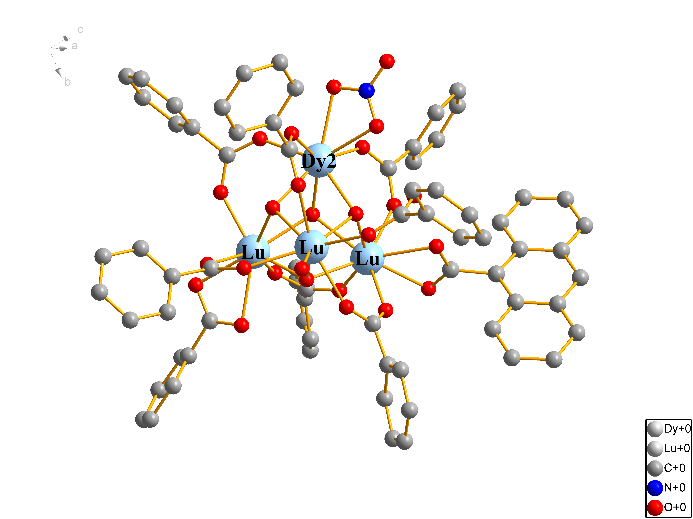


**1**-**Dy1 1**-**Dy2**


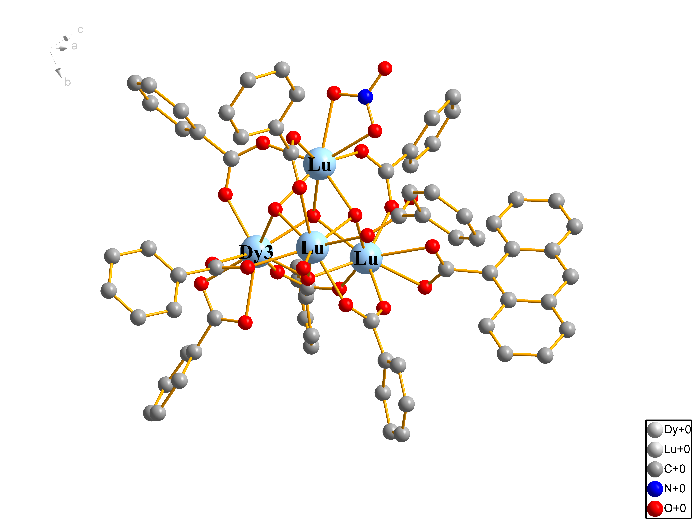

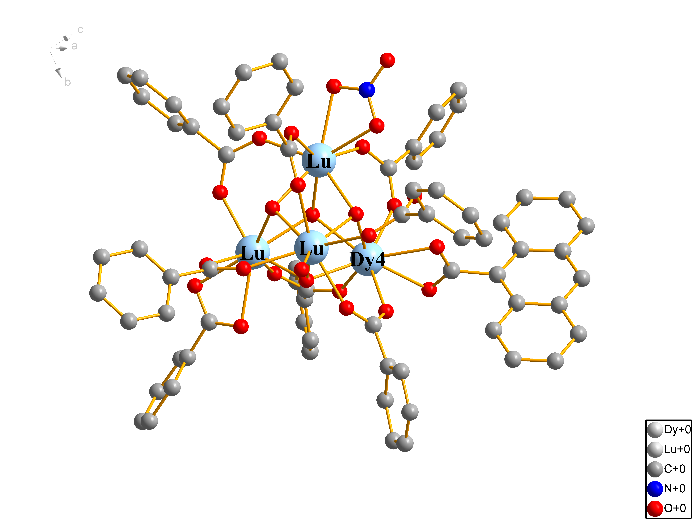


**1**-**Dy3 1**-**Dy4**


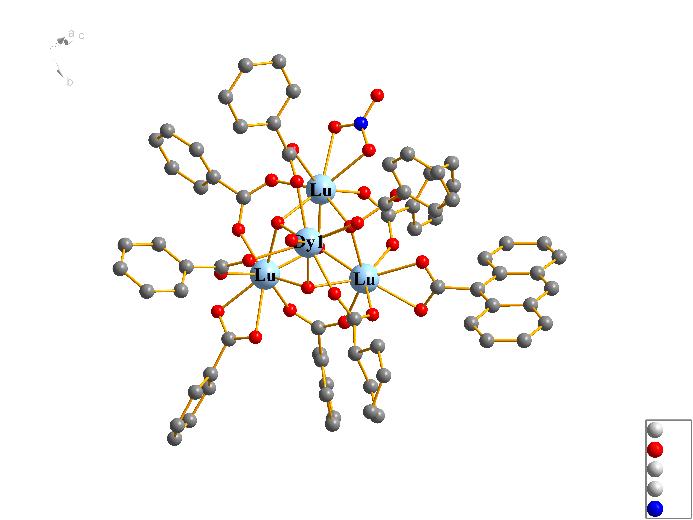

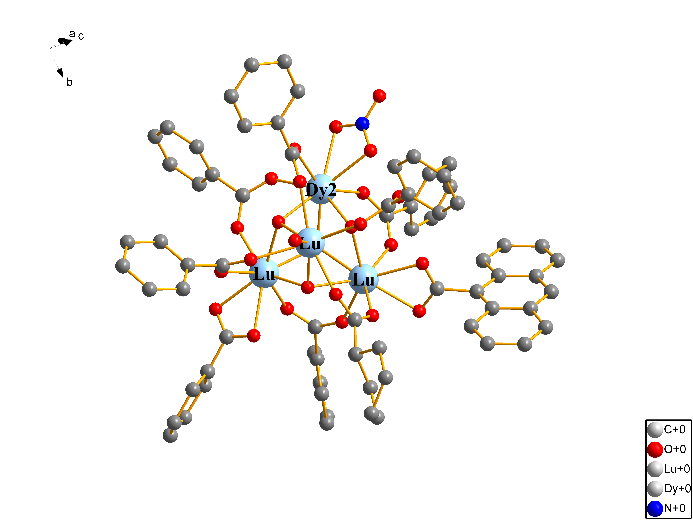


**1a**-**Dy1 1a**-**Dy2**


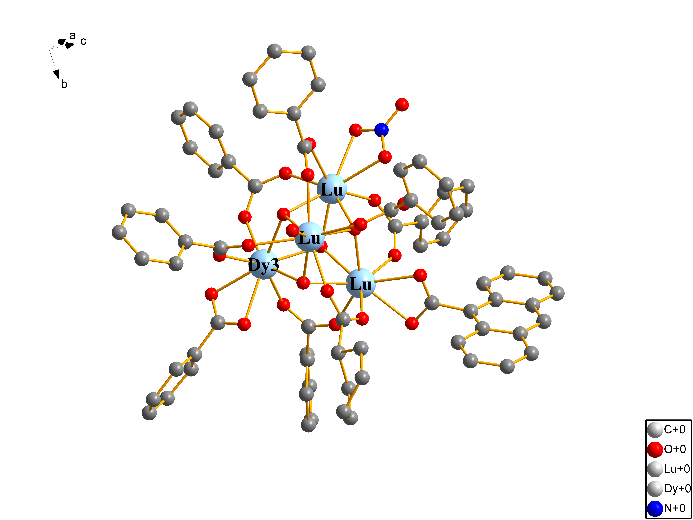

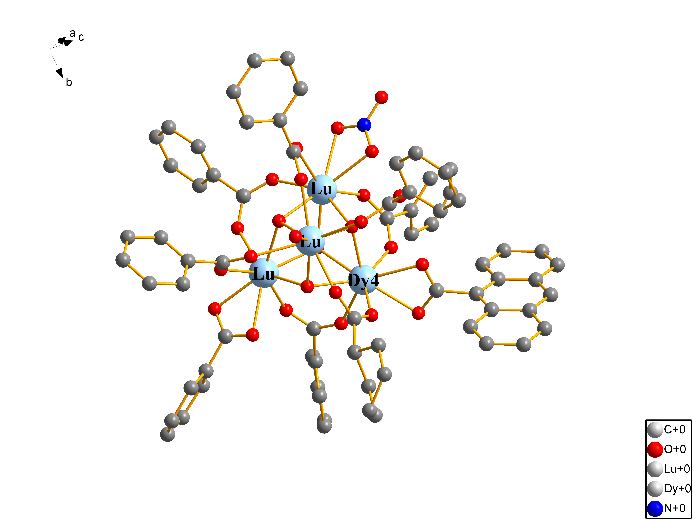


**1a**-**Dy3 1a**-**Dy4**

**Figure S39.** Calculated eight individual Dy^Ⅲ^ fragments of **1-Dy1**, **1-Dy2**, **1-Dy3**, **1-Dy4**, **1a-Dy1**, **1a-Dy2**, **1a-Dy3** and **1a-Dy4**; H atoms are omitted for clarify.


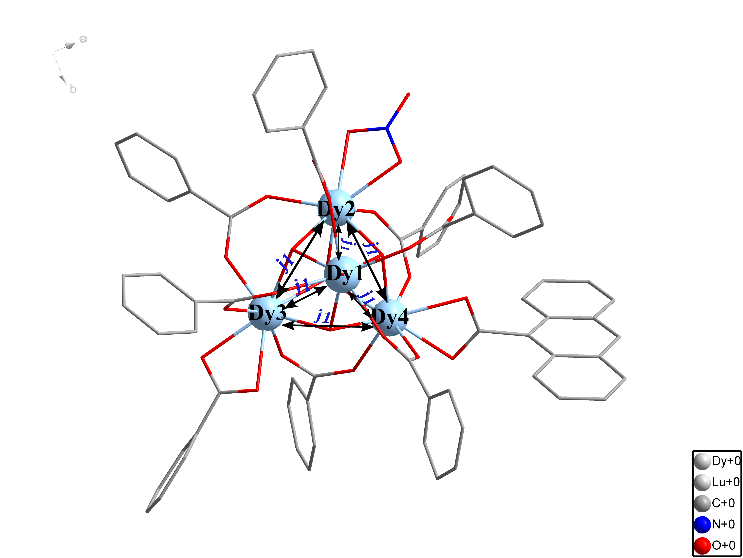

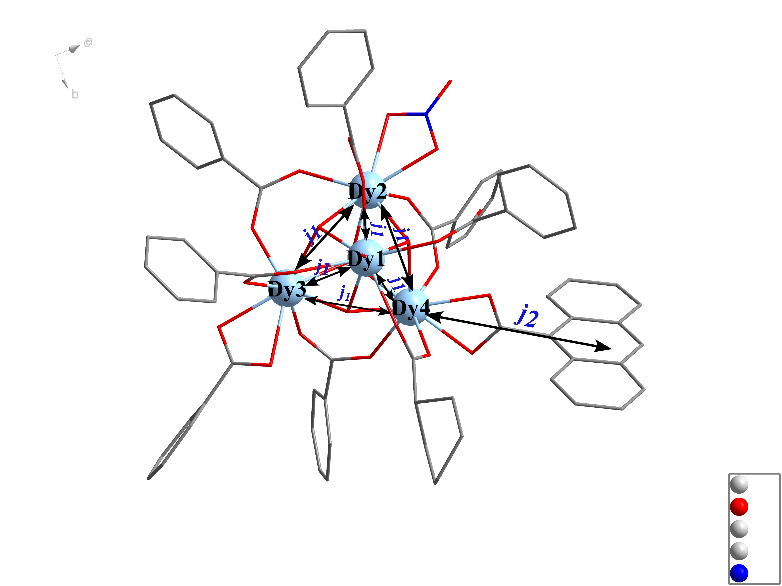


**1 1a**

**Figure S40**. Scheme of the Dy^Ⅲ^-Dy^Ⅲ^ and Dy^Ⅲ^-radical interactions in **1** and **1a**.

The Dy^III^−Dy^III^ and Dy^II^−radical exchange interactions in **1** and **1a** were fitted in two steps as follows. First the individual Dy^III^ fragments were calculated using CASSCF/RASSI-SO to obtain their magnetic properties. Then, the exchange interactions between the magnetic centers were considered within the Lines model,^R1^ which is effective and has been successfully used in the research of SMMs of *d* and *f*-block elements.^R2, R3^ The Dy^III^−Dy^III^ coupling interactions were considered the same because of the similar coordination environments of the Dy^III^ centers and their proximities. Meanwhile, the radical–radical interactions were not fitted because they were very weak due to the large distance between the radicals.

The Ising total Hamiltonian for **1** and **1a** were expressed as (1) and (2), respectively:

 (1)

 (2)

where$\hat{\tilde{S}}_{Dy}$and $\hat{S}_{\text{rad}}$ are the spin operators of four magnetic Dy^III^ centers and the radical, respectively, $\tilde{J}_{1}$ is the parameter of the effective magnetic coupling constant for the Dy^III^–Dy^III^ and $\tilde{J}_{2}$ is the parameter of the effective magnetic coupling constant for Dy^III^–radical, interaction with respect to the ground pseudospin of $\hat{S}_{\text{Dy}}$ = 1/2 on the Dy^III^ site and the spin of $\hat{S}_{\text{rad}}$ = 1 on the radical. The Lines exchange coupling constants *J* were fitted by comparing of the computed and measured magnetic susceptibilities using the POLY_ANISO program.^R1, R4, R5^

References

R1. M. E. Lines, Orbital Angular Momentum in the Theory of Paramagnetic Clusters. Orbital Angular Momentum in the Theory of Paramagnetic Clusters. *J. Chem. Phys*. **1971**, *55*, 2977−2984.

R2. K. C. Mondal, A. Sundt, Y. H. Lan, G. E. Kostakis, O. Waldmann, L. Ungur, L. F. Chibotaru, C. E. Anson, A. K. Powell, Coexistence of Distinct Single-Ion and Exchange-Based Mechanisms for Blocking of Magnetization in a Co^II^_2_Dy^III^_2_ Single-Molecule Magnet. *Angew. Chem. Int. Ed*. **2012**, *51*, 7550−7554.

R3. S. K. Langley, D. P. Wielechowski, V. Vieru, N. F. Chilton, B. Moubaraki, B. F. Abrahams, L. F. Chibotaru, K. S. Murray, A {Cr^III^_2_Dy^III^_2_} Single-Molecule Magnet: Enhancing the Blocking Temperature through 3d Magnetic Exchange. *Angew. Chem. Int. Ed*. **2013**, *52*, 12014–12019.

R4. L. Ungur, W. Van den Heuvel, L. F. Chibotaru, Ab initio investigation of the non-collinear magnetic structure and the lowest magnetic excitations in dysprosium triangles. *New J. Chem*. **2009**, *33*, 1224−1230.

R5. L. F. Chibotaru, L. Ungur, C. Aronica, H. Elmoll, G. Pilet, D. Luneau, Structure, Magnetism, and Theoretical Study of a Mixed-Valence Co^II^_3_Co^III^_4_ Heptanuclear Wheel: Lack of SMM Behavior despite Negative Magnetic Anisotropy. *J. Am. Chem. Soc*. **2008**, *130*, 12445−12455.


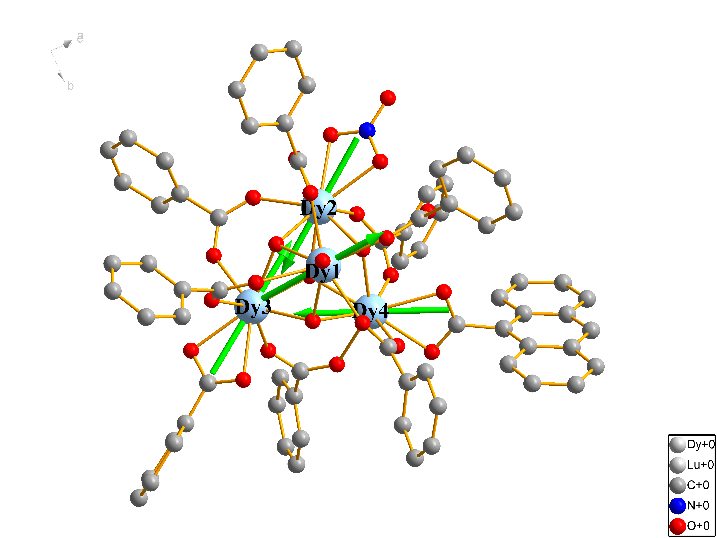

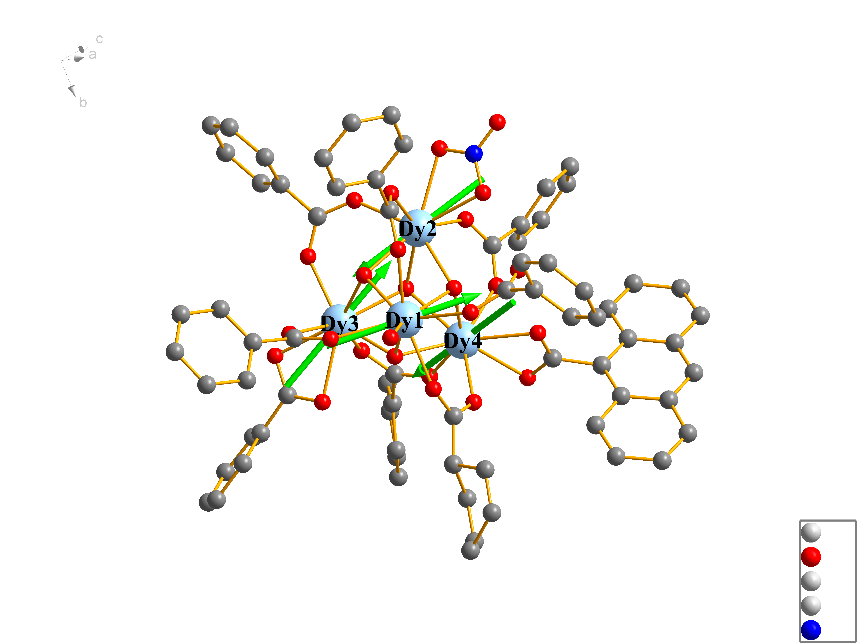


1. **1a**

**Figure S41**. Calculated orientations of the local main magnetic axes on Dy^III^ ions of **1** and **1a** in their ground KDs.

**Table S1**. Crystallographic data for compounds **1** and **1a**.

|  | **1** | **1a** |
| --- | --- | --- |
| Formula | C_135_H_95_Dy_4_NO_29_ | C_135_H_95_Dy_4_NO_29_ |
| *M*r (g·mol^-1^) | 2845.11 | 2845.11 |
| Temperature | 180(2) K | 120(2) K |
| Space group | *P*2_1_/*n* | *P*2_1_/*n* |
| Crystal system | Monoclinic | Monoclinic |
| *a* (Å) | 19.0929(14) | 19.0742(4) |
| *b* (Å) | 26.7829(17) | 26.6163(6) |
| *c* (Å) | 24.9829(18) | 24.9581(6) |
| *α* (*°*) | 90 | 90 |
| *β* (*°*) | 91.547(3) | 91.5030(10) |
| γ (*°*) | 90 | 90 |
| *V* (Å^3^) | 12770.7(15) | 12666.5(5) |
| *Z* | 4 | 4 |
| *F* (000) | 5632 | 5632 |
| *Dc* (gcm^-3^) | 1.480 | 1.492 |
| *μ* (mm^-1^) | 2.386 | 2.406 |
| *R*_int_ | 0.0617 | 0.0344 |
| limiting indices | -22<=h<=24  -34<=k<=34  -32<=l<=28 | -24<=h<=21  -29<=k<=34  -32<=l<=32 |
| Collected reflections | 103790 | 111389 |
| Unique reflections | 29233 | 29102 |
| GOF on *F*^2^ | 0.775 | 0.664 |
| *R*_1_, *wR*_2_ [*I*>2*σ*(*I*)] | 0.0594 0.1593 | 0.0411 0.1140 |
| *R*_1_, *wR*_2_ [all data] | 0.0958 0.1904 | 0.0550 0.1310 |
| *^a^R*_1_ =∑ \|\|*F*_0_\| − \|*F*_c_\|\|/∑\|*F*_o_\|. *^b^*w*R*_2_ = {∑ [*w*(*F*_0_^2^ − *F*_c_^2^)^2^]/∑*w*(*F*_0_^2^)^2^}^1/2^. | | |

**Table S2**. Crystallographic data for compounds **2**, **2a, 3, 3m** and **3a**.

|  | **2** | **2a** | **3** | **3m** | **3a** |
| --- | --- | --- | --- | --- | --- |
| Formula | C_135_H_95_Gd_4_NO_29_ | C_135_H_95_Gd_4_NO_29_ | C_135_H_95_Y_4_NO_29_ | C_135_H_95_Y_4_NO_29_ | C_135_H_95_Y_4_NO_29_ |
| *M*r (g·mol^-1^) | 2824.11 | 2824.11 | 2550.75 | 2550.75 | 2550.75 |
| Temperature | 293(2) K | 293(2) K | 293(2) K | 293(2) K | 293(2) K |
| Space group | *P*2_1_/*n* | *P*2_1_/*n* | *P*2_1_/*n* | *P*2_1_/*n* | *P*2_1_/*n* |
| Crystal system | Monoclinic | Monoclinic | Monoclinic | Monoclinic | Monoclinic |
| *a* (Å) | 19.1149(3) | 19.1968(4) | 19.09040(10) | 19.1796(7) | 19.17010(10) |
| *b* (Å) | 26.8237(5) | 27.0713(5) | 26.7042(2) | 26.7579(11) | 26.56360(10) |
| *c* (Å) | 24.8999(4) | 25.2042(4) | 24.9905(2) | 24.9832(8) | 24.88060(10) |
| *α* (*°*) | 90 | 90 | 90 | 90 | 90 |
| *β* (*°*) | 91.480(2) | 92.012(2) | 92.4440(10) | 92.183(3) | 92.3000(10) |
| γ (*°*) | 90 | 90 | 90 | 90 | 90 |
| *V* (Å^3^) | 12762.7(4) | 13090.1(4) | 12728.42(15) | 12812.2(8) | 12659.66(10) |
| *Z* | 4 | 4 | 4 | 4 | 4 |
| *F* (000) | 5600 | 5600 | 5200 | 5200 | 5200 |
| *Dc* (gcm^-3^) | 1.470 | 1.433 | 1.331 | 1.322 | 1.338 |
| *μ* (mm^-1^) | 2.124 | 2.071 | 2.966 | 1.866 | 2.982 |
| *R*_int_ | 0.0518 | 0.0496 | 0.0442 | 0.0728 | 0.0401 |
| limiting indices | -22<=h<=22  -31<=k<=31  -29<=l<=29 | -22<=h<=22  -32<=k<=29  -29<=l<=29 | -23<=h<=24  -32<=k<=30  -30<=l<=30 | -22<=h<=22  -31<=k<=31  -29<=l<=29 | -23<=h<=24  -33<=k<=32  -31<=l<=28 |
| Collected reflections | 110973 | 104695 | 96075 | 84119 | 95694 |
| Unique reflections | 22504 | 23027 | 25643 | 22595 | 25869 |
| GOF on *F*^2^ | 0.743 | 0.708 | 0.616 | 0.904 | 0.797 |
| *R*_1_, *wR*_2_ [*I*>2*σ*(*I*)] | 0.0591 0.1698 | 0.0472 0.1302 | 0.0574 0.1718 | 0.0752 0.2334 | 0.0469 0.1457 |
| *R*_1_, *wR*_2_ [all data] | 0.0883 0.1911 | 0.0652 0.1640 | 0.0611 0.1769 | 0.1300 0.2627 | 0.0513 0.1511 |
| *^a^R*_1_ =∑ \|\|*F*_0_\| − \|*F*_c_\|\|/∑\|*F*_o_\|. *^b^*w*R*_2_ = {∑ [*w*(*F*_0_^2^ − *F*_c_^2^)^2^]/∑*w*(*F*_0_^2^)^2^}^1/2^. | | | | | |

**Table S3**. Continuous Shape Measure (CShM) analyses of geometries for compound **1** by SHAPE 2.0 Software.

| Geometry | **Dy1** | **Dy2** | **Dy3** | **Dy4** |
| --- | --- | --- | --- | --- |
| Octagon (*D_8h_*) | 30.854 | 29.284 | 29.309 | 28.723 |
| Heptagonal pyramid (*C_7v_*) | 23.366 | 22.155 | 22.458 | 21.458 |
| Hexagonal bipyramid (*D_6h_*) | 16.502 | 14.561 | 15.135 | 14.707 |
| Cube (*O_h_*) | 11.476 | 13.671 | 13.634 | 13.540 |
| Square antiprism (*D_4d_*) | 3.355 | 3.436 | 3.781 | 3.080 |
| Triangular dodecahedron (*D_2d_*) | 0.689 | 1.385 | 1.215 | 1.236 |
| Johnson gyrobifastigium J26 (*D_2d_*) | 12.219 | 12.350 | 12.417 | 12.317 |
| Johnson elongated triangular bipyramid J14 (*D_3h_*) | 27.830 | 27.126 | 27.271 | 28.125 |
| Biaugmented trigonal prism J50 (*C_2v_*) | 2.916 | 3.053 | 3.322 | 2.556 |
| Biaugmented trigonal prism (*C_2v_*) | 2.009 | 2.402 | 2.752 | 1.741 |
| Snub diphenoid J84 (*D_2d_*) | 2.475 | 2.756 | 2.439 | 2.604 |
| Triakis tetrahedron (*T_d_*) | 12.249 | 14.362 | 14.364 | 14.222 |
| Elongated trigonal bipyramid (*D_3h_*) | 23.924 | 23.996 | 24.296 | 24.342 |

**Table S4**. Continuous Shape Measure (CShM) analyses of geometries for compound **1a** by SHAPE 2.0 Software.

| Geometry | **Dy1** | **Dy2** | **Dy3** | **Dy4** |
| --- | --- | --- | --- | --- |
| Octagon (*D_8h_*) | 31.621 | 28.891 | 29.701 | 30.259 |
| Heptagonal pyramid (*C_7v_*) | 23.537 | 21.858 | 22.308 | 21.829 |
| Hexagonal bipyramid (*D_6h_*) | 16.062 | 14.434 | 15.278 | 14.740 |
| Cube (*O_h_*) | 11.409 | 13.581 | 13.342 | 13.858 |
| Square antiprism (*D_4d_*) | 2.482 | 3.297 | 3.861 | 3.887 |
| Triangular dodecahedron (*D_2d_*) | 0.666 | 1.396 | 1.169 | 1.199 |
| Johnson gyrobifastigium J26 (*D_2d_*) | 12.700 | 12.201 | 12.634 | 12.119 |
| Johnson elongated triangular bipyramid J14 (*D_3h_*) | 27.688 | 26.954 | 27.342 | 28.465 |
| Biaugmented trigonal prism J50 (*C_2v_*) | 2.508 | 2.957 | 3.226 | 3.209 |
| Biaugmented trigonal prism (*C_2v_*) | 2.088 | 2.366 | 2.679 | 2.492 |
| Snub diphenoid J84 (*D_2d_*) | 2.303 | 2.708 | 2.591 | 2.569 |
| Triakis tetrahedron (*T_d_*) | 12.063 | 14.302 | 14.003 | 14.521 |
| Elongated trigonal bipyramid (*D_3h_*) | 23.751 | 23.843 | 24.376 | 24.902 |

**Table S5**. Continuous Shape Measure (CShM) analyses of geometries for compound **2** by SHAPE 2.0 Software.

| Geometry | **Gd1** | **Gd2** | **Gd3** | **Gd4** |
| --- | --- | --- | --- | --- |
| Octagon (*D_8h_*) | 29.294 | 28.306 | 28.974 | 29.294 |
| Heptagonal pyramid (*C_7v_*) | 22.227 | 21.208 | 21.901 | 22.227 |
| Hexagonal bipyramid (*D_6h_*) | 14.992 | 14.836 | 14.674 | 14.992 |
| Cube (*O_h_*) | 13.808 | 13.600 | 13.747 | 13.808 |
| Square antiprism (*D_4d_*) | 3.799 | 3.150 | 3.369 | 3.799 |
| Triangular dodecahedron (*D_2d_*) | 1.298 | 1.322 | 1.436 | 1.298 |
| Johnson gyrobifastigium J26 (*D_2d_*) | 12.313 | 12.450 | 12.440 | 12.313 |
| Johnson elongated triangular bipyramid J14 (*D_3h_*) | 27.433 | 27.901 | 26.797 | 27.433 |
| Biaugmented trigonal prism J50 (*C_2v_*) | 3.267 | 2.592 | 3.054 | 3.267 |
| Biaugmented trigonal prism (*C_2v_*) | 2.706 | 1.753 | 2.452 | 2.706 |
| Snub diphenoid J84 (*D_2d_*) | 2.530 | 2.672 | 2.756 | 2.530 |
| Triakis tetrahedron (*T_d_*) | 14.527 | 14.265 | 14.429 | 14.527 |
| Elongated trigonal bipyramid (*D_3h_*) | 24.465 | 24.068 | 23.684 | 24.465 |

**Table S6**. Continuous Shape Measure (CShM) analyses of geometries for compound **2a** by SHAPE 2.0 Software.

| Geometry | **Gd1** | **Gd2** | **Gd3** | **Gd4** |
| --- | --- | --- | --- | --- |
| Octagon (*D_8h_*) | 29.795 | 31.546 | 29.550 | 28.953 |
| Heptagonal pyramid (*C_7v_*) | 21.688 | 23.796 | 21.986 | 21.514 |
| Hexagonal bipyramid (*D_6h_*) | 14.727 | 15.796 | 15.061 | 14.239 |
| Cube (*O_h_*) | 14.119 | 11.564 | 13.662 | 13.542 |
| Square antiprism (*D_4d_*) | 3.928 | 2.544 | 3.975 | 3.384 |
| Triangular dodecahedron (*D_2d_*) | 1.324 | 0.711 | 1.309 | 1.412 |
| Johnson gyrobifastigium J26 (*D_2d_*) | 12.084 | 12.453 | 12.580 | 12.118 |
| Johnson elongated triangular bipyramid J14 (*D_3h_*) | 28.045 | 27.944 | 27.395 | 27.417 |
| Biaugmented trigonal prism J50 (*C_2v_*) | 3.140 | 2.464 | 3.335 | 3.147 |
| Biaugmented trigonal prism (*C_2v_*) | 2.386 | 1.988 | 2.752 | 2.549 |
| Snub diphenoid J84 (*D_2d_*) | 2.633 | 2.433 | 2.679 | 2.721 |
| Triakis tetrahedron (*T_d_*) | 14.769 | 12.271 | 14.307 | 14.216 |
| Elongated trigonal bipyramid (*D_3h_*) | 24.449 | 23.926 | 24.504 | 24.365 |

**Table S7**. Continuous Shape Measure (CShM) analyses of geometries for compound **3** by SHAPE 2.0 Software.

| Geometry | **Y1** | **Y2** | **Y3** | **Y4** |
| --- | --- | --- | --- | --- |
| Octagon (*D_8h_*) | 29.207 | 30.969 | 29.191 | 29.004 |
| Heptagonal pyramid (*C_7v_*) | 21.666 | 23.793 | 22.488 | 21.908 |
| Hexagonal bipyramid (*D_6h_*) | 14.677 | 16.548 | 15.338 | 14.538 |
| Cube (*O_h_*) | 13.555 | 11.332 | 13.282 | 13.762 |
| Square antiprism (*D_4d_*) | 3.225 | 3.290 | 3.515 | 3.381 |
| Triangular dodecahedron (*D_2d_*) | 1.165 | 0.639 | 1.110 | 1.266 |
| Johnson gyrobifastigium J26 (*D_2d_*) | 12.335 | 12.294 | 12.675 | 12.104 |
| Johnson elongated triangular bipyramid J14 (*D_3h_*) | 28.182 | 27.946 | 27.546 | 27.748 |
| Biaugmented trigonal prism J50 (*C_2v_*) | 2.723 | 2.735 | 3.151 | 3.171 |
| Biaugmented trigonal prism (*C_2v_*) | 1.984 | 2.138 | 2.613 | 2.651 |
| Snub diphenoid J84 (*D_2d_*) | 2.575 | 2.335 | 2.594 | 2.502 |
| Triakis tetrahedron (*T_d_*) | 14.213 | 12.038 | 13.946 | 14.438 |
| Elongated trigonal bipyramid (*D_3h_*) | 24.517 | 23.990 | 24.450 | 24.756 |

**Table S8**. Continuous Shape Measure (CShM) analyses of geometries for compound **3m** by SHAPE 2.0 Software.

| Geometry | **Y1** | **Y2** | **Y3** | **Y4** |
| --- | --- | --- | --- | --- |
| Octagon (*D_8h_*) | 29.018 | 31.647 | 29.775 | 29.272 |
| Heptagonal pyramid (*C_7v_*) | 21.901 | 23.872 | 21.656 | 22.266 |
| Hexagonal bipyramid (*D_6h_*) | 14.598 | 15.975 | 14.534 | 15.154 |
| Cube (*O_h_*) | 13.709 | 11.563 | 13.765 | 13.483 |
| Square antiprism (*D_4d_*) | 3.335 | 2.750 | 3.547 | 3.729 |
| Triangular dodecahedron (*D_2d_*) | 1.250 | 0.721 | 1.238 | 1.175 |
| Johnson gyrobifastigium J26 (*D_2d_*) | 12.130 | 12.319 | 12.179 | 12.594 |
| Johnson elongated triangular bipyramid J14 (*D_3h_*) | 27.664 | 27.681 | 28.246 | 27.552 |
| Biaugmented trigonal prism J50 (*C_2v_*) | 3.019 | 2.347 | 3.013 | 3.205 |
| Biaugmented trigonal prism (*C_2v_*) | 2.467 | 1.894 | 2.303 | 2.559 |
| Snub diphenoid J84 (*D_2d_*) | 2.537 | 2.375 | 2.579 | 2.608 |
| Triakis tetrahedron (*T_d_*) | 14.405 | 12.252 | 14.329 | 14.125 |
| Elongated trigonal bipyramid (*D_3h_*) | 24.674 | 23.947 | 25.152 | 24.617 |

**Table S9**. Continuous Shape Measure (CShM) analyses of geometries for compound **3a** by SHAPE 2.0 Software.

| Geometry | **Y1** | **Y2** | **Y3** | **Y4** |
| --- | --- | --- | --- | --- |
| Octagon (*D_8h_*) | 30.342 | 31.253 | 29.155 | 28.651 |
| Heptagonal pyramid (*C_7v_*) | 21.877 | 23.552 | 22.478 | 21.548 |
| Hexagonal bipyramid (*D_6h_*) | 14.720 | 16.162 | 15.485 | 14.485 |
| Cube (*O_h_*) | 13.818 | 11.445 | 13.222 | 13.475 |
| Square antiprism (*D_4d_*) | 3.771 | 2.531 | 3.539 | 3.105 |
| Triangular dodecahedron (*D_2d_*) | 1.160 | 0.636 | 1.120 | 1.263 |
| Johnson gyrobifastigium J26 (*D_2d_*) | 12.214 | 12.512 | 12.698 | 12.219 |
| Johnson elongated triangular bipyramid J14 (*D_3h_*) | 28.338 | 27.856 | 27.413 | 27.827 |
| Biaugmented trigonal prism J50 (*C_2v_*) | 3.196 | 2.331 | 3.007 | 3.019 |
| Biaugmented trigonal prism (*C_2v_*) | 2.538 | 1.906 | 2.416 | 2.470 |
| Snub diphenoid J84 (*D_2d_*) | 2.538 | 2.237 | 2.581 | 2.584 |
| Triakis tetrahedron (*T_d_*) | 14.497 | 12.117 | 13.863 | 14.154 |
| Elongated trigonal bipyramid (*D_3h_*) | 25.033 | 23.906 | 24.409 | 24.726 |

**Table S10**. Selected bond lengths (Å) and angles (^o^) for compound **1** at 180(2) K.

| Compound **1** | | | |
| --- | --- | --- | --- |
| Dy(1)-O(2) | 2.300(6) | Dy(3)-O(25) | 2.290(5) |
| Dy(1)-O(4) | 2.341(6) | Dy(3)-O(12) | 2.307(5) |
| Dy(1)-O(6) | 2.359(5) | Dy(3)-O(15) | 2.332(6) |
| Dy(1)-O(24) | 2.357(5) | Dy(3)-O(20) | 2.338(6) |
| Dy(1)-O(19) | 2.357(6) | Dy(3)-O(26) | 2.361(5) |
| Dy(1)-O(23) | 2.371(5) | Dy(3)-O(23) | 2.376(5) |
| Dy(1)-O(25) | 2.429(5) | Dy(3)-O(14) | 2.409(5) |
| Dy(1)-O(3) | 2.448(6) | Dy(3)-O(13) | 2.475(6) |
| Dy(2)-O(24) | 2.318(5) | Dy(4)-O(24) | 2.317(5) |
| Dy(2)-O(26) | 2.322(5) | Dy(4)-O(16) | 2.359(6) |
| Dy(2)-O(11) | 2.323(5) | Dy(4)-O(22) | 2.367(6) |
| Dy(2)-O(23) | 2.348(5) | Dy(4)-O(1) | 2.366(6) |
| Dy(2)-O(21) | 2.354(6) | Dy(4)-O(25) | 2.379(5) |
| Dy(2)-O(7) | 2.381(6) | Dy(4)-O(26) | 2.388(5) |
| Dy(2)-O(8) | 2.453(6) | Dy(4)-O(17) | 2.422(6) |
| Dy(2)-O(10) | 2.471(5) | Dy(4)-O(18) | 2.458(6) |
|  |  |  |  |
| O(2)-Dy(1)-O(4) | 75.7(2) | C(59)-C(46)-C(60) | 119.3(7) |
| O(2)-Dy(1)-O(6) | 146.7(2) | C(46)-C(47)-C(48) | 123.5(8) |
| O(4)-Dy(1)-O(6) | 74.1(2) | C(46)-C(47)-C(52) | 119.1(8) |
| O(2)-Dy(1)-O(24) | 96.6(2) | C(48)-C(47)-C(52) | 117.4(8) |
| O(4)-Dy(1)-O(24) | 77.66(19) | C(49)-C(48)-C(47) | 122.0(9) |
| O(6)-Dy(1)-O(24) | 90.4(2) | C(48)-C(49)-C(50) | 118.8(10) |
| O(2)-Dy(1)-O(19) | 101.9(2) | C(51)-C(50)-C(49) | 122.0(9) |
| O(4)-Dy(1)-O(19) | 137.1(2) | C(50)-C(51)-C(52) | 121.2(9) |
| O(6)-Dy(1)-O(19) | 90.9(2) | C(53)-C(52)-C(51) | 122.9(9) |
| O(24)-Dy(1)-O(19) | 143.70(19) | C(53)-C(52)-C(47) | 118.5(8) |
| O(2)-Dy(1)-O(23) | 139.12(19) | C(51)-C(52)-C(47) | 118.5(9) |
| O(4)-Dy(1)-O(23) | 134.41(18) | C(52)-C(53)-C(54) | 123.4(9) |
| O(6)-Dy(1)-O(23) | 73.80(18) | C(53)-C(54)-C(59) | 118.9(9) |
| O(24)-Dy(1)-O(23) | 71.07(17) | C(53)-C(54)-C(55) | 123.1(9) |
| O(19)-Dy(1)-O(23) | 74.49(19) | C(59)-C(54)-C(55) | 118.0(8) |
| O(2)-Dy(1)-O(25) | 70.73(19) | C(56)-C(55)-C(54) | 121.0(9) |
| O(4)-Dy(1)-O(25) | 130.49(19) | C(55)-C(56)-C(57) | 119.9(10) |
| O(6)-Dy(1)-O(25) | 141.57(19) | C(58)-C(57)-C(56) | 121.3(10) |
| O(24)-Dy(1)-O(25) | 71.35(17) | C(57)-C(58)-C(59) | 121.5(9) |
| O(19)-Dy(1)-O(25) | 85.7(2) | C(58)-C(59)-C(46) | 122.9(8) |
| O(23)-Dy(1)-O(25) | 68.40(18) | C(58)-C(59)-C(54) | 118.3(8) |
| O(2)-Dy(1)-O(3) | 77.2(2) | C(46)-C(59)-C(54) | 118.9(8) |
| O(4)-Dy(1)-O(3) | 67.7(2) | O(12)-C(60)-O(11) | 126.1(7) |
| O(6)-Dy(1)-O(3) | 78.7(2) | O(12)-C(60)-C(46) | 117.2(7) |
| O(24)-Dy(1)-O(3) | 145.3(2) | O(11)-C(60)-C(46) | 116.7(7) |
| O(19)-Dy(1)-O(3) | 70.0(2) | C(62)-C(61)-C(74) | 120.8(10) |
| O(23)-Dy(1)-O(3) | 134.2(2) | C(62)-C(61)-C(75) | 120.9(10) |
| O(25)-Dy(1)-O(3) | 134.4(2) | C(74)-C(61)-C(75) | 118.2(10) |
| O(24)-Dy(2)-O(26) | 72.37(17) | C(61)-C(62)-C(63) | 122.4(9) |
| O(24)-Dy(2)-O(11) | 147.66(19) | C(61)-C(62)-C(67) | 119.8(9) |
| O(26)-Dy(2)-O(11) | 82.85(19) | C(63)-C(62)-C(67) | 117.8(9) |
| O(24)-Dy(2)-O(23) | 72.15(17) | C(64)-C(63)-C(62) | 121.6(10) |
| O(25)-Dy(4)-O(17) | 134.69(18) | O(17)-C(105)-O(18) | 122.5(8) |
| O(26)-Dy(4)-O(17) | 133.84(19) | O(17)-C(105)-C(91) | 117.2(8) |
| O(24)-Dy(4)-O(18) | 133.87(18) | O(18)-C(105)-C(91) | 120.3(8) |
| O(16)-Dy(4)-O(18) | 79.4(2) | C(119)-C(106)-C(107) | 121.4(9) |
| O(22)-Dy(4)-O(18) | 76.9(2) | C(119)-C(106)-C(120) | 118.3(9) |
| O(1)-Dy(4)-O(18) | 74.8(2) | C(107)-C(106)-C(120) | 120.3(9) |
| O(25)-Dy(4)-O(18) | 138.2(2) | C(106)-C(107)-C(112) | 119.4(10) |
| O(26)-Dy(4)-O(18) | 141.31(19) | C(106)-C(107)-C(108) | 122.5(9) |
| O(17)-Dy(4)-O(18) | 53.80(18) | C(112)-C(107)-C(108) | 118.1(10) |
| C(2)-C(1)-C(14) | 120.9(11) | C(109)-C(108)-C(107) | 121.8(10) |
| C(2)-C(1)-C(15) | 121.2(11) | C(108)-C(109)-C(110) | 120.5(11) |
| C(14)-C(1)-C(15) | 117.6(11) | C(111)-C(110)-C(109) | 119.0(11) |
| C(3)-C(2)-C(1) | 124.2(11) | C(110)-C(111)-C(112) | 123.5(10) |
| C(3)-C(2)-C(7) | 115.8(12) | C(113)-C(112)-C(107) | 119.2(10) |
| C(1)-C(2)-C(7) | 119.7(12) | C(113)-C(112)-C(111) | 123.8(10) |
| C(2)-C(3)-C(4) | 132.4(11) | C(107)-C(112)-C(111) | 117.0(10) |
| C(3)-C(4)-C(5) | 108.4(12) | C(114)-C(113)-C(112) | 122.8(10) |
| C(6)-C(5)-C(4) | 123.1(13) | C(113)-C(114)-C(115) | 123.1(10) |
| C(5)-C(6)-C(7) | 121.7(12) | C(113)-C(114)-C(119) | 119.0(10) |
| C(8)-C(7)-C(6) | 122.8(12) | C(115)-C(114)-C(119) | 118.0(11) |
| C(8)-C(7)-C(2) | 118.8(13) | C(116)-C(115)-C(114) | 121.8(11) |
| C(6)-C(7)-C(2) | 118.3(13) | C(115)-C(116)-C(117) | 121.7(11) |
| C(7)-C(8)-C(9) | 125.9(12) | C(118)-C(117)-C(116) | 119.5(11) |
| C(8)-C(9)-C(10) | 125.2(12) | C(117)-C(118)-C(119) | 121.0(10) |
| C(8)-C(9)-C(14) | 117.2(12) | C(106)-C(119)-C(118) | 123.8(9) |
| C(10)-C(9)-C(14) | 117.5(13) | C(106)-C(119)-C(114) | 118.2(10) |
| C(11)-C(10)-C(9) | 122.2(13) | C(118)-C(119)-C(114) | 118.0(10) |
| C(10)-C(11)-C(12) | 121.4(14) | O(20)-C(120)-O(19) | 125.5(8) |
| C(13)-C(12)-C(11) | 121.4(15) | O(20)-C(120)-C(106) | 116.3(8) |
| C(12)-C(13)-C(14) | 119.6(12) | O(19)-C(120)-C(106) | 118.1(8) |

**Table S11**. Selected bond lengths (Å) and angles (^o^) for compound **1a** at 120(2) K.

| Compound **1a** | | | |
| --- | --- | --- | --- |
| Dy(1)-O(2) | 2.278(3) | Dy(3)-O(25) | 2.273(3) |
| Dy(1)-O(19) | 2.326(4) | Dy(3)-O(12) | 2.305(4) |
| Dy(1)-O(6) | 2.342(4) | Dy(3)-O(20) | 2.340(4) |
| Dy(1)-O(24) | 2.353(3) | Dy(3)-O(26) | 2.340(3) |
| Dy(1)-O(4) | 2.381(4) | Dy(3)-O(15) | 2.378(4) |
| Dy(1)-O(23) | 2.392(3) | Dy(3)-O(23) | 2.384(3) |
| Dy(1)-O(3) | 2.434(4) | Dy(3)-O(14) | 2.390(3) |
| Dy(1)-O(25) | 2.432(3) | Dy(3)-O(13) | 2.445(4) |
| Dy(2)-O(24) | 2.310(3) | Dy(4)-O(24) | 2.315(3) |
| Dy(2)-O(26) | 2.327(3) | Dy(4)-O(16) | 2.326(4) |
| Dy(2)-O(11) | 2.336(4) | Dy(4)-O(25) | 2.370(3) |
| Dy(2)-O(23) | 2.339(3) | Dy(4)-O(22) | 2.381(4) |
| Dy(2)-O(21) | 2.340(4) | Dy(4)-O(26) | 2.398(3) |
| Dy(2)-O(7) | 2.385(4) | Dy(4)-O(1) | 2.419(4) |
| Dy(2)-O(8) | 2.458(4) | Dy(4)-O(17) | 2.420(4) |
| Dy(2)-O(10) | 2.495(4) | Dy(4)-O(18) | 2.455(4) |
|  |  |  |  |
| O(2)-Dy(1)-O(19) | 97.35(13) | O(7)-C(45)-C(31) | 116.2(5) |
| O(2)-Dy(1)-O(6) | 148.48(13) | C(47)-C(46)-C(59) | 120.8(5) |
| O(19)-Dy(1)-O(6) | 95.34(15) | C(47)-C(46)-C(60) | 119.9(5) |
| O(2)-Dy(1)-O(24) | 98.71(12) | C(59)-C(46)-C(60) | 119.2(5) |
| O(19)-Dy(1)-O(24) | 143.00(13) | C(46)-C(47)-C(52) | 119.6(6) |
| O(6)-Dy(1)-O(24) | 87.99(13) | C(46)-C(47)-C(48) | 122.2(5) |
| O(2)-Dy(1)-O(4) | 75.07(12) | C(52)-C(47)-C(48) | 118.2(5) |
| O(19)-Dy(1)-O(4) | 139.30(14) | C(49)-C(48)-C(47) | 120.5(6) |
| O(6)-Dy(1)-O(4) | 76.49(13) | C(48)-C(49)-C(50) | 122.2(8) |
| O(24)-Dy(1)-O(4) | 77.30(13) | C(51)-C(50)-C(49) | 119.6(8) |
| O(2)-Dy(1)-O(23) | 137.32(12) | C(50)-C(51)-C(52) | 121.2(7) |
| O(19)-Dy(1)-O(23) | 74.65(13) | C(53)-C(52)-C(47) | 119.5(6) |
| O(6)-Dy(1)-O(23) | 73.98(13) | C(53)-C(52)-C(51) | 122.2(6) |
| O(24)-Dy(1)-O(23) | 70.93(12) | C(47)-C(52)-C(51) | 118.3(6) |
| O(4)-Dy(1)-O(23) | 136.83(12) | C(54)-C(53)-C(52) | 121.9(6) |
| O(2)-Dy(1)-O(3) | 80.10(14) | C(53)-C(54)-C(59) | 119.4(6) |
| O(19)-Dy(1)-O(3) | 69.69(15) | C(53)-C(54)-C(55) | 121.2(6) |
| O(6)-Dy(1)-O(3) | 77.49(14) | C(59)-C(54)-C(55) | 119.4(6) |
| O(24)-Dy(1)-O(3) | 146.09(14) | C(56)-C(55)-C(54) | 120.0(6) |
| O(4)-Dy(1)-O(3) | 69.61(15) | C(55)-C(56)-C(57) | 122.7(6) |
| O(23)-Dy(1)-O(3) | 131.33(14) | C(58)-C(57)-C(56) | 117.8(7) |
| O(2)-Dy(1)-O(25) | 69.78(12) | C(57)-C(58)-C(59) | 121.6(6) |
| O(19)-Dy(1)-O(25) | 83.33(13) | C(46)-C(59)-C(54) | 118.9(6) |
| O(6)-Dy(1)-O(25) | 140.58(13) | C(46)-C(59)-C(58) | 122.6(5) |
| O(24)-Dy(1)-O(25) | 71.47(11) | C(54)-C(59)-C(58) | 118.5(6) |
| O(4)-Dy(1)-O(25) | 127.82(12) | O(11)-C(60)-O(12) | 126.3(5) |
| O(23)-Dy(1)-O(25) | 67.68(12) | O(11)-C(60)-C(46) | 117.0(5) |
| O(3)-Dy(1)-O(25) | 136.34(13) | O(12)-C(60)-C(46) | 116.7(5) |
| O(24)-Dy(2)-O(26) | 72.21(12) | C(62)-C(61)-C(74) | 121.2(5) |
| O(24)-Dy(2)-O(11) | 148.21(12) | C(62)-C(61)-C(75) | 120.0(5) |
| O(26)-Dy(2)-O(11) | 83.62(13) | C(74)-C(61)-C(75) | 118.8(5) |
| O(24)-Dy(2)-O(23) | 72.63(12) | C(61)-C(62)-C(67) | 119.5(5) |
| O(1)-Dy(4)-O(17) | 74.71(12) | C(91)-C(104)-C(99) | 119.0(6) |
| O(24)-Dy(4)-O(18) | 132.58(12) | C(103)-C(104)-C(99) | 118.5(6) |
| O(16)-Dy(4)-O(18) | 80.35(13) | O(17)-C(105)-O(18) | 122.0(5) |
| O(25)-Dy(4)-O(18) | 140.41(12) | O(17)-C(105)-C(91) | 117.8(5) |
| O(22)-Dy(4)-O(18) | 75.88(14) | O(18)-C(105)-C(91) | 120.1(5) |
| O(26)-Dy(4)-O(18) | 140.56(13) | C(119)-C(106)-C(107) | 121.1(5) |
| O(1)-Dy(4)-O(18) | 76.02(13) | C(119)-C(106)-C(120) | 119.5(5) |
| O(17)-Dy(4)-O(18) | 53.64(12) | C(107)-C(106)-C(120) | 119.3(5) |
| C(15)-C(1)-C(14) | 117.4(4) | C(106)-C(107)-C(108) | 122.7(5) |
| C(15)-C(1)-C(2) | 108.2(4) | C(106)-C(107)-C(112) | 119.3(6) |
| C(14)-C(1)-C(2) | 107.1(4) | C(108)-C(107)-C(112) | 118.0(6) |
| C(15)-C(1)-C(8)#1 | 106.3(4) | C(109)-C(108)-C(107) | 121.3(7) |
| C(14)-C(1)-C(8)#1 | 108.0(4) | C(108)-C(109)-C(110) | 121.0(8) |
| C(2)-C(1)-C(8)#1 | 109.7(4) | C(111)-C(110)-C(109) | 120.1(7) |
| C(3)-C(2)-C(7) | 118.3(5) | C(110)-C(111)-C(112) | 121.8(7) |
| C(3)-C(2)-C(1) | 125.2(4) | C(113)-C(112)-C(107) | 119.5(7) |
| C(7)-C(2)-C(1) | 116.5(4) | C(113)-C(112)-C(111) | 122.8(7) |
| C(4)-C(3)-C(2) | 121.2(5) | C(107)-C(112)-C(111) | 117.7(7) |
| C(5)-C(4)-C(3) | 120.0(5) | C(114)-C(113)-C(112) | 121.7(6) |
| C(4)-C(5)-C(6) | 119.4(5) | C(113)-C(114)-C(115) | 122.4(7) |
| C(7)-C(6)-C(5) | 120.7(5) | C(113)-C(114)-C(119) | 120.5(7) |
| C(6)-C(7)-C(2) | 120.4(5) | C(115)-C(114)-C(119) | 117.1(8) |
| C(6)-C(7)-C(8) | 121.3(5) | C(116)-C(115)-C(114) | 122.6(8) |
| C(2)-C(7)-C(8) | 118.2(5) | C(115)-C(116)-C(117) | 120.1(8) |
| C(7)-C(8)-C(9) | 108.8(4) | C(118)-C(117)-C(116) | 120.0(9) |
| C(7)-C(8)-C(1)#1 | 114.6(4) | C(117)-C(118)-C(119) | 121.1(7) |
| C(9)-C(8)-C(1)#1 | 113.2(4) | C(106)-C(119)-C(118) | 123.0(6) |
| C(10)-C(9)-C(14) | 120.5(5) | C(106)-C(119)-C(114) | 117.9(6) |
| C(10)-C(9)-C(8) | 120.9(5) | C(118)-C(119)-C(114) | 119.1(6) |
| C(14)-C(9)-C(8) | 118.6(4) | O(20)-C(120)-O(19) | 124.8(5) |
| Symmetry codes: #1 -x,-y+1,-z+1 | | | |

**Table S12**. Selected bond lengths (Å) and angles (^o^) for compound **2** at 293(2) K.

| Compound **2** | | | |
| --- | --- | --- | --- |
| Gd(1)-O(18) | 2.327(7) | Gd(3)-O(3) | 2.338(5) |
| Gd(1)-O(12) | 2.368(6) | Gd(3)-O(10) | 2.353(5) |
| Gd(1)-O(16) | 2.383(6) | Gd(3)-O(2) | 2.362(6) |
| Gd(1)-O(3) | 2.386(5) | Gd(3)-O(23) | 2.364(6) |
| Gd(1)-O(8) | 2.389(6) | Gd(3)-O(4) | 2.385(5) |
| Gd(1)-O(4) | 2.393(5) | Gd(3)-O(11) | 2.406(6) |
| Gd(1)-O(1) | 2.446(5) | Gd(3)-O(14) | 2.485(6) |
| Gd(1)-O(26) | 2.469(7) | Gd(3)-O(13) | 2.499(6) |
| Gd(2)-O(3) | 2.344(6) | Gd(4)-O(1) | 2.318(5) |
| Gd(2)-O(25) | 2.372(7) | Gd(4)-O(9) | 2.339(6) |
| Gd(2)-O(20) | 2.471(6) | Gd(4)-O(24) | 2.356(7) |
| Gd(2)-O(19) | 2.389(8) | Gd(4)-O(7) | 2.354(6) |
| Gd(2)-O(22) | 2.397(7) | Gd(4)-O(4) | 2.397(5) |
| Gd(2)-O(2) | 2.410(6) | Gd(4)-O(2) | 2.393(5) |
| Gd(2)-O(1) | 2.415(5) | Gd(4)-O(5) | 2.425(5) |
| Gd(2)-O(21) | 2.449(6) | Gd(4)-O(6) | 2.484(6) |
|  |  |  |  |
| C(106)-O(20)-Gd(2) | 91.7(5) | C(108)-C(107)-C(106) | 121.1(10) |
| O(24)-C(1)-O(25) | 124.2(9) | C(109)-C(108)-C(113) | 119.7(12) |
| O(24)-C(1)-C(2) | 110.2(10) | C(109)-C(108)-C(107) | 120.0(9) |
| O(25)-C(1)-C(2) | 124.5(10) | C(113)-C(108)-C(107) | 120.2(11) |
| C(15)-C(2)-C(3) | 122.4(11) | C(108)-C(109)-C(110) | 120.4(12) |
| C(15)-C(2)-C(1) | 113.8(11) | C(109)-C(110)-C(111) | 119.6(14) |
| C(3)-C(2)-C(1) | 123.8(12) | C(112)-C(111)-C(110) | 120.8(14) |
| C(4)-C(3)-C(8) | 120 | C(111)-C(112)-C(113) | 119.5(12) |
| C(4)-C(3)-C(2) | 124.2(10) | C(114)-C(113)-C(112) | 121.7(11) |
| C(8)-C(3)-C(2) | 115.8(10) | C(114)-C(113)-C(108) | 118.5(11) |
| C(5)-C(4)-C(3) | 120 | C(112)-C(113)-C(108) | 119.8(12) |
| C(4)-C(5)-C(6) | 120 | C(113)-C(114)-C(115) | 123.4(11) |
| C(5)-C(6)-C(7) | 120 | C(114)-C(115)-C(116) | 122.1(11) |
| C(8)-C(7)-C(6) | 120 | C(114)-C(115)-C(120) | 117.7(12) |
| C(9)-C(8)-C(7) | 115.2(11) | C(116)-C(115)-C(120) | 120.2(13) |
| C(9)-C(8)-C(3) | 124.7(11) | C(117)-C(116)-C(115) | 120.3(14) |
| C(7)-C(8)-C(3) | 120 | C(116)-C(117)-C(118) | 120.7(15) |
| C(8)-C(9)-C(10) | 115.1(13) | C(119)-C(118)-C(117) | 117.8(14) |
| C(11)-C(10)-C(15) | 120 | C(118)-C(119)-C(120) | 123.0(13) |
| C(11)-C(10)-C(9) | 116.8(11) | C(107)-C(120)-C(115) | 121.2(13) |
| C(15)-C(10)-C(9) | 123.0(11) | C(107)-C(120)-C(119) | 121.0(9) |
| C(10)-C(11)-C(12) | 120 | C(115)-C(120)-C(119) | 117.7(13) |
| C(13)-C(12)-C(11) | 120 | O(23)-C(121)-O(22) | 126.6(9) |
| C(12)-C(13)-C(14) | 120 | O(23)-C(121)-C(122) | 115.1(9) |
| C(15)-C(14)-C(13) | 120 | O(22)-C(121)-C(122) | 118.2(9) |
| C(2)-C(15)-C(14) | 121.1(10) | C(135)-C(122)-C(123) | 120.5(9) |
| C(2)-C(15)-C(10) | 118.9(10) | C(135)-C(122)-C(121) | 120.7(9) |
| C(14)-C(15)-C(10) | 120 | C(123)-C(122)-C(121) | 118.8(8) |
| O(6)-C(16)-O(5) | 121.2(8) | C(124)-C(123)-C(122) | 123.3(9) |
| O(6)-C(16)-C(17) | 119.9(8) | C(124)-C(123)-C(128) | 117.7(10) |
| O(5)-C(16)-C(17) | 118.8(7) | C(122)-C(123)-C(128) | 119.0(10) |
| C(30)-C(17)-C(18) | 119.7(8) | C(125)-C(124)-C(123) | 121.3(10) |
| C(30)-C(17)-C(16) | 123.4(8) | C(124)-C(125)-C(126) | 120.2(12) |
| C(18)-C(17)-C(16) | 116.9(7) | C(127)-C(126)-C(125) | 120.6(12) |
| C(19)-C(18)-C(17) | 122.6(8) | C(126)-C(127)-C(128) | 121.0(12) |
| C(19)-C(18)-C(23) | 118.4(9) | C(129)-C(128)-C(127) | 121.7(11) |
| C(17)-C(18)-C(23) | 119.0(8) | C(129)-C(128)-C(123) | 119.2(11) |
| C(20)-C(19)-C(18) | 121.3(10) | C(127)-C(128)-C(123) | 119.1(12) |
| C(19)-C(20)-C(21) | 120.2(11) | C(130)-C(129)-C(128) | 123.5(11) |
| C(22)-C(21)-C(20) | 120.2(10) | C(129)-C(130)-C(131) | 123.2(11) |
| C(21)-C(22)-C(23) | 121.7(11) | C(129)-C(130)-C(135) | 118.9(10) |
| C(22)-C(23)-C(24) | 123.4(9) | C(131)-C(130)-C(135) | 117.9(11) |
| C(22)-C(23)-C(18) | 118.1(10) | C(132)-C(131)-C(130) | 123.1(13) |
| C(24)-C(23)-C(18) | 118.5(9) | C(131)-C(132)-C(133) | 119.5(12) |
| C(25)-C(24)-C(23) | 122.5(9) | C(134)-C(133)-C(132) | 120.7(12) |
| C(24)-C(25)-C(30) | 119.8(9) | C(133)-C(134)-C(135) | 121.6(11) |
| C(24)-C(25)-C(26) | 121.2(10) | C(122)-C(135)-C(130) | 118.7(10) |
| C(30)-C(25)-C(26) | 119.0(10) | C(122)-C(135)-C(134) | 123.8(9) |
| C(27)-C(26)-C(25) | 119.2(11) | C(130)-C(135)-C(134) | 117.1(9) |
| C(26)-C(27)-C(28) | 122.8(11) | O(18)-Gd(1)-O(12) | 146.7(2) |
| C(29)-C(28)-C(27) | 119.4(12) | O(18)-Gd(1)-O(16) | 75.0(2) |
| C(28)-C(29)-C(30) | 120.9(11) | O(12)-Gd(1)-O(16) | 74.1(2) |
| C(17)-C(30)-C(29) | 121.0(9) | O(18)-Gd(1)-O(3) | 96.5(2) |
| C(17)-C(30)-C(25) | 120.2(9) | O(12)-Gd(1)-O(3) | 89.0(2) |
| C(29)-C(30)-C(25) | 118.7(9) | O(16)-Gd(1)-O(3) | 77.9(2) |
| O(7)-C(31)-O(8) | 125.4(8) | O(18)-Gd(1)-O(8) | 103.7(3) |
| O(7)-C(31)-C(32) | 116.5(8) | O(12)-Gd(1)-O(8) | 90.6(2) |
| O(8)-C(31)-C(32) | 118.2(8) | O(16)-Gd(1)-O(8) | 136.9(2) |
| C(33)-C(32)-C(45) | 120.8(9) | O(3)-Gd(1)-O(8) | 143.1(2) |
| C(33)-C(32)-C(31) | 119.9(9) | O(18)-Gd(1)-O(4) | 139.2(2) |
| C(45)-C(32)-C(31) | 119.3(9) | O(12)-Gd(1)-O(4) | 73.3(2) |
| C(32)-C(33)-C(34) | 124.4(9) | O(16)-Gd(1)-O(4) | 134.3(2) |
| C(32)-C(33)-C(38) | 117.8(11) | O(3)-Gd(1)-O(4) | 70.38(18) |
| C(34)-C(33)-C(38) | 117.8(11) | O(8)-Gd(1)-O(4) | 74.2(2) |
| C(35)-C(34)-C(33) | 122.8(13) | O(18)-Gd(1)-O(1) | 70.8(2) |
| C(34)-C(35)-C(36) | 119.9(16) | O(12)-Gd(1)-O(1) | 140.9(2) |
| C(37)-C(36)-C(35) | 119.2(15) | O(16)-Gd(1)-O(1) | 130.0(2) |
| C(36)-C(37)-C(38) | 124.6(15) | O(3)-Gd(1)-O(1) | 71.03(18) |
| C(37)-C(38)-C(39) | 126.1(14) | O(8)-Gd(1)-O(1) | 86.8(2) |
| C(37)-C(38)-C(33) | 115.6(14) | O(4)-Gd(1)-O(1) | 68.44(19) |
| C(39)-C(38)-C(33) | 118.3(12) | O(18)-Gd(1)-O(26) | 78.2(3) |
| C(38)-C(39)-C(40) | 123.9(11) | O(12)-Gd(1)-O(26) | 79.4(3) |
| C(39)-C(40)-C(45) | 119.0(13) | O(16)-Gd(1)-O(26) | 68.8(3) |

**Table S13**. Selected bond lengths (Å) and angles (^o^) for compound **2a** at 293(2) K.

| Compound **2a** | | | |
| --- | --- | --- | --- |
| Gd(1)-O(25) | 2.349(5) | Gd(3)-O(4) | 2.306(4) |
| Gd(1)-O(2) | 2.350(4) | Gd(3)-O(14) | 2.339(5) |
| Gd(1)-O(4) | 2.402(4) | Gd(3)-O(18) | 2.375(5) |
| Gd(1)-O(9) | 2.409(5) | Gd(3)-O(3) | 2.379(5) |
| Gd(1)-O(3) | 2.434(4) | Gd(3)-O(1) | 2.405(4) |
| Gd(1)-O(5) | 2.451(5) | Gd(3)-O(24) | 2.405(5) |
| Gd(1)-O(8) | 2.457(5) | Gd(3)-O(17) | 2.436(5) |
| Gd(1)-O(7) | 2.496(5) | Gd(3)-O(16) | 2.481(5) |
| Gd(2)-O(6) | 2.340(5) | Gd(4)-O(2) | 2.344(4) |
| Gd(2)-O(19) | 2.360(5) | Gd(4)-O(3) | 2.359(4) |
| Gd(2)-O(23) | 2.377(5) | Gd(4)-O(15) | 2.361(5) |
| Gd(2)-O(2) | 2.389(4) | Gd(4)-O(1) | 2.374(4) |
| Gd(2)-O(20) | 2.390(5) | Gd(4)-O(10) | 2.380(5) |
| Gd(2)-O(1) | 2.426(4) | Gd(4)-O(27) | 2.404(5) |
| Gd(2)-O(26) | 2.467(6) | Gd(4)-O(11) | 2.482(5) |
| Gd(2)-O(4) | 2.460(4) | Gd(4)-O(13) | 2.511(5) |
|  |  |  |  |
| O(25)-Gd(1)-O(2) | 145.33(18) | C(40)-C(45)-C(32) | 119.0(12) |
| O(25)-Gd(1)-O(4) | 83.92(18) | O(16)-C(46)-O(17) | 122.6(7) |
| O(2)-Gd(1)-O(4) | 72.88(15) | O(16)-C(46)-C(47) | 118.7(7) |
| O(25)-Gd(1)-O(9) | 92.9(2) | O(17)-C(46)-C(47) | 118.8(7) |
| O(2)-Gd(1)-O(9) | 89.49(18) | C(60)-C(47)-C(48) | 120.3(8) |
| O(4)-Gd(1)-O(9) | 140.91(17) | C(60)-C(47)-C(46) | 121.1(8) |
| O(25)-Gd(1)-O(3) | 77.27(19) | C(48)-C(47)-C(46) | 118.6(8) |
| O(2)-Gd(1)-O(3) | 70.48(16) | C(53)-C(48)-C(49) | 117.7(8) |
| O(4)-Gd(1)-O(3) | 68.55(15) | C(53)-C(48)-C(47) | 119.4(8) |
| O(9)-Gd(1)-O(3) | 72.75(17) | C(49)-C(48)-C(47) | 122.9(8) |
| O(25)-Gd(1)-O(5) | 101.4(2) | C(50)-C(49)-C(48) | 121.8(9) |
| O(2)-Gd(1)-O(5) | 95.00(17) | C(49)-C(50)-C(51) | 119.2(10) |
| O(4)-Gd(1)-O(5) | 70.75(16) | C(52)-C(51)-C(50) | 121.5(10) |
| O(9)-Gd(1)-O(5) | 147.08(18) | C(51)-C(52)-C(53) | 120.9(9) |
| O(3)-Gd(1)-O(5) | 139.19(16) | C(54)-C(53)-C(48) | 118.4(9) |
| O(25)-Gd(1)-O(8) | 133.74(19) | C(54)-C(53)-C(52) | 122.7(9) |
| O(2)-Gd(1)-O(8) | 80.02(16) | C(48)-C(53)-C(52) | 118.8(9) |
| O(4)-Gd(1)-O(8) | 133.22(16) | C(55)-C(54)-C(53) | 123.7(9) |
| O(9)-Gd(1)-O(8) | 74.16(19) | C(54)-C(55)-C(60) | 118.7(9) |
| O(3)-Gd(1)-O(8) | 135.25(16) | C(54)-C(55)-C(56) | 124.1(9) |
| O(5)-Gd(1)-O(8) | 74.56(18) | C(60)-C(55)-C(56) | 117.1(9) |
| O(25)-Gd(1)-O(7) | 81.04(19) | C(57)-C(56)-C(55) | 121.5(9) |
| O(2)-Gd(1)-O(7) | 132.81(16) | C(56)-C(57)-C(58) | 121.4(10) |
| O(4)-Gd(1)-O(7) | 140.61(16) | C(59)-C(58)-C(57) | 119.5(10) |
| O(9)-Gd(1)-O(7) | 76.28(19) | C(58)-C(59)-C(60) | 122.4(9) |
| O(3)-Gd(1)-O(7) | 140.85(17) | C(47)-C(60)-C(59) | 122.7(8) |
| O(5)-Gd(1)-O(7) | 76.86(18) | C(47)-C(60)-C(55) | 119.4(8) |
| O(8)-Gd(1)-O(7) | 52.88(17) | C(59)-C(60)-C(55) | 117.9(8) |
| O(6)-Gd(2)-O(19) | 96.9(2) | O(18)-C(61)-O(19) | 125.7(8) |
| O(6)-Gd(2)-O(23) | 149.8(2) | O(18)-C(61)-C(62) | 117.7(8) |
| O(19)-Gd(2)-O(23) | 94.4(2) | O(19)-C(61)-C(62) | 116.7(8) |
| O(6)-Gd(2)-O(2) | 99.68(17) | C(63)-C(62)-C(75) | 121.9(9) |
| O(19)-Gd(2)-O(2) | 142.61(18) | C(63)-C(62)-C(61) | 119.5(10) |
| O(23)-Gd(2)-O(2) | 87.78(19) | C(75)-C(62)-C(61) | 118.6(10) |
| O(6)-Gd(2)-O(20) | 76.54(19) | C(64)-C(63)-C(62) | 123.6(9) |
| O(19)-Gd(2)-O(20) | 139.8(2) | C(64)-C(63)-C(68) | 119.9(10) |
| O(23)-Gd(2)-O(20) | 76.8(2) | C(62)-C(63)-C(68) | 116.5(11) |
| O(2)-Gd(2)-O(20) | 76.93(17) | C(65)-C(64)-C(63) | 120.9(11) |
| O(6)-Gd(2)-O(1) | 136.19(17) | C(64)-C(65)-C(66) | 118.7(13) |
| O(19)-Gd(2)-O(1) | 74.33(18) | C(67)-C(66)-C(65) | 122.5(13) |
| O(23)-Gd(2)-O(1) | 73.88(17) | C(66)-C(67)-C(68) | 122.9(12) |
| O(2)-Gd(2)-O(1) | 70.47(15) | C(69)-C(68)-C(67) | 125.0(11) |
| O(20)-Gd(2)-O(1) | 136.42(16) | C(69)-C(68)-C(63) | 119.9(11) |
| O(6)-Gd(2)-O(26) | 79.43(19) | C(67)-C(68)-C(63) | 115.1(11) |
| O(19)-Gd(2)-O(26) | 70.0(2) | C(70)-C(69)-C(68) | 124.2(11) |
| O(23)-Gd(2)-O(26) | 78.3(2) | C(69)-C(70)-C(75) | 118.6(11) |
| O(2)-Gd(2)-O(26) | 146.1(2) | C(69)-C(70)-C(71) | 124.7(11) |
| O(20)-Gd(2)-O(26) | 69.9(2) | C(75)-C(70)-C(71) | 116.7(11) |
| O(1)-Gd(2)-O(26) | 132.32(19) | C(72)-C(71)-C(70) | 123.4(11) |
| O(6)-Gd(2)-O(4) | 69.20(16) | C(71)-C(72)-C(73) | 118.4(12) |
| O(19)-Gd(2)-O(4) | 84.01(18) | C(72)-C(73)-C(74) | 122.1(13) |
| O(23)-Gd(2)-O(4) | 140.04(17) | C(73)-C(74)-C(75) | 121.8(11) |
| O(2)-Gd(2)-O(4) | 71.20(15) | C(74)-C(75)-C(62) | 123.5(9) |
| O(20)-Gd(2)-O(4) | 127.46(17) | C(74)-C(75)-C(70) | 117.6(11) |
| O(1)-Gd(2)-O(4) | 67.23(15) | C(62)-C(75)-C(70) | 118.9(11) |
| O(26)-Gd(2)-O(4) | 136.23(18) | O(27)-C(76)-O(23) | 125.6(7) |
| O(4)-Gd(3)-O(14) | 145.02(18) | O(27)-C(76)-C(77) | 117.2(7) |
| O(4)-Gd(3)-O(18) | 90.76(19) | O(23)-C(76)-C(77) | 117.1(7) |
| O(14)-Gd(3)-O(18) | 94.1(2) | C(90)-C(77)-C(78) | 119.3(7) |
| O(4)-Gd(3)-O(3) | 71.07(15) | C(90)-C(77)-C(76) | 119.7(7) |
| O(14)-Gd(3)-O(3) | 84.42(18) | C(78)-C(77)-C(76) | 121.0(7) |
| O(18)-Gd(3)-O(3) | 142.25(18) | C(77)-C(78)-C(83) | 120.0(8) |
| O(4)-Gd(3)-O(1) | 70.05(16) | C(77)-C(78)-C(79) | 122.2(7) |
| O(14)-Gd(3)-O(1) | 78.01(18) | C(83)-C(78)-C(79) | 117.8(8) |
| O(18)-Gd(3)-O(1) | 74.30(18) | C(80)-C(79)-C(78) | 121.2(8) |
| O(3)-Gd(3)-O(1) | 68.49(15) | C(79)-C(80)-C(81) | 120.0(9) |
| O(4)-Gd(3)-O(24) | 94.80(19) | C(82)-C(81)-C(80) | 120.5(9) |
| O(14)-Gd(3)-O(24) | 100.9(2) | C(81)-C(82)-C(83) | 121.9(9) |
| O(18)-Gd(3)-O(24) | 144.5(2) | C(84)-C(83)-C(78) | 119.2(8) |
| O(3)-Gd(3)-O(24) | 71.80(17) | C(84)-C(83)-C(82) | 122.1(8) |
| O(1)-Gd(3)-O(24) | 140.19(17) | C(78)-C(83)-C(82) | 118.6(8) |
| O(4)-Gd(3)-O(17) | 128.15(16) | C(83)-C(84)-C(85) | 122.4(8) |
| Symmetry codes: ##1 -x+1,-y+1,-z+2 | | | |

**Table S14**. Selected bond lengths (Å) and angles (^o^) for compound **3** at 293(2) K.

| Compound **3** | | | |
| --- | --- | --- | --- |
| O(1)-Y(1) | 2.419(3) | O(19)-Y(3) | 2.439(3) |
| O(2)-Y(1) | 2.423(3) | O(20)-Y(3) | 2.340(3) |
| O(3)-Y(1) | 2.337(3) | O(21)-Y(1) | 2.359(3) |
| O(4)-Y(4) | 2.335(3) | O(22)-Y(4) | 2.298(2) |
| O(5)-Y(2) | 2.359(3) | O(22)-Y(1) | 2.305(2) |
| O(7)-Y(2) | 2.295(3) | O(22)-Y(2) | 2.351(2) |
| O(8)-Y(1) | 2.359(3) | O(23)-Y(3) | 2.267(2) |
| O(9)-Y(4) | 2.447(3) | O(23)-Y(1) | 2.369(2) |
| O(10)-Y(4) | 2.465(3) | O(23)-Y(2) | 2.412(2) |
| O(12)-Y(4) | 2.356(3) | O(24)-Y(4) | 2.340(2) |
| O(13)-Y(2) | 2.321(2) | O(24)-Y(3) | 2.351(2) |
| O(14)-Y(4) | 2.302(3) | O(24)-Y(2) | 2.374(2) |
| O(15)-Y(3) | 2.313(3) | O(25)-Y(4) | 2.315(2) |
| O(16)-Y(2) | 2.344(3) | O(25)-Y(3) | 2.336(2) |
| O(17)-Y(3) | 2.325(3) | O(25)-Y(1) | 2.371(3) |
| O(18)-Y(3) | 2.378(2) | O(26)-Y(2) | 2.452(3) |
|  |  |  |  |
| O(2)-C(16)-O(1) | 120.4(4) | C(109)-C(108)-C(107) | 123.2(4) |
| O(2)-C(16)-C(17) | 120.7(4) | C(109)-C(108)-C(113) | 118.3(5) |
| O(1)-C(16)-C(17) | 118.9(4) | C(107)-C(108)-C(113) | 118.5(5) |
| C(30)-C(17)-C(18) | 122.0(4) | C(110)-C(109)-C(108) | 120.7(5) |
| C(30)-C(17)-C(16) | 119.0(5) | C(109)-C(110)-C(111) | 119.6(7) |
| C(18)-C(17)-C(16) | 118.9(4) | C(112)-C(111)-C(110) | 121.6(6) |
| C(17)-C(30)-C(29) | 122.4(4) | C(111)-C(112)-C(113) | 121.1(5) |
| C(17)-C(30)-C(25) | 118.4(5) | C(114)-C(113)-C(108) | 119.2(5) |
| C(29)-C(30)-C(25) | 119.2(5) | C(114)-C(113)-C(112) | 122.0(5) |
| C(28)-C(29)-C(30) | 121.0(5) | C(108)-C(113)-C(112) | 118.8(6) |
| C(29)-C(28)-C(27) | 119.4(6) | C(113)-C(114)-C(115) | 123.0(5) |
| C(26)-C(27)-C(28) | 121.3(6) | C(114)-C(115)-C(120) | 118.9(6) |
| C(27)-C(26)-C(25) | 121.9(5) | C(114)-C(115)-C(116) | 123.8(5) |
| C(24)-C(25)-C(30) | 119.9(5) | C(120)-C(115)-C(116) | 117.2(6) |
| C(24)-C(25)-C(26) | 122.7(5) | C(117)-C(116)-C(115) | 123.2(5) |
| C(30)-C(25)-C(26) | 117.3(6) | C(116)-C(117)-C(118) | 118.2(6) |
| C(23)-C(24)-C(25) | 122.1(4) | C(119)-C(118)-C(117) | 122.4(7) |
| C(24)-C(23)-C(22) | 123.7(5) | C(118)-C(119)-C(120) | 121.6(5) |
| C(24)-C(23)-C(18) | 118.6(5) | C(107)-C(120)-C(119) | 123.4(4) |
| C(22)-C(23)-C(18) | 117.7(5) | C(107)-C(120)-C(115) | 119.2(5) |
| C(21)-C(22)-C(23) | 121.6(5) | C(119)-C(120)-C(115) | 117.4(5) |
| C(22)-C(21)-C(20) | 120.7(6) | O(19)-C(121)-O(18) | 121.2(3) |
| C(19)-C(20)-C(21) | 120.0(6) | O(19)-C(121)-C(122) | 120.2(3) |
| C(20)-C(19)-C(18) | 121.6(5) | O(18)-C(121)-C(122) | 118.5(3) |
| C(17)-C(18)-C(19) | 122.8(4) | C(135)-C(122)-C(123) | 121.6(4) |
| C(17)-C(18)-C(23) | 118.8(5) | C(135)-C(122)-C(121) | 119.7(3) |
| C(19)-C(18)-C(23) | 118.3(5) | C(123)-C(122)-C(121) | 118.7(3) |
| O(8)-C(15)-O(7) | 124.4(4) | C(122)-C(123)-C(124) | 122.5(3) |
| O(8)-C(15)-C(1) | 119.2(4) | C(122)-C(123)-C(128) | 119.4(4) |
| O(7)-C(15)-C(1) | 116.4(4) | C(124)-C(123)-C(128) | 118.1(4) |
| C(14)-C(1)-C(2) | 122.1(5) | C(125)-C(124)-C(123) | 120.6(4) |
| C(14)-C(1)-C(15) | 119.3(5) | C(124)-C(125)-C(126) | 121.1(5) |
| C(2)-C(1)-C(15) | 118.4(5) | C(127)-C(126)-C(125) | 119.6(4) |
| C(3)-C(2)-C(1) | 125.2(5) | C(126)-C(127)-C(128) | 121.5(4) |
| C(3)-C(2)-C(7) | 117.6(6) | C(127)-C(128)-C(129) | 122.5(4) |
| C(1)-C(2)-C(7) | 117.2(6) | C(127)-C(128)-C(123) | 119.0(4) |
| C(4)-C(3)-C(2) | 121.1(6) | C(129)-C(128)-C(123) | 118.5(4) |
| C(3)-C(4)-C(5) | 121.0(8) | C(130)-C(129)-C(128) | 122.1(4) |
| C(6)-C(5)-C(4) | 120.5(7) | C(129)-C(130)-C(135) | 119.8(4) |
| C(5)-C(6)-C(7) | 121.6(6) | C(129)-C(130)-C(131) | 121.8(4) |
| C(8)-C(7)-C(6) | 122.7(6) | C(135)-C(130)-C(131) | 118.4(4) |
| C(8)-C(7)-C(2) | 119.2(6) | C(132)-C(131)-C(130) | 121.4(4) |
| C(6)-C(7)-C(2) | 118.0(7) | C(131)-C(132)-C(133) | 119.8(4) |
| C(7)-C(8)-C(9) | 123.9(5) | C(134)-C(133)-C(132) | 121.0(5) |
| C(8)-C(9)-C(10) | 122.4(6) | C(133)-C(134)-C(135) | 121.2(4) |
| C(8)-C(9)-C(14) | 119.4(6) | C(122)-C(135)-C(134) | 123.4(4) |
| C(10)-C(9)-C(14) | 118.2(6) | C(122)-C(135)-C(130) | 118.5(4) |
| C(11)-C(10)-C(9) | 122.7(6) | C(134)-C(135)-C(130) | 118.1(4) |
| C(10)-C(11)-C(12) | 118.8(7) | O(11)-N(1)-O(10) | 122.2(3) |
| C(13)-C(12)-C(11) | 120.4(7) | O(11)-N(1)-O(9) | 121.7(3) |
| C(12)-C(13)-C(14) | 121.7(5) | O(10)-N(1)-O(9) | 116.1(3) |
| C(13)-C(14)-C(1) | 123.7(5) | C(16)-O(1)-Y(1) | 93.1(2) |
| C(13)-C(14)-C(9) | 118.3(6) | C(16)-O(2)-Y(1) | 92.6(2) |
| C(1)-C(14)-C(9) | 118.0(6) | C(46)-O(3)-Y(1) | 136.9(3) |
| O(20)-C(31)-O(21) | 124.0(4) | C(46)-O(4)-Y(4) | 134.3(3) |
| O(20)-C(31)-C(32) | 119.3(4) | C(61)-O(5)-Y(2) | 138.4(3) |
| O(21)-C(31)-C(32) | 116.6(5) | C(15)-O(7)-Y(2) | 134.6(3) |
| C(45)-C(32)-C(33) | 117.2(6) | C(15)-O(8)-Y(1) | 137.2(3) |
| C(45)-C(32)-C(31) | 122.7(7) | N(1)-O(9)-Y(4) | 96.4(2) |
| C(33)-C(32)-C(31) | 120.0(6) | N(1)-O(10)-Y(4) | 95.6(2) |
| C(34)-C(33)-C(32) | 125.0(6) | C(76)-O(12)-Y(4) | 132.3(2) |
| C(34)-C(33)-C(38) | 112.3(7) | C(76)-O(13)-Y(2) | 138.2(2) |
| C(32)-C(33)-C(38) | 122.7(7) | C(91)-O(14)-Y(4) | 135.1(2) |
| C(33)-C(34)-C(35) | 121.9(7) | C(91)-O(15)-Y(3) | 137.2(2) |
| C(34)-C(35)-C(36) | 120.1(10) | C(106)-O(16)-Y(2) | 138.1(2) |
| C(37)-C(36)-C(35) | 120.2(9) | C(106)-O(17)-Y(3) | 133.6(2) |
| C(38)-C(37)-C(36) | 117.3(7) | C(121)-O(18)-Y(3) | 93.3(2) |
| C(37)-C(38)-C(39) | 117.7(8) | C(121)-O(19)-Y(3) | 90.9(2) |
| C(37)-C(38)-C(33) | 128.2(9) | C(31)-O(20)-Y(3) | 133.5(3) |
| C(39)-C(38)-C(33) | 113.9(9) | C(31)-O(21)-Y(1) | 135.2(3) |
| C(38)-C(39)-C(40) | 127.8(8) | Y(4)-O(22)-Y(1) | 106.24(10) |
| C(41)-C(40)-C(39) | 127.3(7) | Y(4)-O(22)-Y(2) | 106.02(9) |

**Table S15**. Selected bond lengths (Å) and angles (^o^) for compound **3m** at 293(2) K.

| Compound **3m** | | | |
| --- | --- | --- | --- |
| Y(1)-O(19) | 2.306(4) | Y(3)-O(19) | 2.307(4) |
| Y(1)-O(14) | 2.303(4) | Y(3)-O(7) | 2.323(5) |
| Y(1)-O(20) | 2.313(4) | Y(3)-O(22) | 2.362(4) |
| Y(1)-O(6) | 2.333(5) | Y(3)-O(5) | 2.356(5) |
| Y(1)-O(21) | 2.332(4) | Y(3)-O(20) | 2.397(4) |
| Y(1)-O(16) | 2.360(4) | Y(3)-O(2) | 2.420(5) |
| Y(1)-O(23) | 2.432(5) | Y(3)-O(3) | 2.403(4) |
| Y(1)-O(24) | 2.463(5) | Y(3)-O(4) | 2.435(5) |
| Y(2)-O(1) | 2.299(5) | Y(4)-O(22) | 2.268(4) |
| Y(2)-O(15) | 2.319(4) | Y(4)-O(13) | 2.310(5) |
| Y(2)-O(12) | 2.331(5) | Y(4)-O(20) | 2.335(4) |
| Y(2)-O(19) | 2.350(4) | Y(4)-O(11) | 2.346(5) |
| Y(2)-O(17) | 2.360(5) | Y(4)-O(8) | 2.355(5) |
| Y(2)-O(21) | 2.384(4) | Y(4)-O(21) | 2.360(4) |
| Y(2)-O(22) | 2.417(4) | Y(4)-O(10) | 2.393(4) |
| Y(2)-O(26) | 2.449(5) | Y(4)-O(9) | 2.429(5) |
|  |  |  |  |
| O(19)-Y(1)-O(14) | 148.18(16) | O(5)-C(46)-C(47) | 118.2(7) |
| O(19)-Y(1)-O(20) | 72.37(14) | C(48)-C(47)-C(60) | 122.2(7) |
| O(14)-Y(1)-O(20) | 83.35(15) | C(48)-C(47)-C(46) | 118.8(7) |
| O(19)-Y(1)-O(6) | 94.52(17) | C(60)-C(47)-C(46) | 119.0(7) |
| O(14)-Y(1)-O(6) | 97.76(18) | C(47)-C(48)-C(49) | 128.1(8) |
| O(20)-Y(1)-O(6) | 73.01(16) | C(47)-C(48)-C(53) | 117.0(9) |
| O(19)-Y(1)-O(21) | 72.50(14) | C(49)-C(48)-C(53) | 112.9(9) |
| O(14)-Y(1)-O(21) | 79.91(15) | C(50)-C(49)-C(48) | 126.9(11) |
| O(20)-Y(1)-O(21) | 69.18(14) | C(49)-C(50)-C(51) | 117.2(12) |
| O(6)-Y(1)-O(21) | 142.15(16) | C(52)-C(51)-C(50) | 121.1(13) |
| O(19)-Y(1)-O(16) | 98.60(16) | C(51)-C(52)-C(53) | 119.6(14) |
| O(14)-Y(1)-O(16) | 87.92(17) | C(54)-C(53)-C(52) | 119.5(13) |
| O(20)-Y(1)-O(16) | 142.04(15) | C(54)-C(53)-C(48) | 118.7(9) |
| O(6)-Y(1)-O(16) | 144.92(16) | C(52)-C(53)-C(48) | 121.7(13) |
| O(21)-Y(1)-O(16) | 72.93(14) | C(53)-C(54)-C(55) | 122.9(11) |
| O(19)-Y(1)-O(23) | 79.13(15) | C(54)-C(55)-C(60) | 122.7(11) |
| O(14)-Y(1)-O(23) | 132.38(16) | C(54)-C(55)-C(56) | 121.5(11) |
| O(20)-Y(1)-O(23) | 135.28(16) | C(60)-C(55)-C(56) | 115.5(11) |
| O(6)-Y(1)-O(23) | 75.75(17) | C(57)-C(56)-C(55) | 122.4(11) |
| O(21)-Y(1)-O(23) | 132.78(15) | C(56)-C(57)-C(58) | 118.1(12) |
| O(16)-Y(1)-O(23) | 75.02(17) | C(59)-C(58)-C(57) | 121.9(12) |
| O(19)-Y(1)-O(24) | 131.73(16) | C(58)-C(59)-C(60) | 123.3(9) |
| O(14)-Y(1)-O(24) | 79.98(17) | C(59)-C(60)-C(55) | 118.6(9) |
| O(20)-Y(1)-O(24) | 140.70(16) | C(59)-C(60)-C(47) | 125.2(7) |
| O(6)-Y(1)-O(24) | 74.33(17) | C(55)-C(60)-C(47) | 116.1(9) |
| O(21)-Y(1)-O(24) | 140.45(16) | O(16)-C(61)-O(15) | 126.3(6) |
| O(16)-Y(1)-O(24) | 72.64(16) | O(16)-C(61)-C(62) | 117.8(6) |
| O(23)-Y(1)-O(24) | 52.62(16) | O(15)-C(61)-C(62) | 115.9(6) |
| O(1)-Y(2)-O(15) | 147.46(19) | C(63)-C(62)-C(75) | 122.2(6) |
| O(1)-Y(2)-O(12) | 97.10(18) | C(63)-C(62)-C(61) | 118.3(6) |
| O(15)-Y(2)-O(12) | 92.35(19) | C(75)-C(62)-C(61) | 119.5(6) |
| O(1)-Y(2)-O(19) | 100.68(15) | C(68)-C(63)-C(64) | 118.7(6) |
| O(15)-Y(2)-O(19) | 89.38(16) | C(68)-C(63)-C(62) | 118.1(6) |
| O(12)-Y(2)-O(19) | 143.74(16) | C(64)-C(63)-C(62) | 123.2(6) |
| O(1)-Y(2)-O(17) | 76.31(18) | C(65)-C(64)-C(63) | 121.1(8) |
| O(15)-Y(2)-O(17) | 75.88(18) | C(64)-C(65)-C(66) | 119.6(8) |
| O(12)-Y(2)-O(17) | 137.95(17) | C(67)-C(66)-C(65) | 121.0(8) |
| O(19)-Y(2)-O(17) | 77.35(15) | C(66)-C(67)-C(68) | 120.7(8) |
| O(1)-Y(2)-O(21) | 137.80(16) | C(69)-C(68)-C(63) | 118.8(7) |
| O(15)-Y(2)-O(21) | 74.74(16) | C(69)-C(68)-C(67) | 122.3(7) |
| O(12)-Y(2)-O(21) | 74.78(15) | C(63)-C(68)-C(67) | 118.9(7) |
| O(19)-Y(2)-O(21) | 70.79(13) | C(68)-C(69)-C(70) | 123.6(7) |
| O(17)-Y(2)-O(21) | 136.35(15) | C(71)-C(70)-C(69) | 121.8(7) |
| O(1)-Y(2)-O(22) | 70.38(16) | C(71)-C(70)-C(75) | 119.6(8) |
| O(15)-Y(2)-O(22) | 141.70(16) | C(69)-C(70)-C(75) | 118.5(7) |
| O(12)-Y(2)-O(22) | 85.15(16) | C(72)-C(71)-C(70) | 121.0(9) |
| O(19)-Y(2)-O(22) | 71.69(14) | C(71)-C(72)-C(73) | 119.4(9) |
| O(17)-Y(2)-O(22) | 128.45(16) | C(74)-C(73)-C(72) | 121.4(9) |
| O(21)-Y(2)-O(22) | 67.73(14) | C(73)-C(74)-C(75) | 121.7(8) |
| O(1)-Y(2)-O(26) | 77.08(18) | C(74)-C(75)-C(62) | 124.4(6) |
| O(15)-Y(2)-O(26) | 77.44(18) | C(74)-C(75)-C(70) | 116.8(7) |
| O(12)-Y(2)-O(26) | 69.0(2) | C(62)-C(75)-C(70) | 118.7(7) |
| O(19)-Y(2)-O(26) | 145.93(18) | O(14)-C(76)-O(13) | 124.8(6) |
| O(17)-Y(2)-O(26) | 69.04(19) | O(14)-C(76)-C(77) | 118.3(6) |
| O(21)-Y(2)-O(26) | 132.76(18) | O(13)-C(76)-C(77) | 116.9(6) |
| O(22)-Y(2)-O(26) | 135.22(16) | C(78)-C(77)-C(90) | 120.1(7) |
| O(19)-Y(3)-O(7) | 146.75(17) | C(78)-C(77)-C(76) | 120.1(6) |
| O(19)-Y(3)-O(22) | 73.43(14) | C(90)-C(77)-C(76) | 119.8(7) |
| O(7)-Y(3)-O(22) | 83.79(16) | C(77)-C(78)-C(83) | 120.6(7) |
| O(19)-Y(3)-O(5) | 91.74(17) | C(77)-C(78)-C(79) | 122.8(7) |
| O(7)-Y(3)-O(5) | 91.30(19) | C(83)-C(78)-C(79) | 116.5(8) |
| O(22)-Y(3)-O(5) | 141.38(16) | C(80)-C(79)-C(78) | 122.6(11) |
| O(19)-Y(3)-O(20) | 70.82(14) | C(79)-C(80)-C(81) | 120.2(12) |
| O(7)-Y(3)-O(20) | 78.58(17) | C(82)-C(81)-C(80) | 121.9(12) |
| O(22)-Y(3)-O(20) | 68.87(14) | C(81)-C(82)-C(83) | 120.2(11) |
| O(5)-Y(3)-O(20) | 72.60(16) | C(84)-C(83)-C(78) | 118.4(8) |
| O(19)-Y(3)-O(2) | 93.84(17) | C(84)-C(83)-C(82) | 123.1(9) |
| O(7)-Y(3)-O(2) | 101.6(2) | C(78)-C(83)-C(82) | 118.5(9) |
| O(22)-Y(3)-O(2) | 71.40(16) | C(85)-C(84)-C(83) | 124.6(8) |
| O(5)-Y(3)-O(2) | 146.49(18) | C(84)-C(85)-C(86) | 124.4(10) |
| O(20)-Y(3)-O(2) | 140.01(16) | C(84)-C(85)-C(90) | 118.1(8) |
| O(19)-Y(3)-O(3) | 79.85(16) | C(86)-C(85)-C(90) | 117.5(10) |

**Table S16**. Selected bond lengths (Å) and angles (^o^) for compound **3a** at 293(2) K.

| Compound **3a** | | | |
| --- | --- | --- | --- |
| O(1)-Y(4) | 2.3092(19) | O(9)-Y(3) | 2.433(2) |
| O(1)-Y(3) | 2.331(2) | O(10)-Y(3) | 2.380(2) |
| O(1)-Y(1) | 2.389(2) | O(11)-Y(3) | 2.337(2) |
| O(2)-Y(4) | 2.2970(19) | O(12)-Y(2) | 2.322(2) |
| O(2)-Y(1) | 2.3006(19) | O(13)-Y(3) | 2.306(2) |
| O(2)-Y(2) | 2.342(2) | O(14)-Y(4) | 2.316(2) |
| O(3)-Y(3) | 2.2618(19) | O(15)-Y(4) | 2.368(2) |
| O(3)-Y(1) | 2.3561(19) | O(16)-Y(2) | 2.323(2) |
| O(3)-Y(2) | 2.4192(19) | O(18)-Y(2) | 2.372(2) |
| O(4)-Y(4) | 2.3289(19) | O(19)-Y(4) | 2.469(2) |
| O(4)-Y(3) | 2.3638(19) | O(20)-Y(4) | 2.451(2) |
| O(4)-Y(2) | 2.3882(19) | O(22)-Y(2) | 2.432(2) |
| O(5)-Y(1) | 2.411(2) | O(23)-Y(4) | 2.327(2) |
| O(6)-Y(2) | 2.294(2) | O(24)-Y(1) | 2.352(2) |
| O(7)-Y(1) | 2.332(2) | O(25)-Y(1) | 2.414(2) |
| O(8)-Y(3) | 2.362(2) | O(26)-Y(1) | 2.433(2) |
|  |  |  |  |
| O(5)-C(15)-O(6) | 124.3(3) | C(120)-C(107)-C(108) | 121.9(3) |
| O(5)-C(15)-C(1) | 121.2(3) | C(120)-C(107)-C(106) | 119.1(3) |
| O(6)-C(15)-C(1) | 114.5(3) | C(108)-C(107)-C(106) | 119.0(3) |
| C(14)-C(1)-C(2) | 106.9(2) | C(109)-C(108)-C(107) | 122.7(3) |
| C(14)-C(1)-C(15) | 107.9(2) | C(109)-C(108)-C(113) | 119.0(3) |
| C(2)-C(1)-C(15) | 117.3(2) | C(107)-C(108)-C(113) | 118.3(4) |
| C(14)-C(1)-C(8)#1 | 110.3(2) | C(110)-C(109)-C(108) | 121.0(4) |
| C(2)-C(1)-C(8)#1 | 108.4(2) | C(109)-C(110)-C(111) | 120.0(5) |
| C(15)-C(1)-C(8)#1 | 106.1(2) | C(112)-C(111)-C(110) | 120.9(4) |
| C(9)-C(14)-C(13) | 119.2(3) | C(111)-C(112)-C(113) | 121.3(4) |
| C(9)-C(14)-C(1) | 116.0(3) | C(114)-C(113)-C(112) | 122.9(4) |
| C(13)-C(14)-C(1) | 124.8(3) | C(114)-C(113)-C(108) | 119.2(4) |
| C(14)-C(13)-C(12) | 120.2(3) | C(112)-C(113)-C(108) | 117.9(4) |
| C(11)-C(12)-C(13) | 120.3(3) | C(115)-C(114)-C(113) | 122.6(3) |
| C(12)-C(11)-C(10) | 119.3(3) | C(114)-C(115)-C(120) | 118.5(4) |
| C(9)-C(10)-C(11) | 120.9(3) | C(114)-C(115)-C(116) | 122.9(4) |
| C(10)-C(9)-C(14) | 120.0(3) | C(120)-C(115)-C(116) | 118.6(4) |
| C(10)-C(9)-C(8) | 121.3(3) | C(117)-C(116)-C(115) | 121.2(4) |
| C(14)-C(9)-C(8) | 118.5(3) | C(116)-C(117)-C(118) | 120.3(4) |
| C(7)-C(8)-C(9) | 108.7(3) | C(119)-C(118)-C(117) | 120.7(5) |
| C(7)-C(8)-C(1)#1 | 113.1(2) | C(118)-C(119)-C(120) | 120.9(4) |
| C(9)-C(8)-C(1)#1 | 114.7(2) | C(107)-C(120)-C(119) | 122.3(3) |
| C(6)-C(7)-C(2) | 120.1(3) | C(107)-C(120)-C(115) | 119.3(3) |
| C(6)-C(7)-C(8) | 120.7(3) | C(119)-C(120)-C(115) | 118.3(4) |
| C(2)-C(7)-C(8) | 119.3(3) | O(24)-C(121)-O(23) | 124.8(3) |
| C(5)-C(6)-C(7) | 120.2(4) | O(24)-C(121)-C(122) | 118.1(3) |
| C(6)-C(5)-C(4) | 120.3(4) | O(23)-C(121)-C(122) | 117.1(3) |
| C(5)-C(4)-C(3) | 120.6(4) | C(135)-C(122)-C(123) | 120.7(3) |
| C(2)-C(3)-C(4) | 120.0(4) | C(135)-C(122)-C(121) | 119.8(3) |
| C(3)-C(2)-C(7) | 118.9(3) | C(123)-C(122)-C(121) | 119.5(3) |
| C(3)-C(2)-C(1) | 125.9(3) | C(122)-C(123)-C(124) | 123.8(3) |
| C(7)-C(2)-C(1) | 115.1(3) | C(122)-C(123)-C(128) | 119.3(3) |
| O(17)-C(16)-O(18) | 125.5(3) | C(124)-C(123)-C(128) | 116.9(3) |
| O(17)-C(16)-C(17) | 117.4(3) | C(125)-C(124)-C(123) | 121.6(4) |
| O(18)-C(16)-C(17) | 117.0(3) | C(124)-C(125)-C(126) | 120.5(4) |
| C(30)-C(17)-C(18) | 121.8(3) | C(127)-C(126)-C(125) | 120.4(4) |
| C(30)-C(17)-C(16) | 119.0(3) | C(126)-C(127)-C(128) | 120.9(4) |
| C(18)-C(17)-C(16) | 119.1(3) | C(129)-C(128)-C(127) | 121.1(4) |
| C(17)-C(18)-C(19) | 122.9(3) | C(129)-C(128)-C(123) | 119.2(4) |
| C(17)-C(18)-C(23) | 118.7(3) | C(127)-C(128)-C(123) | 119.7(4) |
| C(19)-C(18)-C(23) | 118.3(3) | C(130)-C(129)-C(128) | 122.5(4) |
| C(20)-C(19)-C(18) | 120.9(4) | C(129)-C(130)-C(131) | 120.8(4) |
| C(19)-C(20)-C(21) | 120.6(4) | C(129)-C(130)-C(135) | 119.4(4) |
| C(22)-C(21)-C(20) | 120.8(4) | C(131)-C(130)-C(135) | 119.8(4) |
| C(21)-C(22)-C(23) | 121.2(4) | C(132)-C(131)-C(130) | 121.6(5) |
| C(24)-C(23)-C(22) | 122.5(3) | C(131)-C(132)-C(133) | 119.4(5) |
| C(24)-C(23)-C(18) | 119.3(3) | C(134)-C(133)-C(132) | 121.5(5) |
| C(22)-C(23)-C(18) | 118.1(4) | C(133)-C(134)-C(135) | 121.4(4) |
| C(25)-C(24)-C(23) | 122.3(3) | C(122)-C(135)-C(134) | 124.8(3) |
| C(24)-C(25)-C(26) | 122.2(4) | C(122)-C(135)-C(130) | 118.8(3) |
| C(24)-C(25)-C(30) | 118.9(4) | C(134)-C(135)-C(130) | 116.2(4) |
| C(26)-C(25)-C(30) | 118.9(4) | O(21)-N(1)-O(19) | 122.7(3) |
| C(27)-C(26)-C(25) | 120.8(4) | O(21)-N(1)-O(20) | 121.3(3) |
| C(26)-C(27)-C(28) | 121.1(4) | O(19)-N(1)-O(20) | 116.0(2) |
| C(29)-C(28)-C(27) | 120.0(5) | Y(4)-O(1)-Y(3) | 109.22(8) |
| C(28)-C(29)-C(30) | 121.0(4) | Y(4)-O(1)-Y(1) | 103.84(8) |
| C(17)-C(30)-C(25) | 118.8(3) | Y(3)-O(1)-Y(1) | 105.00(8) |
| C(17)-C(30)-C(29) | 122.9(3) | Y(4)-O(2)-Y(1) | 107.13(8) |
| C(25)-C(30)-C(29) | 118.2(4) | Y(4)-O(2)-Y(2) | 106.28(8) |
| O(15)-C(31)-O(16) | 125.9(3) | Y(1)-O(2)-Y(2) | 105.68(7) |
| O(15)-C(31)-C(32) | 117.8(3) | Y(3)-O(3)-Y(1) | 108.36(8) |
| O(16)-C(31)-C(32) | 116.3(3) | Y(3)-O(3)-Y(2) | 109.39(8) |
| C(33)-C(32)-C(45) | 120.4(3) | Y(1)-O(3)-Y(2) | 101.56(7) |
| C(33)-C(32)-C(31) | 119.7(3) | Y(4)-O(4)-Y(3) | 107.43(7) |
| C(45)-C(32)-C(31) | 119.9(3) | Y(4)-O(4)-Y(2) | 103.77(7) |
| C(32)-C(33)-C(38) | 120.0(3) | Y(3)-O(4)-Y(2) | 107.05(8) |
| C(32)-C(33)-C(34) | 122.6(3) | C(15)-O(5)-Y(1) | 133.46(19) |
| C(38)-C(33)-C(34) | 117.4(3) | C(15)-O(6)-Y(2) | 134.90(19) |
| C(35)-C(34)-C(33) | 121.0(3) | C(91)-O(7)-Y(1) | 135.7(2) |
| C(34)-C(35)-C(36) | 120.9(3) | C(91)-O(8)-Y(3) | 131.1(2) |
| C(37)-C(36)-C(35) | 120.2(3) | C(76)-O(9)-Y(3) | 90.78(18) |
| C(36)-C(37)-C(38) | 121.4(3) | C(76)-O(10)-Y(3) | 93.19(18) |
| Symmetry codes: #1 -x+1,-y+1,-z+2 | | | |

**Table S17.** Calculated energy levels (cm^−1^), ***g*** (*g_x_*, *g_y_*, *g_z_*) tensors and predominant *m_J_* values of the lowest eight Kramers doublets (KDs) of individual Dy^Ⅲ^ fragments for **1** and **1a** using CASSCF/RASSI-SO with the OpenMolcas.

| KDs | **1**-**Dy1** | | | **1**-**Dy2** | | | **1**-**Dy3** | | |
| --- | --- | --- | --- | --- | --- | --- | --- | --- | --- |
|  | *E* | ***g*** | *m_J_* | *E* | ***g*** | *m_J_* |  |  |  |
| 1 | 0.0 | 0.392  1.049  18.956 | ±15/2 | 0.0 | 0.614  2.282  17.738 | ±15/2 | 0.0 | 0.312  0.993  18.843 | ±15/2 |
| 2 | 31.0 | 0.098  1.052  16.253 | ±13/2 | 48.1 | 2.772  4.060  11.359 | ±13/2 | 52.0 | 1.711  2.684  15.238 | ±9/2 |
| 3 | 114.4 | 1.914  2.779  12.282 |  | 103.3 | 7.480  5.777  0.453 |  | 96.0 | 8.734  7.244  3.096 |  |
| 4 | 165.5 | 3.299  4.247  12.414 |  | 164.7 | 2.032  3.540  13.114 |  | 129.1 | 0.109  2.697  15.031 |  |
| 5 | 213.5 | 1.434  2.099  11.774 |  | 189.1 | 0.712  2.257  13.912 |  | 135.3 | 1.070  2.982  11.247 |  |
| 6 | 298.7 | 0.284  0.323  14.617 |  | 250.6 | 0.548  1.018  15.625 |  | 203.0 | 1.057  1.634  14.076 |  |
| 7 | 377.1 | 0.032  0.061  17.928 |  | 334.3 | 0.186  0.314  18.860 |  | 307.3 | 0.181  0.343  18.700 |  |
| 8 | 510.2 | 0.000  0.007  19.706 |  | 445.5 | 0.038  0.070  19.609 |  | 396.1 | 0.065  0.144  19.440 |  |
| KDs | **1-Dy4** | | | **1a-Dy1** | | | **1a-Dy2** | | |
|  | *E* | ***g*** | *m_J_* | *E* | ***g*** | *m_J_* | *E* | ***g*** | *m_J_* |
| 1 | 0.0 | 0.115  0.778  19.073 | ±15/2 | 0.0 | 0.320  0.806  18.624 | ±15/2 | 0.0 | 0.935  4.193  16.215 | ±15/2 |
| 2 | 27.2 | 0.324  0.867  18.467 | ±9/2 | 47.6 | 1.004  1.588  16.984 | ±9/2 | 38.0 | 2.408  3.657  9.102 | ±13/2 |
| 3 | 96.8 | 3.658  5.000  11.568 |  | 101.1 | 0.699  2.854  12.132 |  | 94.4 | 8.000  5.085  0.263 |  |
| 4 | 139.5 | 8.431  5.585  0.925 |  | 126.9 | 3.114  3.902  11.062 |  | 150.1 | 0.891  2.295  14.668 |  |
| 5 | 182.4 | 1.061  2.667  13.050 |  | 177.2 | 2.448  3.115  11.289 |  | 178.0 | 1.532  3.101  12.749 |  |
| 6 | 224.1 | 0.929  1.618  15.239 |  | 247.4 | 0.312  0.429  17.005 |  | 237.6 | 0.012  0.923  14.985 |  |
| 7 | 344.1 | 0.003  0.156  18.387 |  | 356.6 | 0.110  0.507  14.995 |  | 284.5 | 0.357  0.513  18.550 |  |
| 8 | 410.8 | 0.044  0.124  19.337 |  | 373.8 | 0.070  0.565  16.203 |  | 450.2 | 0.013  0.018  19.692 |  |
| KDs | **1a-Dy3** | | | **1a-Dy4** | | |  | | |
|  | *E* | ***g*** | *m_J_* | *E* | ***g*** | *m_J_* |  |  |  |
| 1 | 0.0 | 0.183  0.442  19.077 | ±15/2 | 0.0 | 0.495  1.908  18.115 | ±15/2 |  |  |  |
| 2 | 59.3 | 1.293  1.508  16.449 | ±13/2 | 17.5 | 0.826  1.126  16.797 | ±9/2 |  |  |  |
| 3 | 91.3 | 0.222  1.015  16.922 |  | 110.7 | 3.445  5.140  11.385 |  |  |  |  |
| 4 | 114.5 | 2.576  3.907  11.424 |  | 156.9 | 1.567  4.996  8.633 |  |  |  |  |
| 5 | 142.4 | 8.715  6.997  2.925 |  | 202.1 | 1.331  2.312  12.352 |  |  |  |  |
| 6 | 189.0 | 1.061  1.290  13.698 |  | 249.3 | 0.570  0.977  15.827 |  |  |  |  |
| 7 | 327.4 | 0.400  0.698  17.942 |  | 379.6 | 0.157  0.243  15.920 |  |  |  |  |
| 8 | 362.1 | 0.236  1.032  18.539 |  | 414.0 | 0.087  0.380  17.412 |  |  |  |  |

**Table S18.** Wave functions with definite projection of the total moment | *m_J_* > for the lowest eight KDs of individual Dy^Ⅲ^ fragments for **1** and **1a** using CASSCF/RASSI-SO with the OpenMolcas.

|  | *E*/cm^−1^ | wave functions |
| --- | --- | --- |
| **1**-**Dy1** | 0.0 | 90.7%\|±15/2> |
|  | 31.0 | 59.0%\|±13/2>+11.0%\|±11/2>+9.4%\|±9/2>+6.2%\|±5/2> |
| **1**-**Dy2** | 0.0 | 78.1%\|±15/2>+12.6%\|±11/2> |
|  | 48.1 | 23.8%\|±13/2>+22.6%\|±9/2>+16.7%\|±5/2>+11.0%\|±1/2> |
| **1**-**Dy3** | 0.0 | 89.3%\|±15/2> |
|  | 52.0 | 14.2%\|±13/2>+24.3%\|±9/2>+12.7%\|±7/2>+14.2%\|±5/2>+10.3%\|±3/2>+12.4%\|±1/2> |
| **1**-**Dy4** | 0.0 | 93.8%\|±15/2> |
|  | 27.2 | 11.1%\|±13/2>+18.5%\|±11/2>+18.8%\|±9/2>+13.0%\|±7/2>+12.7%\|±5/2>+11.2%\|±3/2> |
| **1a**-**Dy1** | 0.0 | 82.8%\|±15/2>+13.1%\|±11/2> |
|  | 47.6 | 14.2%\|±13/2>+31.5%\|±9/2>+15.7%\|±7/2>+16.5%\|±5/2>+8.3%\|±11/2> |
| **1a**-**Dy2** | 0.0 | 65.3%\|±15/2>+19.3%\|±11/2>+6.1%\|±7/2> |
|  | 38.0 | 10.4%\|±15/2>+36.9%\|±13/2>+20.2%\|±9/2>+13.6%\|±5/2>+9.5%\|±1/2> |
| **1a**-**Dy3** | 0.0 | 90.1%\|±15/2>+5.5%\|±11/2> |
|  | 59.3 | 35.1%\|±13/2>+17.4%\|±11/2>+22.9%\|±9/2>+7.1%\|±5/2>+5.7%\|±3/2> |
| **1a**-**Dy4** | 0.0 | 81.5%\|±15/2>+10.9%\|±11/2> |
|  | 17.5 | 22.8%\|±13/2>+13.3%\|±11/2>+30.1%\|±9/2>+11.3%\|±7/2>+9.2%\|±5/2>+8.1%\|±15/2> |

**Table S19.** Exchange energies *E* (cm^−1^), the energy differences between each exchange doublet Δ*_t_* (cm^−1^) and the main values of the *g_z_* for the lowest 8 and 24 exchange doublets arising from the exchange interactions on magnetic centers **1** and **1a**.

|  | **1** | | |
| --- | --- | --- | --- |
| Exchange doublets | *E* | Δ*_t_* | *g*_z_ |
| 0 | 0.000000000000 | 5.804×10^−5^ | 11.352 |
|  | 0.000058040242 |  |  |
| 1 | 0.064846180120 | 1.561×10^−5^ | 17.016 |
|  | 0.064861792418 |  |  |
| 2 | 0.260623200251 | 2.285×10^−5^ | 28.100 |
|  | 0.260646045784 |  |  |
| 3 | 0.420659441243 | 9.976×10^−4^ | 26.947 |
|  | 0.421657051588 |  |  |
| 4 | 0.425802215259 | 5.417×10^−4^ | 26.066 |
|  | 0.426343873568 |  |  |
| 5 | 0.437666535482 | 1.378×10^−3^ | 34.471 |
|  | 0.439044767528 |  |  |
| 6 | 0.640913056419 | 8.973×10^−5^ | 42.536 |
|  | 0.641002786104 |  |  |
| 7 | 1.589310199493 | 9.302×10^−6^ | 65.627 |
|  | 1.589319501063 |  |  |
|  | **1a** | | |
| Exchange doublets | *E* | Δ*_t_* | *g*_z_ |
| 0 | 0.000000000000 | 3.277×10^−6^ | 11.712 |
|  | 0.000003276751 |  |  |
| 1 | 0.027868199111 | 1.821×10^−6^ | 13.551 |
|  | 0.027870020199 |  |  |
| 2 | 0.104006823686 | 8.675×10^−6^ | 16.301 |
|  | 0.104007691234 |  |  |
| 3 | 0.379884934887 | 4.500×10^−5^ | 26.569 |
|  | 0.379929934965 |  |  |
| 4 | 0.431200347324 | 3.600×10^−4^ | 11.853 |
|  | 0.431560395086 |  |  |
| 5 | 0.452857782795 | 3.387×10^−4^ | 14.020 |
|  | 0.453196490021 |  |  |
| 6 | 0.544761314967 | 1.235×10^−4^ | 24.692 |
|  | 0.544884808440 |  |  |
| 7 | 0.553642492272 | 2.338×10^−4^ | 26.011 |
|  | 0.553876295764 |  |  |
| 8 | 0.560923826581 | 3.154×10^−4^ | 15.935 |
|  | 0.561239213967 |  |  |
| 9 | 0.693102329412 | 4.318×10^−6^ | 42.631 |
|  | 0.693106647216 |  |  |
| 10 | 0.838492857810 | 2.600×10^−4^ | 29.857 |
|  | 0.838752859259 |  |  |
| 11 | 0.891227891178 | 3.785×10^−5^ | 11.885 |
|  | 0.891265744021 |  |  |
| 12 | 0.915645225560 | 4.771×10^−5^ | 13.522 |
|  | 0.915692935923 |  |  |
| 13 | 0.980974091295 | 9.521×10^−4^ | 29.439 |
|  | 0.981926216136 |  |  |
| 14 | 0.991931351610 | 8.509×10^−4^ | 32.147 |
|  | 0.992782201925 |  |  |
| 15 | 1.006676713324 | 6.712×10^−5^ | 20.032 |
|  | 1.006743836528 |  |  |
| 16 | 1.139376293414 | 2.291×10^−4^ | 40.470 |
|  | 1.139605344121 |  |  |
| 17 | 1.287337990864 | 8.285×10^−7^ | 34.675 |
|  | 1.287338819317 |  |  |
| 18 | 1.416658910093 | 1.656×10^−6^ | 38.725 |
|  | 1.416660565726 |  |  |
| 19 | 1.431330751871 | 9.169×10^−7^ | 39.358 |
|  | 1.431331668784 |  |  |
| 20 | 1.586636957075 | 9.106×10^−7^ | 38.550 |
|  | 1.586637867628 |  |  |
| 21 | 1.982512644841 | 6.093×10^−8^ | 63.000 |
|  | 1.982512705770 |  |  |
| 22 | 2.424701662233 | 1.314×10^−5^ | 66.550 |
|  | 2.424714802982 |  |  |
| 23 | 2.868837973922 | 3.745×10^−8^ | 69.980 |
|  | 2.868838011369 |  |  |
